# Supplementary material for: Impact of the first year of COVID-19 vaccination strategy in Brazil: an ecological study
Source: BMJ Open. 2024 Jul 4;14(7):e072314. doi: 10.1136/bmjopen-2023-072314 (PMC11227766; doi:10.1136/bmjopen-2023-072314)
Supplement: Supplementary data [file bmjopen-2023-072314supp001.pdf]

Supplementary Material

Table of contents

Figure S1. (A) Deaths per 100.000 pop stratified by age group – The vertical dashed line refers to the start of the vaccination campaign. (B) Estimated effect for each age group before and after the beginning the vaccination campaign. Effects were obtained as the Rate Ratio (RR) and their respective 95% confidence intervals. 3
Figure S2. (A) First dose vaccination coverage stratified by age group – first vertical dashed line on the left refers to the start of the vaccination campaign for each age group, the horizontal dashed line indicates the 75% vaccination coverage target by the World Health Organization (WHO). (B) Second or single vaccination coverage stratified by age group. 3
Figure S3. (A) Estimated effect of the vaccination campaign in each age groups comparing to 70+ age group. (B) Estimated effect of the vaccination campaign in each age groups comparing to 20-49 age group. Effects were obtained as the Rate Ratio (RR) and their respective 95% confidence intervals. The vertical dashed line refers to the start of the vaccination campaign on January 17, 2021. Our analysis considered six months before the vaccination roll-out as the baseline period and used the national mortality rates as reference in a Negative Binomial Regression model. 4

Table S1. Description of the data sources used in this study 2
Table S2. Monthly average of the mortality ratios in Brazil, in 2021, stratified by age group. 5
Table S3. Number of reported deaths and first dose vaccine coverage per month in during the COVID-19 vaccination campaign in Brazil (started on January 17, 2021). 6
Table S4. Second or single vaccination coverage during the vaccination roll-out in Brazil, in 2021, stratified by age group. 7
Table S5. Monthly and global estimated effect of the vaccination campaign with their respective 95% confidence intervals stratified by age group. 8
Table S6. Monthly estimated effect of the vaccination campaign with their respective 95% confidence intervals stratified by age group (70+ reference). 8
Table S7. Monthly estimated effect of the vaccination campaign with their respective 95% confidence intervals stratified by age group (20-49 reference). 8
Table S8. Monthly estimated effect of the vaccination campaign with their respective 95% confidence intervals stratified by age group. 9

Aguilar S, *et al. BMJ Open* 2024; 14:e072314. doi: 10.1136/bmjopen-2023-072314

Table S1. Description of the data sources used in this study

Data	Source	Source address	Version	Date exported
COVID-19 Vaccination doses	National Immunisation Program Information System,"SI-PNI"	*OpenDataSUS* repository: https://opendatasus.saude.gov.br/dataset/covid-19-vacinacao	21/07/2022	21/07/2022
Brazilian population estimates (per age, sex, and municipalities)	Health Surveillance Secretary from the Brazilian Ministry of Health	Official website: http://tabnet.datasus.gov.br/cgi/tabcgi.exe?popsvs/cnv/popbr.def	2020	07/02/2022
COVID-19 hospitalized cases	Influenza Epidemiological Surveillance Information System, "SIVEP-Gripe"	*OpenDataSUS* repository: https://opendatasus.saude.gov.br/dataset/bd-srag-2020 https://opendatasus.saude.gov.br/dataset/srag-2021-e-2022	21/07/2022	21/07/2022
Life expectancy at birth, total (years) - Brazil	World Bank	Official website: https://data.worldbank.org/indicator/SP.DYN.LE00.IN?locations=BR&most_recent_year_desc=true	2019	25/07/2022

COVID-19: Disease caused by the SARS-CoV-2 virus.

Figure S1. (A) Deaths per 100.000 pop stratified by age group – The vertical dashed line refers to the start of the vaccination campaign. (B) Estimated effect for each age group before and after the beginning the vaccination campaign. Effects were obtained as the Rate Ratio (RR) and their respective 95% confidence intervals.

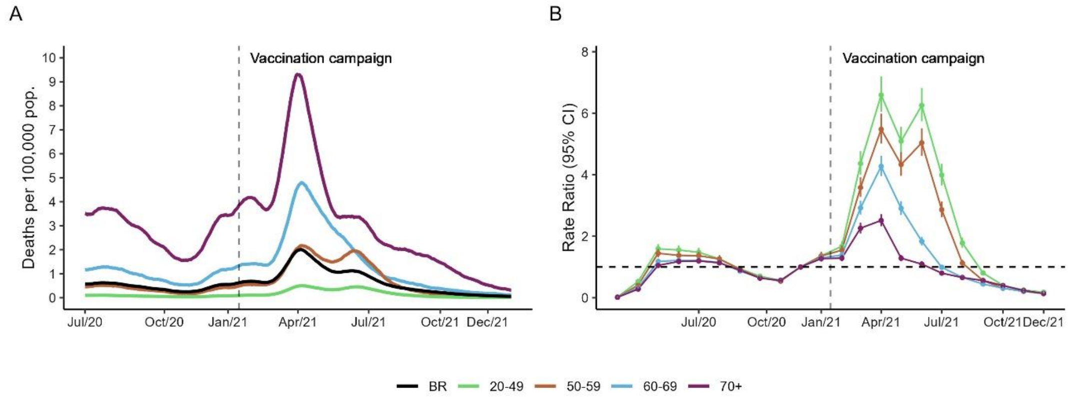

Figure S2. (A) First dose vaccination coverage stratified by age group – first vertical dashed line on the left refers to the start of the vaccination campaign for each age group, the horizontal dashed line indicates the 75% vaccination coverage target by the World Health Organization (WHO). (B) Second or single vaccination coverage stratified by age group.

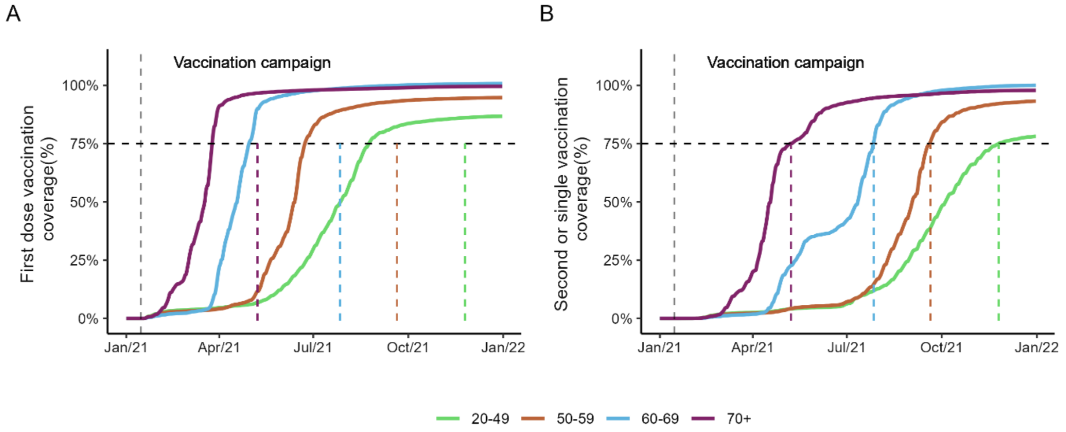

Aguilar S, *et al. BMJ Open* 2024; 14:e072314. doi: 10.1136/bmjopen-2023-072314

Figure S3. (A) Estimated effect of the vaccination campaign in each age groups comparing to 70+ age group. (B) Estimated effect of the vaccination campaign in each age groups comparing to 20-49 age group. Effects were obtained as the Rate Ratio (RR) and their respective 95% confidence intervals. The vertical dashed line refers to the start of the vaccination campaign on January 17, 2021. Our analysis considered six months before the vaccination roll-out as the baseline period and used the national mortality rates as reference in a Negative Binomial Regression model.

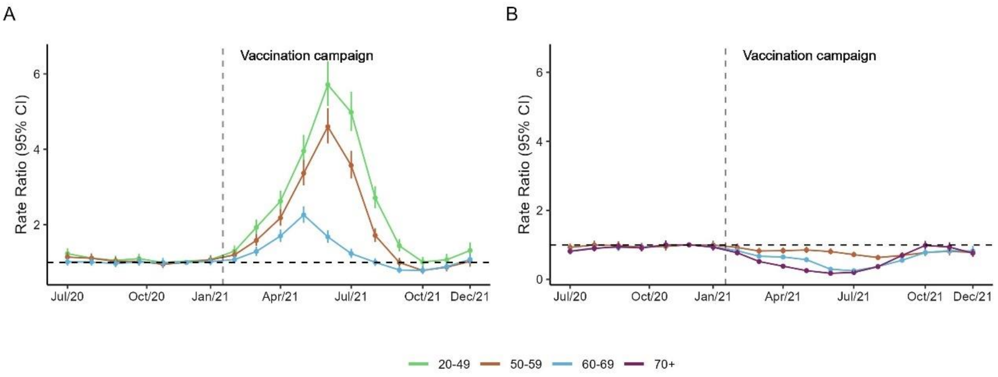

Table S2. Monthly average of the mortality ratios in Brazil, in 2021, stratified by age group.

Age group	Jan/21	Feb/21	Mar/21	Apr/21	Mai/21	Jun/21	Jul/21	Aug/21	Sep/21	Oct/21	Nov/21	Dec/21
20 – 49	0.15	0.17	0.22	0.26	0.31	0.41	0.41	0.31	0.2	0.16	0.16	0.18
50 – 59	0.79	0.85	0.96	1.14	1.38	1.72	1.53	1.03	0.75	0.63	0.69	0.74
60 – 69	2.12	2.12	2.22	2.48	2.58	1.74	1.52	1.7	1.7	1.81	1.95	2.16
70+	6.16	5.87	5.21	4.29	3.38	3.11	3.69	5.1	6.35	6.77	6.48	5.96

Table S3. Number of reported deaths and first dose vaccine coverage per month in during the COVID-19 vaccination campaign in Brazil (started on January 17, 2021).

Age group	Monthly Average Baseline*	Baseline*	Jan/21	Feb/21	Mar/21	Apr/21	Mai/21	Jun/21	Jul/21	Aug/21	Sep/21	Oct/21	Nov/21	Dec/21
Monthly reported deaths														
BR	20,627	123,763	29,346	28,141	62,603	79,939	53,772	48,416	31,293	18,485	12,164	8,254	4,958	3,223
20 – 49	2,050	12,298 (10 %)	2,842 (10 %)	3,149 (11 %)	9,099 (15 %)	13,299 (17 %)	10,617 (20 %)	12,620 (26 %)	8,316 (27 %)	3,714 (20 %)	1,626 (13 %)	832 (10 %)	506 (10 %)	372 (12 %)
50 – 59	2,545	15,269 (12 %)	3,641 (12 %)	3,748 (13 %)	9,611 (15 %)	14,224 (18 %)	11,623 (22 %)	13,082 (27 %)	7,677 (25 %)	3,022 (16 %)	1,449 (12 %)	830 (10 %)	531 (11 %)	374 (12 %)
60 – 69	4,731	28,385 (23 %)	6,845 (23 %)	6,588 (23 %)	15,385 (25 %)	21,775 (27 %)	15,319 (28 %)	9,363 (19 %)	5,210 (17 %)	3,471 (19 %)	2,276 (19 %)	1,634 (20 %)	1,063 (21 %)	770 (24 %)
70+	11,302	67,811 (55 %)	16,018 (55 %)	14,656 (52 %)	28,508 (46 %)	30,641 (38 %)	16,213 (30 %)	13,351 (28 %)	10,090 (32 %)	8,278 (45 %)	6,813 (56 %)	4,958 (60 %)	2,858 (58 %)	1,707 (53 %)
Monthly average mortality rates per 100,000 people														
BR		0.44	0.62	0.66	1.33	1.76	1.14	1.06	0.67	0.39	0.27	0.18	0.11	0.07
20 – 49		0.07	0.09	0.12	0.30	0.45	0.35	0.43	0.27	0.12	0.06	0.03	0.02	0.01
50 – 59		0.35	0.49	0.56	1.30	1.99	1.57	1.83	1.04	0.41	0.20	0.11	0.07	0.05
60 – 69		0.92	1.32	1.41	2.97	4.34	2.95	1.87	1.00	0.67	0.45	0.32	0.21	0.15
70+		2.73	3.84	3.89	6.83	7.59	3.88	3.31	2.42	1.98	1.69	1.19	0.71	0.41
Vaccine coverage# (%)														
BR			1.82	4.74	13.75	21.92	32.22	50.82	67.72	84.82	88.32	89.58	90.3	90.67
20 – 49			1.99	3.61	4.56	5.98	12.55	28.85	52.75	78.55	83.55	85.26	86.23	86.72
50 – 59			1.65	3.06	4.08	7.64	31.84	81.34	89.73	92.36	93.57	94.16	94.53	94.75
60 – 69			1.05	2.28	19.58	75.48	95.28	97.61	98.79	99.6	100.09	100.4	100.6	100.72
70+			1.85	18.95	90.35	96.32	97.4	97.99	98.38	98.72	99.07	99.4	99.53	99.61

*Baseline period corresponds to the six-month period (July to December 2020), prior to the start of the vaccination campaign. For monthly reported deaths, the baseline is the sum of the six months deaths.

#At least one dose.

Table S4. Second or single vaccination coverage during the vaccination roll-out in Brazil, in 2021, stratified by age group.

Age group	Jan/21	Feb/21	Mar/21	Apr/21	Mai/21	Jun/21	Jul/21	Aug/21	Sep/21	Oct/21	Nov/21	Dec/21
Vaccine coverage (%)												
BR	0	1.56	3.86	11.37	15.26	17.78	28.38	42.88	62.88	76.18	82.88	84.55
20 – 49	0	1.7	2.54	3.7	4.65	6.05	12.74	26.04	47.34	66.04	75.76	78.08
50 – 59	0	1.45	2.19	3.4	5.21	6.95	17.05	46.05	83.75	90.42	92.44	93.29
60 – 69	0	0.87	1.79	18.39	35.29	41.64	83.04	94.74	97.78	98.94	99.71	100
70+	0.01	1.67	19.17	72.57	85.27	92.52	94.75	95.68	96.59	97.27	97.69	97.83

Table S5. Monthly and global estimated effect of the vaccination campaign with their respective 95% confidence intervals stratified by age group.

Age group	Global	Jan/21	Feb/21	Mar/21	Apr/21	Mai/21	Jun/21	Jul/21	Aug/21	Sep/21	Oct/21	Nov/21	Dec/21
20 – 49	1.75 (1.44 - 2.12)	1.05 (0.95 - 1.17)	1.21 (1.09 - 1.35)	1.56 (1.41 - 1.73)	1.78 (1.61 - 1.97)	2.10 (1.90 - 2.32)	2.75 (2.48 - 3.04)	2.80 (2.53 - 3.10)	2.15 (1.93 - 2.38)	1.44 (1.29 - 1.62)	1.09 (0.97 - 1.24)	1.11 (0.97 - 1.27)	1.25 (1.08 - 1.44)
50 – 59	1.47 (1.21 - 1.78)	1.05 (0.94 - 1.16)	1.12 (1.01 - 1.24)	1.28 (1.16 - 1.42)	1.48 (1.34 - 1.64)	1.79 (1.62 - 1.97)	2.21 (2.00 - 2.44)	2.01 (1.82 - 2.22)	1.36 (1.22 - 1.51)	1.00 (0.90 - 1.12)	0.85 (0.75 - 0.96)	0.90 (0.79 - 1.03)	0.98 (0.84 - 1.13)
60 – 69	1.01 (0.84 - 1.23)	1.00 (0.91 - 1.10)	1.00 (0.91 - 1.11)	1.05 (0.95 - 1.15)	1.15 (1.05 - 1.23)	1.20 (1.09 - 1.27)	0.81 (0.73 - 1.32)	0.70 (0.63 - 0.71)	0.79 (0.72 - 0.88)	0.80 (0.72 - 0.89)	0.85 (0.76 - 0.95)	0.92 (0.82 - 1.03)	1.02 (0.91 - 1.16)
70+	0.73 (0.60 - 0.88)	0.98 (0.89 - 1.08)	0.93 (0.85 - 1.03)	0.81 (0.74 - 0.89)	0.68 (0.62 - 0.75)	0.53 (0.48 - 0.58)	0.48 (0.43 - 0.53)	0.56 (0.51 - 0.62)	0.79 (0.72 - 0.87)	1.00 (0.91 - 1.10)	1.08 (0.98 - 1.19)	1.04 (0.93 - 1.15)	0.95 (0.85 - 1.06)

Table S6. Monthly estimated effect of the vaccination campaign with their respective 95% confidence intervals stratified by age group (70+ reference).

Age group	Jan/21	Feb/21	Mar/21	Apr/21	Mai/21	Jun/21	Jul/21	Aug/21	Sep/21	Oct/21	Nov/21	Dec/21
20 – 49	1.07 (0.96 - 1.19)	1.30 (1.16 - 1.45)	1.93 (1.74 - 2.14)	2.62 (2.37 - 2.91)	3.96 (3.57 - 4.39)	5.71 (5.15 - 6.34)	4.98 (4.49 - 5.53)	2.71 (2.44 - 3.02)	1.44 (1.29 - 1.62)	1.01 (0.90 - 1.15)	1.07 (0.93 - 1.23)	1.32 (1.13 - 1.53)
50 – 59	1.07 (0.96 - 1.19)	1.20 (1.08 - 1.34)	1.58 (1.43 - 1.75)	2.18 (1.97 - 2.41)	3.36 (3.05 - 3.72)	4.60 (4.16 - 5.09)	3.57 (3.23 - 3.96)	1.71 (1.54 - 1.91)	1.00 (0.89 - 1.12)	0.79 (0.70 - 0.89)	0.87 (0.76 - 1.00)	1.03 (0.89 - 1.19)
60 – 69	1.02 (0.93 - 1.13)	1.08 (0.97 - 1.19)	1.29 (1.17 - 1.42)	1.70 (1.54 - 1.87)	2.26 (2.05 - 2.49)	1.68 (1.52 - 1.85)	1.24 (1.18 - 1.37)	1.00 (0.91 - 1.11)	0.80 (0.72 - 0.89)	0.79 (0.71 - 0.88)	0.89 (0.79 - 1.00)	1.08 (0.95 - 1.23)
70+ (REF)	-	-	-	-	-	-	-	-	-	-	-	-

Table S7. Monthly estimated effect of the vaccination campaign with their respective 95% confidence intervals stratified by age group (20-49 reference).

Age group	Jan/21	Feb/21	Mar/21	Apr/21	Mai/21	Jun/21	Jul/21	Aug/21	Sep/21	Oct/21	Nov/21	Dec/21
20 – 49 (REF)	-	-	-	-	-	-	-	-	-	-	-	-
50 – 59	0.99 (0.89 - 1.19)	0.92 (0.82 - 1.04)	0.82 (0.74 - 0.92)	0.83 (0.75 - 0.93)	0.85 (0.76 - 0.95)	0.81 (0.72 - 0.90)	0.71 (0.64 - 0.80)	0.63 (0.56 - 0.71)	0.69 (0.61 - 0.79)	0.77 (0.67 - 0.89)	0.81 (0.69 - 0.96)	0.78 (0.65 - 0.93)
60 – 69	0.95 (0.85 - 1.07)	0.82 (0.74 - 0.93)	0.67 (0.60 - 0.74)	0.65 (0.58 - 0.72)	0.57 (0.51 - 0.63)	0.29 (0.26 - 0.33)	0.24 (0.22 - 0.28)	0.37 (0.33 - 0.41)	0.55 (0.49 - 0.63)	0.78 (0.68 - 0.89)	0.83 (0.72 - 0.96)	0.82 (0.70 - 0.96)
70+	0.93 (0.84 - 1.04)	0.76 (0.69 - 0.86)	0.52 (0.47 - 0.57)	0.38 (0.34 - 0.42)	0.25 (0.23 - 0.28)	0.18 (0.16 - 0.19)	0.20 (0.18 - 0.22)	0.37 (0.33 - 0.41)	0.69 (0.62 - 0.78)	0.99 (0.87 - 1.12)	0.93 (0.81 - 1.07)	0.75 (0.65 - 0.88)

Table S8. Monthly estimated effect of the vaccination campaign with their respective 95% confidence intervals stratified by age group.

Age group	Jan/21	Feb/21	Mar/21	Apr/21	Mai/21	Jun/21	Jul/21	Aug/21	Sep/21	Oct/21	Nov/21	Dec/21
20 – 49	1.36 (1.24 - 1.50)	1.67 (1.52 - 1.84)	4.36 (4.00 - 4.76)	6.59 (6.04 - 7.19)	5.09 (4.67 - 5.56)	6.25 (5.73 - 6.83)	3.99 (3.68 - 4.35)	1.78 (1.63 - 1.95)	0.81 (0.73 - 0.89)	0.40 (0.36 - 0.44)	0.25 (0.22 - 0.28)	0.18 (0.16 - 0.20)
50 – 59	1.35 (1.23 - 1.49)	1.55 (1.41 - 1.70)	3.58 (3.28 - 3.91)	5.48 (5.01 - 5.99)	4.33 (3.97 - 4.73)	5.04 (4.61 - 5.51)	2.86 (2.62 - 3.13)	1.13 (1.03 - 1.24)	0.56 (0.50 - 0.62)	0.31 (0.28 - 0.35)	0.20 (0.18 - 0.23)	0.14 (0.12 - 0.15)
60 – 69	1.29 (1.20 - 1.41)	1.38 (1.27 - 1.50)	2.92 (2.70 - 3.15)	4.27 (3.96 - 4.61)	2.91 (2.69 - 3.14)	1.84 (1.70 - 1.99)	0.99 (0.91 - 1.07)	0.66 (0.61 - 0.72)	0.45 (0.41 - 0.49)	0.31 (0.28 - 0.34)	0.21 (0.19 - 0.23)	0.15 (0.13 - 0.16)
70+	1.28 (1.18 - 1.37)	1.29 (1.19 - 1.39)	2.26 (2.09 - 2.44)	2.51 (2.32 - 2.72)	1.29 (1.19 - 1.39)	1.09 (1.01 - 1.18)	0.80 (0.74 - 0.87)	0.66 (0.61 - 0.71)	0.56 (0.52 - 0.61)	0.39 (0.36 - 0.43)	0.23 (0.22 - 0.26)	0.14 (0.12 - 0.15)

Aguilar S, *et al. BMJ Open* 2024; 14:e072314. doi: 10.1136/bmjopen-2023-072314
