# Supplementary material for: Impact of the first year of COVID-19 vaccination strategy in Brazil: an ecological study
Source: BMJ Open. 2024 Jul 4;14(7):e072314. doi: 10.1136/bmjopen-2023-072314 (PMC11227766; doi:10.1136/bmjopen-2023-072314)
Supplement: Author's manuscript [file bmjopen-2023-072314.draft_revisions.pdf]

BMJ Open

BMJ Open is committed to open peer review. As part of this commitment we make the peer review history of every article we publish publicly available.

When an article is published we post the peer reviewers' comments and the authors' responses online. We also post the versions of the paper that were used during peer review. These are the versions that the peer review comments apply to.

The versions of the paper that follow are the versions that were submitted during the peer review process. They are not the versions of record or the final published versions. They should not be cited or distributed as the published version of this manuscript.

BMJ Open is an open access journal and the full, final, typeset and author-corrected version of record of the manuscript is available on our site with no access controls, subscription charges or pay-per-view fees (http://bmjopen.bmj.com).

If you have any questions on BMJ Open's open peer review process please email info.bmjopen@bmj.com

BMJ Open

The impact of the first year of COVID-19 vaccination strategy in Brazil: An ecological study

Journal:	*BMJ Open*
Manuscript ID	bmjopen-2023-072314
Article Type:	Original research
Date Submitted by the Author:	30-Jan-2023
Complete List of Authors:	Aguilar, Soraida; Pontifical Catholic University of Rio de Janeiro, Department of Industrial Engineering<br>Bastos, Leonardo S. L.; Pontifical Catholic University of Rio de Janeiro, Department of Industrial Engineering<br>Maçaira, Paula; Pontifical Catholic University of Rio de Janeiro, Department of Industrial Engineering<br>Baião, Fernanda; Pontifical Catholic University of Rio de Janeiro, Department of Industrial Engineering<br>Simões, Paulo; Pontifical Catholic University of Rio de Janeiro, Department of Industrial Engineering<br>Cerbino-Neto, José; Oswaldo Cruz Foundation<br>Ranzani, Otavio; ISGlobal<br>Hamacher, Silvio; Pontifical Catholic University of Rio de Janeiro, Department of Industrial Engineering<br>Bozza, Fernando; D'Or Institute for Research and Education (IDOR)
Keywords:	COVID-19, Public health < INFECTIOUS DISEASES, EPIDEMIOLOGY

SCHOLARONE™
Manuscripts

BMJ

I, the Submitting Author has the right to grant and does grant on behalf of all authors of the Work (as defined in the below author licence), an exclusive licence and/or a non-exclusive licence for contributions from authors who are: i) UK Crown employees; ii) where BMJ has agreed a CC-BY licence shall apply, and/or iii) in accordance with the terms applicable for US Federal Government officers or employees acting as part of their official duties; on a worldwide, perpetual, irrevocable, royalty-free basis to BMJ Publishing Group Ltd ("BMJ") its licensees and where the relevant Journal is co-owned by BMJ to the co-owners of the Journal, to publish the Work in this journal and any other BMJ products and to exploit all rights, as set out in our licence.

The Submitting Author accepts and understands that any supply made under these terms is made by BMJ to the Submitting Author unless you are acting as an employee on behalf of your employer or a postgraduate student of an affiliated institution which is paying any applicable article publishing charge ("APC") for Open Access articles. Where the Submitting Author wishes to make the Work available on an Open Access basis (and intends to pay the relevant APC), the terms of reuse of such Open Access shall be governed by a Creative Commons licence – details of these licences and which Creative Commons licence will apply to this Work are set out in our licence referred to above.

Other than as permitted in any relevant BMJ Author's Self Archiving Policies, I confirm this Work has not been accepted for publication elsewhere, is not being considered for publication elsewhere and does not duplicate material already published. I confirm all authors consent to publication of this Work and authorise the granting of this licence.

For peer review only

The impact of the first year of COVID-19 vaccination strategy in Brazil: An ecological study

Soraida Aguilar, PhD[1]; Leonardo S. L. Bastos, PhD[1,2]; Paula Maçaira, PhD[1]; Fernanda Baião, PhD[1]; Paulo Simões, MSc[1]; José Cerbino-Neto[3], MD, PhD; Otavio T. Ranzani, MD, PhD[4,5]; Silvio Hamacher, PhD[1,2]; Fernando A. Bozza, MD, PhD[3,6,*]

1. Department of Industrial Engineering (DEI), Pontifical Catholic University of Rio de Janeiro (PUC-Rio), Rio de Janeiro, RJ, 22451-900, Brazil

2. Tecgraf Institute, Pontifical Catholic University of Rio de Janeiro (PUC-Rio), Rio de Janeiro, RJ, 22451-900, Brazil

3. National Institute of Infectious Disease Evandro Chagas (INI), Oswaldo Cruz Foundation (FIOCRUZ), Rio de Janeiro, RJ, 21045-900, Brazil

4. Barcelona Institute for Global Health, ISGlobal, Barcelona, 08003, Spain.

5. Pulmonary Division, Heart Institute (InCor), Hospital das Clinicas HCFMUSP, Faculdade de Medicina da Universidade de São Paulo, São Paulo, Brazil

6. D'Or Institute for Research and Education (IDOR), Rio de Janeiro, RJ, 22281-100, Brazil

Corresponding author:

***Fernando A. Bozza, MD, PhD.**, Laboratório de Pesquisa Clínica em Medicina Intensiva, Instituto Nacional de Infectologia Evandro Chagas (INI), Fundação Oswaldo Cruz (FIOCRUZ), Av. Brasil 4365, Rio De Janeiro, RJ, 21045-900, Brazil.

Email: fernando.bozza@ini.fiocruz.br or bozza.fernando@gmail.com

For peer review only

Abstract

Objectives: No consensus exists about the best COVID-19 vaccination strategy to be adopted by low-and-middle-income countries. Brazil adopted an age-based calendar strategy to reduce mortality and the burden over the healthcare system. This study evaluates the impact of the vaccination campaign in Brazil on the progression of the COVID-19 reported deaths.

Methods: This ecological study analyses the dynamic of vaccination coverage and COVID-19 deaths in hospital adults (≥20 years) during the first year of the COVID-19 vaccination roll-out (January-December, 2021) using nationwide data (DATASUS). We stratified the adult population into 20-49, 50-59, 60-69, and 70+ years. The dynamic effect of the vaccination campaign on mortality rates was estimated using Differences-in-Differences. The prevented and preventable deaths (observed deaths higher than expected), and Potential Years of Life Lost (PYLL), for each age group were obtained in a counterfactual analysis.

Results: During the first year of COVID-19 vaccination 266,153,517 doses were administered, achieving 91% first-dose coverage. 380,594 deaths were reported, being 154,091 (40%) in 70+, and 136,804 (36%) from 50-59 or 20-49 years. The mortality rates of 70+ decreased 52% (RRR [95% CI]: 0.48 [0.43-0.53]) in 6 months, whereas rates for 20-49 were still increasing due to the low coverage (52%). The vaccination roll-out strategy prevented 59,618 deaths, 53,088 (89%) from those aged 70+ years. However, the strategy did not prevent 54,797 deaths, 85% from those under 60 years, being 26,344 (45%) only in 20-49, corresponding to 1,589,271 PYLL, being 1,080,104 PYLL (68%) from those aged 20-49 years.

Conclusion: The adopted aged-based calendar vaccination strategy initially reduced the mortality in the oldest but did not significantly prevent the deaths of the youngest. Countries with high burden, limited vaccine supply, and young population should consider other factors besides age to prioritise who to vaccine first.

For peer review only

Strengths and limitations of this study

- We evaluated the impact of the COVID-19 vaccination strategy in Brazil on the number of reported deaths during the campaign's first year. In 2021, 266,153,517 COVID-19 vaccine doses were administered in adults with 91% vaccination coverage.
- The vaccination strategy followed an age calendar and prioritised the high-risk groups and following an age-based calendar, the mortality rates of the elderly population reduced earlier compared to the young population.
- Alternative strategies to an age-based calendar are necessary for countries with low-resource and young populations. These strategies should consider the local burden, vaccination supply and speed, vaccine hesitancy, and socioeconomic vulnerability.
- Non-pharmacological actions that may affect the progression of deaths and mortality rates and were not considered in the analysis.
- This ecological study analysed notified data in the SIVEP-Gripe, what limits the assessment of all COVID-19 deaths and excess mortality in the country.

For peer review only

Introduction

The COVID-19 pandemic imposed unprecedented challenges worldwide. In 2020, the pandemic in Brazil quickly spread across the different states, with distinct patterns and burdens due to the disparities in health assistance, income, and a lack of a unified national strategy to face the pandemic.[1,2] With the emergence and dominance of SARS-CoV-2-Variants of Concern (VOC)[3] at the end of 2020,[4] characterized by high transmissibility,[4] immune escape,[5,6] and increased disease severity, substantial pressure was imposed on the health system. At the beginning of 2021, when the vaccination campaign started in the country, a new surge – the second COVID-19 Gamma wave - was still rampant, with higher mortality than the first.[3]

Countries have used different strategies to define who vaccinates first.[7,8] Several studies evaluated the impact of vaccination campaigns on the infections, hospitalizations and saving lives principally, in high-income countries.[8–11] In Brazil, the National Immunization Program (PNI), responsible for providing the vaccination guidelines, is part of the Brazilian universal health system (*Sistema Unico de Saúde – SUS*) and has been recognized as a model of vaccination campaigns and population adhesion worldwide.[12] The Brazilian COVID-19 vaccination campaign, which started on January 17, 2021, prioritized high-risk groups and considered an age-based calendar beginning with the elderly (85 years or more).[13] Vaccine distribution accomplished the National guidelines centralizing the distribution of vaccine doses to the states and municipalities.

It is still unclear how to best prioritize groups for mass vaccination during pandemics/outbreaks such as COVID-19, especially in low-and-middle-income countries (LMIC) with a high burden of the disease and a young population. Hence, we performed an ecological study to evaluate the impact of the vaccination campaign in Brazil on the progression of the COVID-19 reported deaths. We estimated the number of prevented and preventable deaths during the campaign stratified by age groups.

Methods

Study Design and Participants

We conducted an ecological study to analyse the progression of COVID-19 reported deaths and mortality rates before and during the first year of the vaccination campaign roll-out in Brazil.

Data on reported deaths were obtained from the Influenza Epidemiological Surveillance Information System (*Sistema de Informação de Vigilância Epidemiológica da Gripe*, SIVEP-Gripe), a nationwide surveillance open-access database used to monitor severe acute respiratory infections (SARI) in Brazil. SIVEP-Gripe has been the primary source of COVID-19 reported deaths

For peer review only

in the country, covering 80% of Brazilian municipalities, with a confirmed case in Brazil comprising 96% of the population.[2] Each register contains information about the individual's demographics, self-reported symptoms, comorbidities, hospital and ICU admission and ventilatory support, in-hospital outcome (death or discharge), and dates of symptom onset, hospital admission, and ICU admission. We included all adult participants (≥20 years old) with SARS-CoV-2-confirmed infection (RT-PCR or clinical-epidemiological criteria) whose death was reported between February 27, 2020, on December 31, 2021.

Data on COVID-19 vaccination coverage was obtained from the Brazilian National Immunisation Program database (SI-PNI). The SI-PNI contains information on the individual's demographics (age, sex, and place of residence), vaccine dose and platform, and place and date of dose administration. We included all doses (first, second or single) administered to adults (≥20 years old) between January 17, 2021 (the start of the vaccination campaign) and December 31, 2021. Finally, we used the 2020 Population estimates provided by the Brazilian Ministry of Health stratified by age.

Data was publicly available, anonymized, and de-identified. Following ethically agreed principles on open data, this analysis did not require ethical approval in Brazil. Information on the data sources is in appendix p 2.

Patient and public involvement

No patient involved.

COVID-19 Vaccination Campaign Roll-Out in Brazil

On January 17, 2021, the Brazilian national immunisation program (PNI) initiated the vaccination campaign for COVID-19. Vaccine doses were freely distributed to states and municipalities, which managed the administration according to the availability of resources. The COVID-19 vaccination campaign strategy followed a national age-based calendar during the whole period, with some local adaptations. It started in parallel vaccinating those aged 90+, indigenous, healthcare workers, and high-risk groups based on comorbidities, then followed exclusively age-based calendar. Four vaccine platforms were considered in the country: two doses of Sinovac-CoronaVac, ChAdOx1-S/nCoV-19 (Oxford-AstraZeneca), or BNT162b2 (Pfizer/BioNTech); or Ad26.COV2.S (Janssen) single-dose.

For peer review only

Outcomes

Our primary outcome was the estimated number of prevented COVID-19 deaths. Secondary outcomes comprise preventable deaths, the potential years of life lost (PYLL), the mortality rates per 100,000 population, and the first dose and second or single dose vaccination coverage. All quantities, rates, and estimates were evaluated temporally for the whole country and stratified by age groups.

Data Analysis

We described continuous variables using medians and interquartile range (IQR) or means and standard deviations (SDs) when applicable, and frequency and proportions for categorical variables. The analysis was pre-specified, and the sample size was pragmatic (SIVEP-Gripe database). All the analysis considered complete data, and no imputation of missing values was performed.

We evaluated the progression of COVID-19 deaths before and during the vaccination campaign (February 26, 2020, to December 31, 2021) for the whole country and stratified by age groups. We generated the time series of daily reported deaths and mortality rates using the 15-day moving average to reduce the effects of variability in the notification process. Changes in the representativeness of age groups in the deaths were assessed by calculating the proportion of each group in the daily count. In addition, vaccination coverage of at least one dose ("partially vaccinated") and two or single doses ("fully vaccinated") was calculated as the number of doses divided by the corresponding estimated population.

To estimate the impact of the vaccination campaign strategy on the evolution of daily reported mortality rates for each age group (20-49, 50-59, 60-69, and 70+), we performed a three-stage analysis.

First, we estimated the dynamic effects of the vaccination campaign using a differences-in-differences (DID) analysis.[14] DID is a causal modelling approach used for impact evaluation by comparing treatment and control groups, before and after an intervention, under non-experimental settings (e.g., non-randomized data).[15] Each age group was considered the "treatment group" and the start of the vaccination roll-out was the intervention onset. We considered the six months previous vaccination roll-out as the "before intervention" period (July to December 2020). The control group is represented by the national age-adjusted mortality rates in Brazil as a surrogate of the pandemic progression in the country during the vaccination

For peer review only

campaign. We compared the daily mortality rates of each age group and the control group using the Rate Ratio (RR).

Afterwards, we applied a Negative Binomial Regression model to estimate the intervention effect. The response variable was the reported deaths, and the covariates were the intervention (age) / control group, the intervention period indicator, their interaction, and the population as the offset variable. To obtain the dynamic effects for each age group, we split the intervention period variable into separate indicators, one for each month. The intervention effect was calculated as the exponentiated coefficient of the interaction terms, defined as the ratio of rate ratios (RRR)[16] or the DID estimator, and their corresponding 95% confidence intervals.

Subsequently, we quantified the impact of the vaccination strategy in terms of prevented and preventable deaths for each age group using a counterfactual analysis.[17] Using a Linear Regression model, we predicted the mortality rate of an age group (response variable, y_i), based on the national age-adjusted mortality rates in the country (predictor variable, X_i).[17] The model is expressed by:

$$y_i = \beta_0 + \beta_1 X_i + \varepsilon_i \qquad (1)$$

Where ε_i denotes the error term. Although the national age-adjusted mortality rates in the country do not represent strictly a scenario without vaccination, we could obtain the number of expected deaths considering this progression as being the dynamic pandemic progression and the vaccination roll-out as the speed and effectiveness in the decrease of deaths for each age group.

We considered the start of the vaccination roll-out as the date of intervention onset (January 17, 2021). Hence, we fit the model using data previous to the vaccination roll-out obtained the number of predicted mortality rates for each age group in the following period. We multiplied the predicted mortality rates with the total population to obtain the number of expected deaths. When comparing the number of expected to observed deaths for each age group, we considered “prevented deaths” as when the observed deaths were lower than the expected deaths; otherwise, we attained “preventable deaths”.

Furthermore, we estimated the corresponding number of prevented and preventable Potential Years of Life Lost (PYLL) for each age group, considering the midpoint of each age group and a life expectancy of 76 years for Brazil in 2019.[18] All the analyses were performed in R 4.2.1. We followed STROBE recommendations.

For peer review only

Role of the Funding Source

The funders had no role in any decision about the manuscript. All authors had full access to all the data in the study, SA, PM, FB, and LSLB verified the data, and all authors approved the final version of the manuscript for publication.

Results

Age-Adjusted Mortality and Vaccine Coverage in the First Year of the Vaccine Roll-Out

From February 26, 2020 to December 31, 2021, 565,774 COVID-19 deaths were reported (**figure 1-A**). Most of these comprised those aged 70 years or older (253,092, 45%), and one-third were those aged under 60 years (**figure 1-B**). The monthly average mortality rates per 100,000 population was the highest in April 2021 for all age groups and occurred during the dominance of VOC Gamma. When comparing the proportion of reported deaths (**figure 1-C**), the 70+ age group showed a decrease since March 2021, two months after the vaccination roll-out start, being the lowest in May-June 2021, almost 50% lower compared to pre-vaccination period. During this period, the proportion of deaths related to 20-49 and 50-59 increased. However, in late 2021, there was a gradual reduction in the proportion of deaths related to those under 60 years.

With respect to the vaccination roll-out, 266,153,517 vaccine doses for COVID-19 were administered in adults (≥20 years old) in 2021, being 137,644,737 (51.66%) first doses and 128,508,780 (48.34%) second or single doses. At the end of 2021, the national first dose vaccination coverage for adults was 90.7%, being 86.74% for 20-49 years, 94.75% for 50-59 years, 100.78% for 60-69 years, and 99.59% for 70+ years (**table 1**). The progression of vaccination coverage rates was different for each age group due to the campaign strategy (**figure 1-D**). Brazil achieved 75% of vaccination coverage in August 2021, six months after the vaccination roll-out. In the same period, the 70+ group had 98% first-dose coverage, whereas the 20-49 presented 52% (**table 1**). We observed that the oldest group (70+) already had more than 90% vaccination coverage by the end of March 2021. Similar behaviour for the second or single dose was observed (**appendix pp 3-4**).

For peer review only

Table 1. Number of reported deaths and first dose vaccine coverage per month in during the COVID-19 vaccination campaign in Brazil (started in January 17, 2021).

Age group	Baseline*	Jan/21	Feb/21	Mar/21	Apr/21	Mai/21	Jun/21	Jul/21	Aug/21	Sep/21	Oct/21	Nov/21	Dec/21
Monthly reported deaths													
BR	123,763	29,346	28,141	62,603	79,939	53,772	48,416	31,293	18,485	12,164	8,254	4,958	3,223
20 – 49	12,298 (10 %)	2,842 (10 %)	3,149 (11 %)	9,099 (15 %)	13,299 (17 %)	10,617 (20 %)	12,620 (26 %)	8,316 (27 %)	3,714 (20 %)	1,626 (13 %)	832 (10 %)	506 (10 %)	372 (12 %)
50 – 59	15,269 (12 %)	3,641 (12 %)	3,748 (13 %)	9,611 (15 %)	14,224 (18 %)	11,623 (22 %)	13,082 (27 %)	7,677 (25 %)	3,022 (16 %)	1,449 (12 %)	830 (10 %)	531 (11 %)	374 (12 %)
60 – 69	28,385 (23 %)	6,845 (23 %)	6,588 (23 %)	15,385 (25 %)	21,775 (27 %)	15,319 (28 %)	9,363 (19 %)	5,210 (17 %)	3,471 (19 %)	2,276 (19 %)	1,634 (20 %)	1,063 (21 %)	770 (24 %)
70+	67,811 (55 %)	16,018 (55 %)	14,656 (52 %)	28,508 (46 %)	30,641 (38 %)	16,213 (30 %)	13,351 (28 %)	10,090 (32 %)	8,278 (45 %)	6,813 (56 %)	4,958 (60 %)	2,858 (58 %)	1,707 (53 %)
Monthly average mortality rates per 100,000 people													
BR	0.44	0.62	0.66	1.33	1.76	1.14	1.06	0.67	0.39	0.27	0.18	0.11	0.07
20 – 49	0.07	0.09	0.12	0.30	0.45	0.35	0.43	0.27	0.12	0.06	0.03	0.02	0.01
50 – 59	0.35	0.49	0.56	1.30	1.99	1.57	1.83	1.04	0.41	0.20	0.11	0.07	0.05
60 – 69	0.92	1.32	1.41	2.97	4.34	2.95	1.87	1.00	0.67	0.45	0.32	0.21	0.15
70+	2.73	3.84	3.89	6.83	7.59	3.88	3.31	2.42	1.98	1.69	1.19	0.71	0.41
Vaccine coverage# (%)													
BR		1.82	4.74	13.75	21.92	32.22	50.82	67.72	84.82	88.32	89.58	90.3	90.67
20 – 49		1.99	3.61	4.56	5.98	12.55	28.85	52.75	78.55	83.55	85.26	86.23	86.72
50 – 59		1.65	3.06	4.08	7.64	31.84	81.34	89.73	92.36	93.57	94.16	94.53	94.75
60 – 69		1.05	2.28	19.58	75.48	95.28	97.61	98.79	99.6	100.09	100.4	100.6	100.72
70+		1.85	18.95	90.35	96.32	97.4	97.99	98.38	98.72	99.07	99.4	99.53	99.61

*Baseline period corresponds to the six-month period (July to December, 2020) previous to the vaccination campaign start.
#At least one dose.

Vaccination Effect on Mortality Rates

To assess the impact of the vaccination campaign strategy on reducing mortality rates, we compared the mortality rates for each age group with the national age-adjusted mortality rates in the country (**figure 2-A**). The 70+ group showed the highest mortality rates in the whole period (Rate ratio > 1). However, six months later after the vaccination roll-out start, the mortality rates reduced from 3.84 deaths per 100,000 population (Rate Ratio: 6.16) in January 2021 to 3.31 deaths per 100,000 (Rate Ratio: 3.11) in June 2021 (**appendix p 5**). In the same period, the 20-49 showed an increase in mortality rates (Rate ratio: 0.21 in January 2021 and 0.58 in June 2021) compared to the national age-adjusted mortality rates. After September 2021, rate ratios for all age groups returned to similar levels to those seen before vaccination (**appendix p 5**).

We estimate the intervention effect using a Negative Binomial Regression model to evaluate the association between the reported deaths, the intervention (age)/ control group, and the intervention period indicator (**figure 2-B**). When analysing the temporal progression of the RRR stratified by age group (**appendix p 6**), the 70+ age group had the lowest rates in June 2021 (RRR [95% CI]: 0.48 [0.43-0.53]), which evidenced a 52% decrease in the number of deaths in 5 months. With regard to age groups 50-59 and 60-69, we estimated a decrease in death rates of 15% and 30% (RRR [95% CI]: 0.85 [0.75-0.96] and RRR [95% CI]: 0.70 [0.63-0.71], respectively), for at least 4 and 6 months in the first year of vaccination (**appendix p 6**).

Number of Prevented and Preventable Deaths and Potential Years of Life Lost (PYLL)

We quantified the number of prevented and preventable deaths in each age group during the vaccination roll-out (**figure 3**). We estimated that vaccination prevented 59,618 deaths compared to the expected deaths in the first year of vaccination roll-out (**table 2**). Of these, 89% was prevented in those aged 70 years or more (53,088), followed by those aged 60-69 years (6,032, 10%), which achieved high vaccination coverage earlier than younger age groups (**figure 1-D**).

For peer review only

Table 2. Comparison between observed and predicted deaths during the vaccination campaign in Brazil, in 2021, stratified by age group.

Results	Age group				Total
	20-49	50-59	60-69	70+	
Observeddeaths	65,582	67,991	86,126	145,812	365,511
Expecteddeaths	39,393	48,098	85,064	197,777	370,332
Preventeddeaths	155	343	6,032	53,088	59,618
Preventabledeaths	26,344	20,236	7,094	1,123	54,797
PreventedPYLL	6,355	7,203	66,352	291,984	371,894
PreventablePYLL	1,080,104	424,956	78,034	6,177	1,589,271

Prevented deaths were obtained if observed deaths was lower than predicted, and preventable deaths, if the number of observed deaths was higher than predicted.
†The denominator is the adult population of Brazil for this age group = 151,778,738.
# PYLL was calculated using the average age for each group and a life expectancy of 76 years for Brazil in 2020.

We considered preventable deaths when the number of observed deaths was higher than those expected if each age group followed the progression of the national age-adjusted mortality rates in the country (**figure 3, table 2**). Despite the considerable number of deaths prevented, we estimated 54,797 potentially preventable deaths in the same period. More than 85% of preventable deaths comprised those under 60 years old, half of them corresponding to those aged 20-49 years (26,344, 48%), which achieved late vaccination coverage compared to older groups (**figure 1-D**).

In addition, we estimated the impact of the vaccination roll-out in terms of the Potential Years of Life Lost (PYLL). The vaccination strategy prevented 371,894 PYLL, especially for those aged 60 years or older. However, it was not able to prevent 1,589,271 PYLL, composed mainly by those aged 20-49 years (68%) (**table 2**).

Discussion

We evaluated the impact of the COVID-19 vaccination strategy in Brazil on the number of reported deaths during the campaign's first year by analysing the progression of vaccination coverage and reported deaths and modelling the prevented and preventable deaths. In prioritising the vaccination of high-risk groups, the mortality rates of the elderly population reduced earlier compared to the young population, especially in a period of high burden by the introduction of a new VOCs. After 6 months of vaccination roll-out, the vaccination coverage for those aged 70+ years was 98% compared to only 52% for the age group 20-49, which resulted in a reduction of 44% the mortality rates in the 70+ years and for those aged 20-49 were still

For peer review only

increasing due to the low coverage. At the end of 2021, the first dose coverage achieved 91% and the estimated number of prevented deaths 59,618. However, the delay in the vaccination of younger ages may have resulted in an expressive number of deaths that could be prevented in this group.

Brazil was one of the epicentres of COVID-19 deaths in 2020, which was intensified in a second and larger surge at the beginning of 2021 when the COVID-19 vaccination campaign started.[3] The first months comprised of limited vaccine doses availability until May 2021. The strategy prioritised the immunisation of high-risk groups (i.e., indigenous, healthcare workers, and comorbidities) and older ages, intending to reduce the burden of deaths and overload over the health system. This strategy resulted in a rapid decrease in mortality rates in the country, driven mainly by those aged 70+, which displayed the highest number of prevented deaths (53,088) in the period. The strategy successfully prevented deaths in the older population; however, the major drawback was the expressive number of deaths in those aged < 60 years old.

Except for those with high-risk conditions, the vaccination for younger groups started only four to six months after the older groups, during the third surge of deaths (July 2021). Although mortality risks for this population are lower compared to the elderly, Brazil is composed mainly of young people. At the end of 2021, 86,834 deaths were reported for the 20-49 group, where 66,692 (77%) occurred during the vaccination roll-out period. The trend of mortality for the youngers started to decrease in July 2021 being more expressive in September 2021, when vaccination coverage of this population was higher than 75%.

We estimated that the vaccination strategy prevented 59,618 deaths, composed mainly by those aged 60-69 or 70+ years. However, 54,797 preventable deaths of those aged 50-59 or 20-49 years occurred, corresponding to potential save of 1,589,271 PYLL, 68% from 20-49 years. These results align with previous studies that identified a reduction in deaths and hospitalizations due to vaccination in high-income countries.[8–11] A recently Brazilian study obtained 303,129 prevented deaths in the period of January 17, 2021 to January 31, 2022, with 61% from those aged >= 60 and 7% for 20-39 age group.[19] Our estimates were obtained using a counterfactual analysis in which the control group represented the progression of national age-adjusted mortality rates during the vaccination campaign. Although it does not represent a scenario strictly without vaccination, we could obtain the number of expected deaths considering the dynamics in pandemic progression and the vaccination roll-out as the speed and effectiveness in the decrease of deaths for each age group. Hence, we were able to evaluate the

For peer review only

results from the age-based calendar strategy and quantify the number of prevented and preventable deaths for each age group during the first year of the vaccination campaign.

Latin America was highly affected by the pandemic, the continent with the highest number of deaths per capita, with limited access to vaccine doses.[20,21] In Brazil, the availability of vaccine doses at the beginning of the vaccination roll-out was low due to the national government's late negotiation for vaccine purchases, global demand, and the high concentration of doses in rich countries.[13,22] The concern with respect to vaccine hesitancy, encouraged by the country's leadership and polarized political environment, was not confirmed by previous evidences and a high vaccination coverage was achieved.[13,22–24] The urgency of vaccination also refers to better use of a common resource, including access to vaccines, prioritizing care for those most affected, i.e., where the epidemic is behaving most intensely.[25]

Recently, the WHO revised the initial recommendation of prioritising the years of life saved to reduce deaths and disease burden, especially in scenarios of limited access to vaccines.[25] In Brazil, due to the low availability of vaccine doses at the start of the roll-out, a strategy that followed an age-based calendar also prioritising high-risk groups was adopted. Our results demonstrated that the strategy protected the oldest. However, it could not significantly prevent the deaths of the youngest. These findings raise concerns about whether the age-based calendar is the best strategy for countries with low-resource settings and a young population[26]. Should alternative strategies prioritise locals with high burden and increase the years of life saved in locations with limited healthcare resources and socioeconomic vulnerability? Alternative strategies to the age-based calendar have been proposed pointing out the importance of considering the burden, age distribution, vaccination supply and speed, hesitancy, and socioeconomic vulnerability to promote a better and more equitable decision-making process.[25,27–30] Other modelling approaches, such as agent-based or deterministic compartmental models, may also assess the potential gains of alternative strategies.[30,31]

The findings of this study should be interpreted in light of some potential limitations. First, we analysed data notified in the SIVEP-Gripe, which consists of cases reported by the surveillance system. This limits the assessment of all COVID-19 deaths and excess mortality in the country. However, the SIVEP-Gripe has been one of the country's central repositories of COVID-19 epidemiological data, with high information coverage[2]. Second, stringency policies and actions, as well as personal behaviour risk, may affect the progression of deaths and mortality rates and were not considered in the analysis. Furthermore, the study has strengths: this is a nationwide data analysis of a large LMIC with a high burden of COVID-19; we estimate

For peer review only

the dynamic impact of the vaccination program in four age groups considering an average behaviour, which allows the assessment and understanding of the vaccination roll-out within the country.

Conclusion

Our findings indicate that the vaccination campaign was able to prevent more than 59,000 deaths in Brazil, especially for the older population. However, the delay in the vaccination of younger ages may have resulted in an expressive number of deaths that could be prevented in this group.

For peer review only

Data availability statement

Data are available on reasonable request.

Ethics statements

Patient consent for publication

Not applicable.

Acknowledgements

This work is part of the Grand Challenges ICODA pilot initiative, delivered by Health Data Research UK and funded by the Bill & Melinda Gates Foundation and the Minderoo Foundation. This study was also supported by the National Council for Scientific and Technological Development (CNPq), the Coordination for the Improvement of Higher Education Personnel (CAPES) - Finance Code 001, Carlos Chagas Filho Foundation for Research Support of the State of Rio de Janeiro (FAPERJ), the Pontifical Catholic University of Rio de Janeiro. OTR is funded by a Sara Borrell grant from the Instituto de Salud Carlos III (CD19/00110). OTR acknowledges support from the Spanish Ministry of Science and Innovation and State Research Agency through the "Centro de Excelencia Severo Ochoa 2019-2023" Program (CEX2018-000806-S), and support from the Generalitat de Catalunya through the CERCA Program.

We are grateful to all healthcare workers for their endless efforts to tackle COVID-19 pandemic in Brazil. We thank the NOIS research group and the ICODA driver project EFFECT-Brazil team members for the discussions and collaborative production during the COVID-19 pandemic. All authors carried out the research independently of the funding body. The findings and conclusions in this manuscript reflect the opinions of the authors alone.

For peer review only

Contributors

SA, LSLB, PM, FB, PS, JCN, OTR, SH and FAB participated in the design and concept of the study. SA, LSLB and PM did the data curation. SA, LSBL, PM, FB, OTR and FAB designed the data analysis. SA, LSLB, PM, FB, PS, SH and FAB performed the data analysis. SA, LSLB, PM, FB and FAB wrote the first version of the manuscript. LSLB, PM, FB, SH and FAB supervised the study. All authors had full access to data, participated in data interpretation, revised the manuscript, and approved the final version of the manuscript.

Funding

This work is part of the Grand Challenges ICODA pilot initiative, delivered by Health Data Research UK and funded by the Bill & Melinda Gates Foundation (INV-017293) and the Minderoo Foundation. This study was supported by the National Council for Scientific and Technological Development (CNPq), the Coordination for the Improvement of Higher Education Personnel (CAPES) - Finance Code 001, Carlos Chagas Filho Foundation for Research Support of the State of Rio de Janeiro (FAPERJ) and the Pontifical Catholic University of Rio de Janeiro. OTR is funded by a Sara Borrell grant from the Instituto de Salud Carlos III (CD19/00110). OTR acknowledges support from the Spanish Ministry of Science and Innovation and State Research Agency through the "Centro de Excelencia Severo Ochoa 2019-2023" Program (CEX2018-000806-S), and support from the Generalitat de Catalunya through the CERCA Program.

All authors carried out the research independently of the funding bodies. The findings and conclusions in this manuscript reflect the opinions of the authors alone.

For peer review only

Competing interests

SH and FAB are funded by the CNPq and FAPERJ. PM is funded by CNPq (422470/2021-0) and FAPERJ (E-26/211.645/2021 and E-26/201.348/2022). OTR is funded by a Sara Borrell fellowship from the Instituto de Salud Carlos III (CD19/00110), acknowledges support from the Spanish Ministry of Science and Innovation and State Research Agency through the “Centro de Excelencia Severo Ochoa 2019–2023” Program (CEX2018-000806-S), support from the Generalitat de Catalunya through the CERCA Program, and received a research grant from the Health Effects Institute for research unrelated to this manuscript. OTR was also member of the Data Safety Monitoring Board in the REVOLUTION and STOP-COVID trials, testing treatments

against COVID-19, and is currently member of the Data Safety Monitoring Board of the RENOVATE trial, testing respiratory support strategies in patients with acute respiratory hypoxaemic failure. The other authors declare that they have no conflict of interest.

All authors carried out the research independently of the funding bodies. The findings and conclusions in this manuscript reflect the opinions of the authors alone.

Data sharing

All data, including individual participant data, is publicly available with de-identification and anonymisation of patients. The data sources are described in the manuscript and in the supplementary material (**appendix p 2**). The raw data and code used for the analysis are available in a GitHub repository, with publication (https://github.com/noispuc/ICODA_COVID_VaccineStrategy)

For peer review only

References

1 Castro MC, Kim S, Barberia L, *et al.* Spatiotemporal pattern of COVID-19 spread in Brazil. *Science (80-)* 2021; **372**: 821–6.

2 Ranzani OT, Bastos LSL, Gelli JGM, *et al.* Characterisation of the first 250 000 hospital admissions for COVID-19 in Brazil: a retrospective analysis of nationwide data. *Lancet Respir Med* 2021; **9**: 407–18.

3 Bastos LS, Ranzani OT, Souza TML, Hamacher S, Bozza FA. COVID-19 hospital admissions: Brazil's first and second waves compared. *Lancet Respir Med* 2021; **9**: e82–3.

4 Faria NR, Mellan TA, Whittaker C, *et al.* Genomics and epidemiology of the P.1 SARS-CoV-2 lineage in Manaus, Brazil. *Science (80-)* 2021; **372**: 815–21.

5 Sabino EC, Buss LF, Carvalho MPS, *et al.* Resurgence of COVID-19 in Manaus, Brazil, despite high seroprevalence. *Lancet* 2021; **397**: 452–5.

6 Planas D, Saunders N, Maes P, *et al.* Considerable escape of SARS-CoV-2 Omicron to antibody neutralization. *Nature* 2022; **602**: 671–5.

7 Voigt A, Omholt S, Almaas E. Comparing the impact of vaccination strategies on the spread of COVID-19, including a novel household-targeted vaccination strategy. *PLoS One* 2022; **17**: 1–16.

8 Kim D, Lee YJ. Vaccination strategies and transmission of COVID-19: Evidence across advanced countries. *J Health Econ* 2022; **82**: 102589.

9 Shoukat A, Vilches TN, Moghadas SM, *et al.* Lives saved and hospitalizations averted by COVID-19 vaccination in New York City: a modeling study. *Lancet Reg Heal - Am* 2022; **5**: 100085.

10 Steele MK, Couture A, Reed C, *et al.* Estimated Number of COVID-19 Infections, Hospitalizations, and Deaths Prevented among Vaccinated Persons in the US, December 2020 to September 2021. *JAMA Netw Open* 2022; **5**: E2220385.

11 Ferreira LS, Marquitti FMD, Silva RLP da, *et al.* Estimating the impact of implementation and timing of COVID-19 vaccination program in Brazil: a counterfactual analysis. *medRxiv* 2021; : 2021.12.24.21268384.

12 Peres KC, Buendgens FB, Prates EA, *et al.* Vacinas no Brasil: análise histórica do registro sanitário e a disponibilização no Sistema de Saúde. *Cien Saude Colet* 2021; **26**: 5509–22.

13 Bastos LS, Aguilar S, Rache B, *et al.* Primary healthcare protects vulnerable populations from inequity in COVID-19 vaccination: An ecological analysis of nationwide data from Brazil. *Lancet Reg Heal* 2022; : 100335.

14 Karaivanov A, Kim D, Lu SE, Shigeoka H. COVID-19 vaccination mandates and vaccine uptake. *Nat Hum Behav* 2022; : 1–10.

15 Fredriksson A, Oliveira GM de. Impact evaluation using Difference-in-Differences. *RAUSP Manag J* 2019; **54**: 519–32.

16 Matthay EC, Farkas K, Goin DE, Rudolph KE, Pear VA, Ahern J. Associations of firearm dealer openings with firearm self-harm deaths and injuries: A differences-in-differences

For peer review only

analysis. *PLoS One* 2021; **16**: 1–11.

17 Izbicki R, Bastos LS, Izbicki M, Lopes HF, Dos Santos TM. How many hospitalizations has the covid-19 vaccination already prevented in são paulo? *Clinics* 2021; **76**: 3–4.

18 Mitra AK, Payton M, Kabir N, Whitehead A, Ragland KN, Brown A. Potential years of life lost due to COVID-19 in the United States, Italy, and Germany: An old formula with newer ideas. *Int J Environ Res Public Health* 2020; **17**: 1–9.

19 Dos Santos CVB, de Noronha TG, Werneck G, Struchiner CJ, Villela DAM. Estimated COVID-19 Severe Cases and Deaths Averted in the First Year Of the Vaccination Campaign in Brazil: A Retrospective Observational Study. *Lancet Reg Heal* 2023; **17**: 100418.

20 Cifuentes-Faura J. COVID-19 mortality rate and its incidence in Latin America: Dependence on demographic and economic variables. *Int J Environ Res Public Health* 2021; **18**.

21 Hotez PJ, Narayan KMV. Sharing Technology and Vaccine Doses to Address Global Vaccine Inequity and End the COVID-19 Pandemic. *JAMA - J Am Med Assoc* 2021; **326**: 219–20.

22 Fonseca EM da, Shadlen KC, Bastos FI. The politics of COVID-19 vaccination in middle-income countries: Lessons from Brazil. *Soc Sci Med* 2021; **281**: 114093.

23 Gramacho WG, Turgeon M. When politics collides with public health: COVID-19 vaccine country of origin and vaccination acceptance in Brazil. *Vaccine* 2021; **39**: 2608–12.

24 Moore DCBC, Nehab MF, Camacho KG, *et al.* Low COVID-19 vaccine hesitancy in Brazil. *Vaccine* 2021; **39**: 6262–8.

25 Faden R, Cravioto A, Hombach J, *et al.* Who to vaccinate first? A peek at decision-making in a pandemic. *Nature* 2022; **607**: 235–8.

26 Watson OJ, Barnsley G, Toor J, Hogan AB, Winskill P, Ghani AC. Global impact of the first year of COVID-19 vaccination: a mathematical modelling study. *Lancet Infect Dis* 2022; **22**: 1293–302.

27 Hogan AB, Winskill P, Watson OJ, *et al.* Within-country age-based prioritisation, global allocation, and public health impact of a vaccine against SARS-CoV-2: A mathematical modelling analysis. *Vaccine* 2021; **39**: 2995–3006.

28 McIntyre PB, Aggarwal R, Jani I, *et al.* COVID-19 vaccine strategies must focus on severe disease and global equity. *Lancet* 2022; **399**: 406–10.

29 Sachs JD, Karim SSA, Aknin L, *et al.* The Lancet Commission on lessons for the future from the COVID-19 pandemic. *Lancet* 2022; **400**.

30 Saadi N, Chi YL, Ghosh S, *et al.* Models of COVID-19 vaccine prioritisation: a systematic literature search and narrative review. *BMC Med* 2021; **19**: 1–11.

31 Liu Y, Sandmann FG, Barnard RC, *et al.* Optimising health and economic impacts of COVID-19 vaccine prioritisation strategies in the WHO European Region: a mathematical modelling study. *Lancet Reg Heal - Eur* 2022; **12**: 100267.

For peer review only

Figure legends

Figure 1. Progression of COVID-19 reported deaths and the vaccination roll-out in Brazil: (A) Daily number of reported deaths (15-day moving average, left y-axis) and first dose vaccine coverage (right y-axis) from February 26, 2020 to December 31, 2021; yellow shaded areas display the period of VOC dominance (50% of samples using oubreak.info/GISAID data) for Gamma (February 15, 2021) and Delta (August 11, 2021) - vertical dashed lines indicate the start of the vaccination roll-out in Brazil and the 75% first vaccination coverage of adult population, respectively; (B) Daily number reported deaths stratified by age group in 2021; (C) Daily proportions of deaths reported for each age group (number of deaths / total number of deaths per day); (D) First dose vaccination coverage stratified by age group - vertical dashed lines refer to the start of the vaccination campaign for each age group, the horizontal dashed line indicates the 75% vaccination coverage target by the World Health Organization (WHO).

Figure 2. The impact of the COVID-19 vaccination campaign in reported deaths stratified by age groups: (A) Rate ratios comparing daily mortality rates stratified by age group with the national mortality rate; (B) Estimated effect of the vaccination campaign in each age groups from the difference-in-differences (DID) analysis. Effects were obtained as the Ratio of Rate Ratios (RRR) and their respective 95% confidence intervals. The vertical dashed line refers to the start of the vaccination campaign on January 17, 2021. DID analysis considered six months before the vaccination roll-out as the baseline period and the national mortality rates as the control group in a Negative Binomial Regression model.

Figure 3. Estimated prevented and preventable deaths stratified by age group. For each age group (coloured lines), the predicted deaths (black lines) were obtained whether that age-group followed the national age-adjusted mortality rates. Grey lines correspond to the fitted linear model. Predicted deaths were estimated from the period when 10% of first dose vaccination coverage was achieved for each age group (black dashed line). Prevented deaths (green-shaded area) were obtained if observed deaths was lower than predicted, and preventable deaths (red-shaded area), if the number of observed deaths was higher than predicted. Left-first vertical dashed lines refer to the start of the vaccination campaign in January 17, 2021.

For peer review only

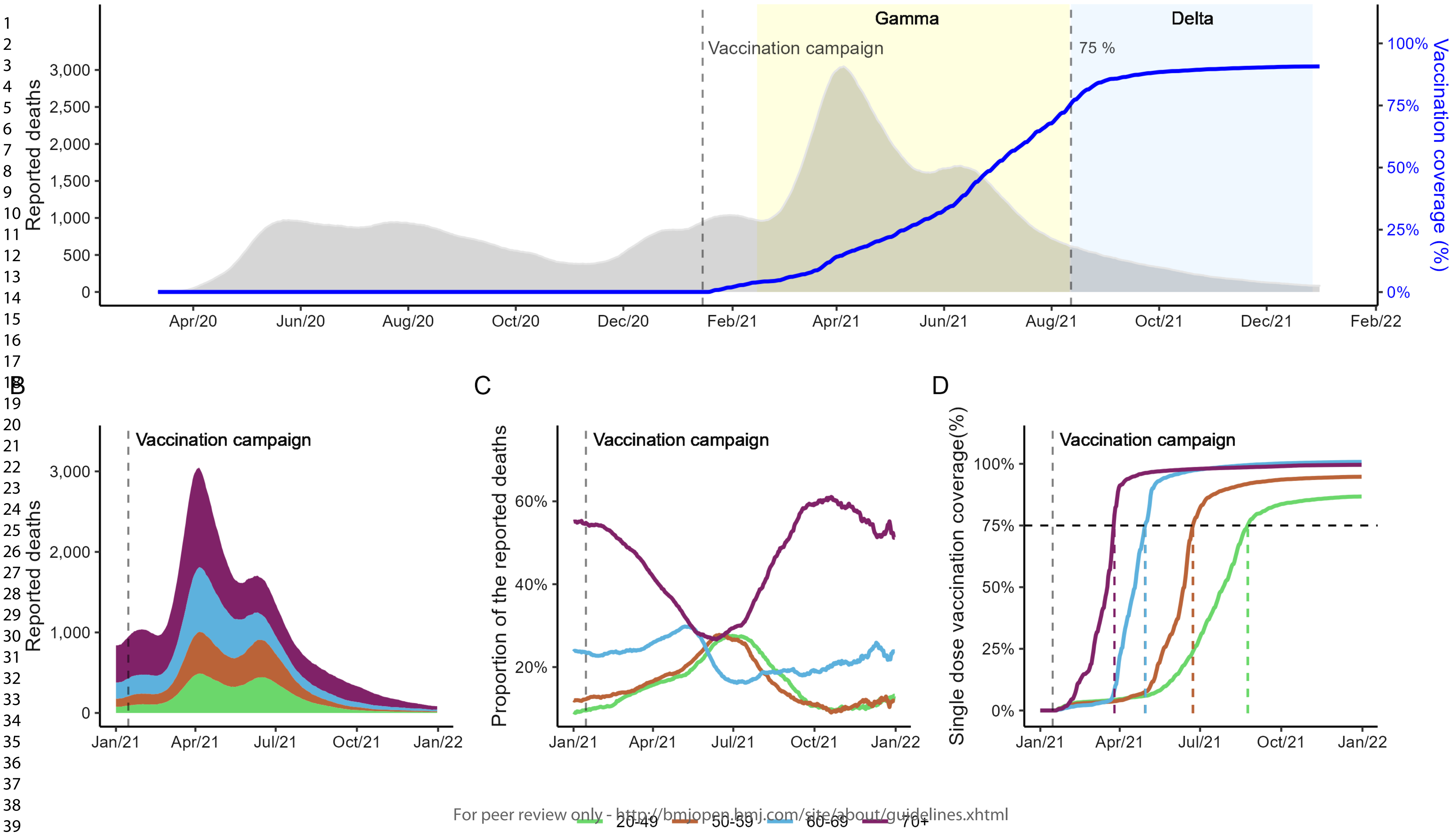
A
Gamma
Delta
Vaccination campaign
75 %
Reported deaths
3,000
2,500
2,000
1,500
1,000
500
0
100%
75%
50%
25%
0%
Vaccination coverage (%)
Apr/20
Jun/20
Aug/20
Oct/20
Dec/20
Feb/21
Apr/21
Jun/21
Aug/21
Oct/21
Dec/21
Feb/22
B
Vaccination campaign
Reported deaths
3,000
2,000
1,000
0
Jan/21
Apr/21
Jul/21
Oct/21
Jan/22
C
Vaccination campaign
Proportion of the reported deaths
60%
40%
20%
Jan/21
Apr/21
Jul/21
Oct/21
Jan/22
D
Vaccination campaign
Single dose vaccination coverage(%)
100%
75%
50%
25%
0%
Jan/21
Apr/21
Jul/21
Oct/21
Jan/22
20-49
50-59
60-69
70+

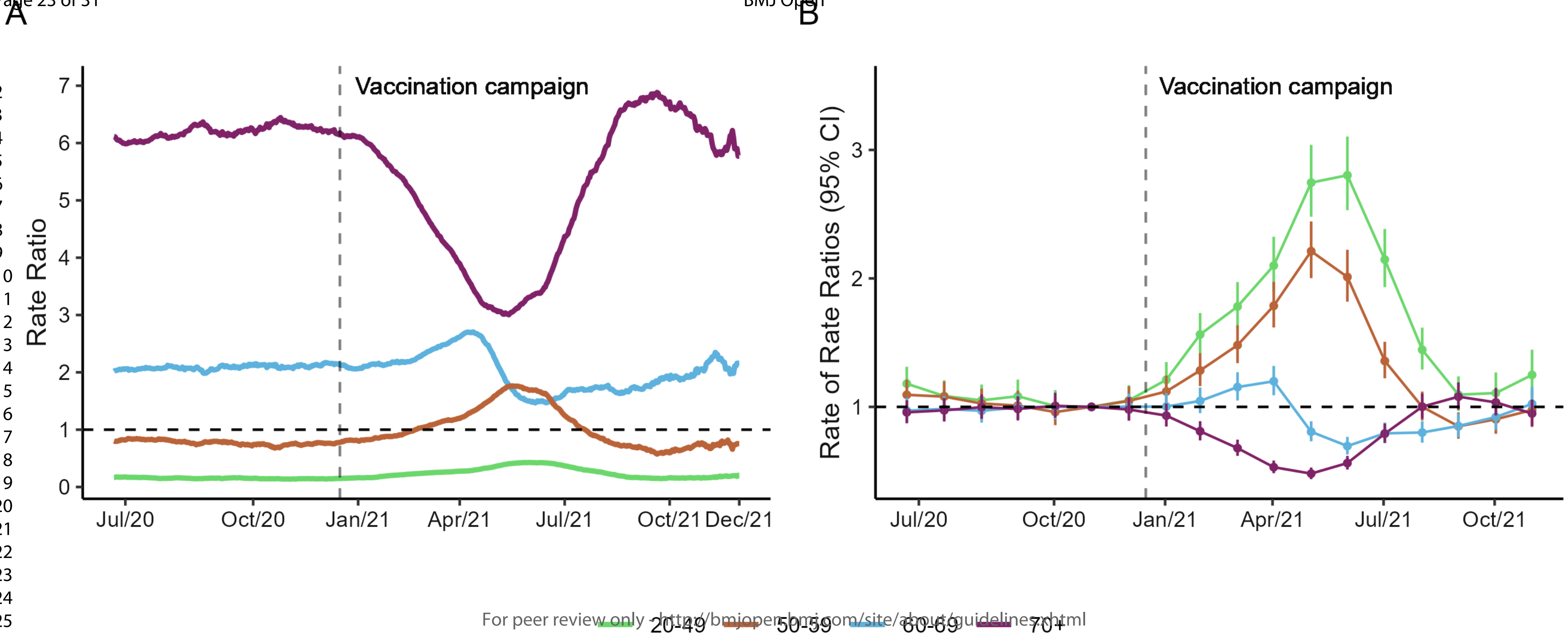
A
B
Vaccination campaign
Vaccination campaign
Rate Ratio
Rate of Rate Ratios (95% CI)
0
1
2
3
4
5
6
7
Jul/20
Oct/20
Jan/21
Apr/21
Jul/21
Oct/21
Dec/21
20-49
50-59
60-69
70+

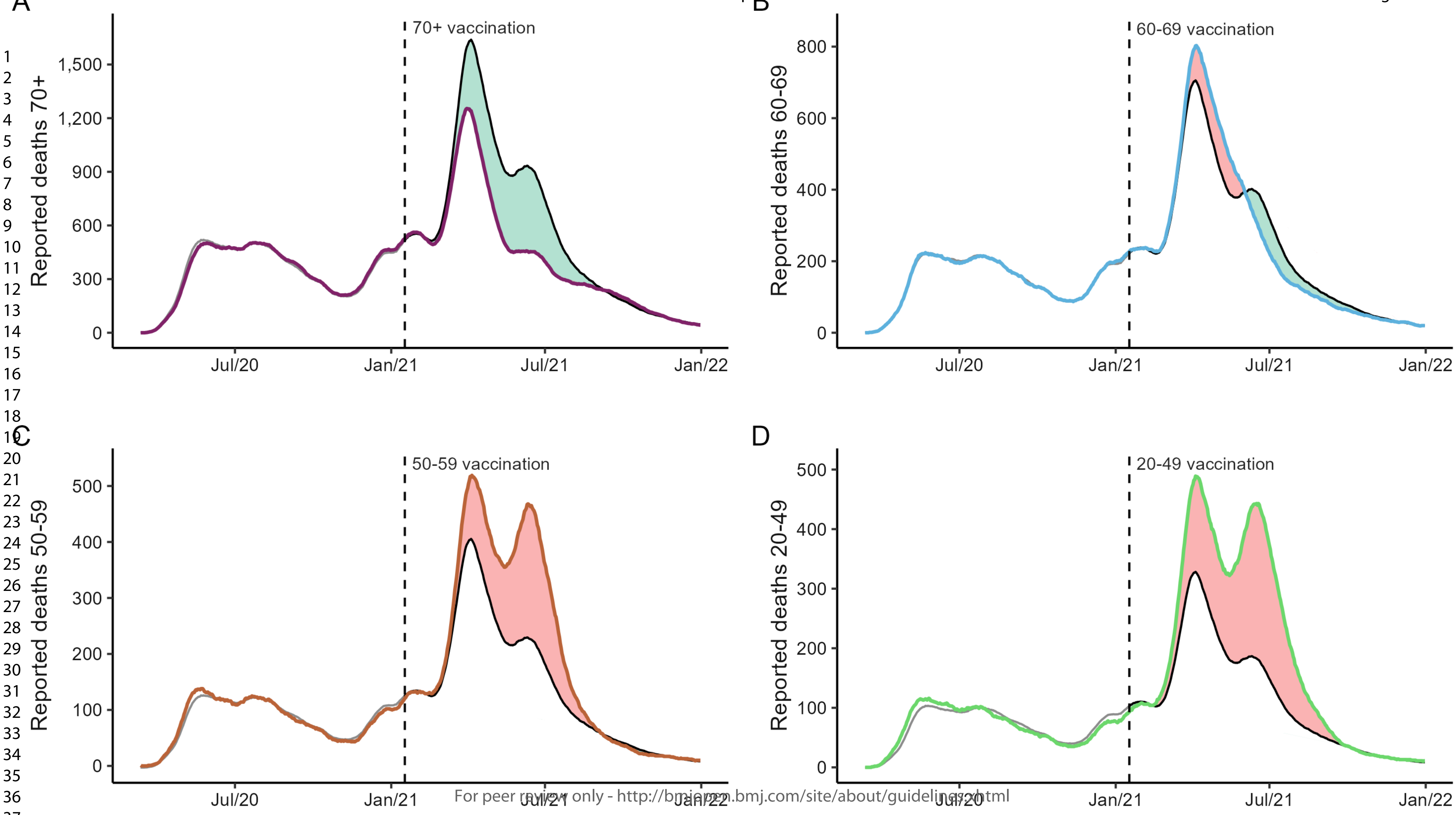
A
70+ vaccination
Reported deaths 70+
0
300
600
900
1,200
1,500
Jul/20
Jan/21
Jul/21
Jan/22
B
60-69 vaccination
Reported deaths 60-69
0
200
400
600
800
Jul/20
Jan/21
Jul/21
Jan/22
C
50-59 vaccination
Reported deaths 50-59
0
100
200
300
400
500
Jul/20
Jan/21
Jul/21
Jan/22
D
20-49 vaccination
Reported deaths 20-49
0
100
200
300
400
500
Jul/20
Jan/21
Jul/21
Jan/22

Supplementary Material

Table of contents

Figure S1. (A) First dose vaccination coverage stratified by age group – first vertical dashed line on the left refers to the start of the vaccination campaign for each age group, the horizontal dashed line indicates the 75% vaccination coverage target by the World Health Organization (WHO). (B) Second or single vaccination coverage stratified by age group. 3
Table S1. Description of the data sources used in this study 2
Table S2. Second or single vaccination coverage during the vaccination roll-out in Brazil, in 2021, stratified by age group. 4
Table S3. Monthly average of the mortality ratios in Brazil, in 2021, stratified by age group. 5
Table S4. Monthly and global estimated effect of the vaccination campaign (DID analysis) with their respective 95% confidence intervals stratified by age group. 6

For peer review only - http://bmjopen.bmj.com/site/about/guidelines.xhtml

Table S1. Description of the data sources used in this study

Data	Source	Source address	Version	Date exported
COVID-19 Vaccination doses	National Immunisation Program Information System,"SI-PNI"	*OpenDataSUS* repository: https://opendatasus.saude.gov.br/dataset/covid-19-vacinacao	21/07/2022	21/07/2022
Brazilian population estimates (per age, sex, and municipalities)	Health Surveillance Secretary from the Brazilian Ministry of Health	Official website: http://tabnet.datasus.gov.br/cgi/tabcgi.exe?popsvs/cnv/popbr.def	2020	07/02/2022
COVID-19 hospitalized cases	Influenza Epidemiological Surveillance Information System, "SIVEP-Gripe"	*OpenDataSUS* repository: https://opendatasus.saude.gov.br/dataset/bd-srag-2020 https://opendatasus.saude.gov.br/dataset/srag-2021-e-2022	21/07/2022	21/07/2022
Life expectancy at birth, total (years) - Brazil	World Bank	Official website: https://data.worldbank.org/indicator/SP.DYN.LE00.IN?locations=BR&most_recent_year_desc=true	2019	25/07/2022

COVID-19: Disease caused by the SARS-CoV-2 virus.

For peer review only

For peer review only - http://bmjopen.bmj.com/site/about/guidelines.xhtml

Figure S1. (A) First dose vaccination coverage stratified by age group – first vertical dashed line on the left refers to the start of the vaccination campaign for each age group, the horizontal dashed line indicates the 75% vaccination coverage target by the World Health Organization (WHO). (B) Second or single vaccination coverage stratified by age group.

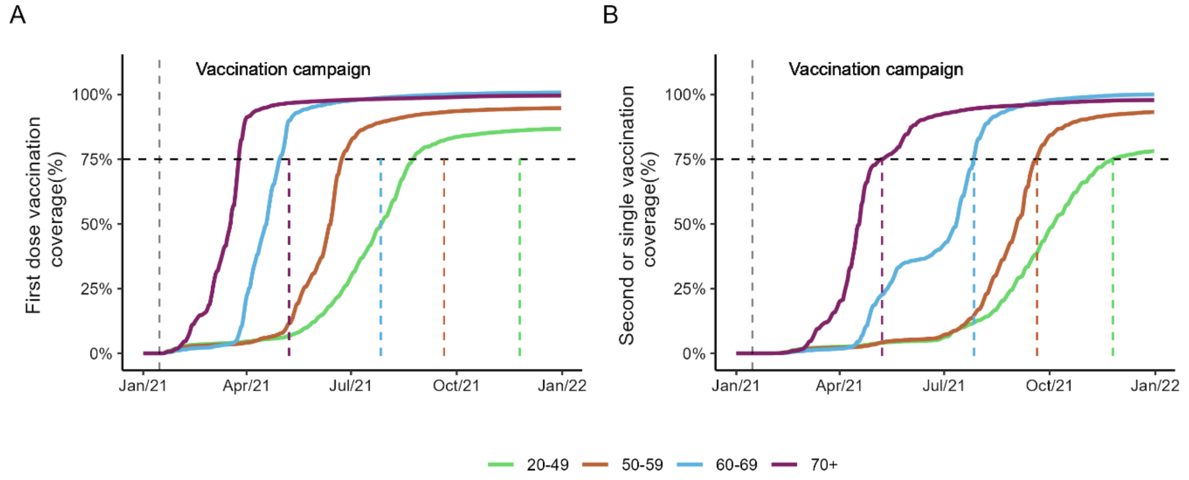

For peer review only

Table S2. Second or single vaccination coverage during the vaccination roll-out in Brazil, in 2021, stratified by age group.

Age group	Jan/21	Feb/21	Mar/21	Apr/21	Mai/21	Jun/21	Jul/21	Aug/21	Sep/21	Oct/21	Nov/21	Dec/21
Vaccine coverage (%)												
BR	0	1.56	3.86	11.37	15.26	17.78	28.38	42.88	62.88	76.18	82.88	84.55
20 – 49	0	1.7	2.54	3.7	4.65	6.05	12.74	26.04	47.34	66.04	75.76	78.08
50 – 59	0	1.45	2.19	3.4	5.21	6.95	17.05	46.05	83.75	90.42	92.44	93.29
60 – 69	0	0.87	1.79	18.39	35.29	41.64	83.04	94.74	97.78	98.94	99.71	100
70+	0.01	1.67	19.17	72.57	85.27	92.52	94.75	95.68	96.59	97.27	97.69	97.83

For peer review only

For peer review only - http://bmjopen.bmj.com/site/about/guidelines.xhtml

Table S3. Monthly average of the mortality ratios in Brazil, in 2021, stratified by age group.

Age group	Jan/21	Feb/21	Mar/21	Apr/21	Mai/21	Jun/21	Jul/21	Aug/21	Sep/21	Oct/21	Nov/21	Dec/21
20 – 49	0.15	0.17	0.22	0.26	0.31	0.41	0.41	0.31	0.2	0.16	0.16	0.18
50 – 59	0.79	0.85	0.96	1.14	1.38	1.72	1.53	1.03	0.75	0.63	0.69	0.74
60 – 69	2.12	2.12	2.22	2.48	2.58	1.74	1.52	1.7	1.7	1.81	1.95	2.16
70+	6.16	5.87	5.21	4.29	3.38	3.11	3.69	5.1	6.35	6.77	6.48	5.96

For peer review only

Table S4. Monthly and global estimated effect of the vaccination campaign (DID analysis) with their respective 95% confidence intervals stratified by age group.

Age group	Global	Jan/21	Feb/21	Mar/21	Apr/21	Mai/21	Jun/21	Jul/21	Aug/21	Sep/21	Oct/21	Nov/21	Dec/21
20 – 49	1.75 (1.44 - 2.12)	1.05 (0.95 - 1.17)	1.21 (1.09 - 1.35)	1.56 (1.41 - 1.73)	1.78 (1.61 - 1.97)	2.10 (1.90 - 2.32)	2.75 (2.48 - 3.04)	2.80 (2.53 - 3.10)	2.15 (1.93 - 2.38)	1.44 (1.29 - 1.62)	1.09 (0.97 - 1.24)	1.11 (0.97 - 1.27)	1.25 (1.08 - 1.44)
50 – 59	1.47 (1.21 - 1.78)	1.05 (0.94 - 1.16)	1.12 (1.01 - 1.24)	1.28 (1.16 - 1.42)	1.48 (1.34 - 1.64)	1.79 (1.62 - 1.97)	2.21 (2.00 - 2.44)	2.01 (1.82 - 2.22)	1.36 (1.22 - 1.51)	1.00 (0.90 - 1.12)	0.85 (0.75 - 0.96)	0.90 (0.79 - 1.03)	0.98 (0.84 - 1.13)
60 – 69	1.01 (0.84 - 1.23)	1.00 (0.91 - 1.10)	1.00 (0.91 - 1.11)	1.05 (0.95 - 1.15)	1.15 (1.05 - 1.23)	1.20 (1.09 - 1.27)	0.81 (0.73 - 1.32)	0.70 (0.63 - 0.71)	0.79 (0.72 - 0.88)	0.80 (0.72 - 0.89)	0.85 (0.76 - 0.95)	0.92 (0.82 - 1.03)	1.02 (0.91 - 1.16)
70+	0.73 (0.60 - 0.88)	0.98 (0.89 - 1.08)	0.93 (0.85 - 1.03)	0.81 (0.74 - 0.89)	0.68 (0.62 - 0.75)	0.53 (0.48 - 0.58)	0.48 (0.43 - 0.53)	0.56 (0.51 - 0.62)	0.79 (0.72 - 0.87)	1.00 (0.91 - 1.10)	1.08 (0.98 - 1.19)	1.04 (0.93 - 1.15)	0.95 (0.85 - 1.06)

For peer review only

For peer review only - http://bmjopen.bmj.com/site/about/guidelines.xhtml

STROBE 2007 (v4) Statement—Checklist of items that should be included in reports of cross-sectional studies.

Section/Topic	Item No	Recommendation	Reported on page #
**Title and abstract**	1	(*a*) Indicate the study's design with a commonly used term in the title or the abstract	Title, Page 1
		(*b*) Provide in the abstract an informative and balanced summary of what was done and what was found	Abstract, Methods/Findings, Page 2
**Introduction**			
Background/rationale	2	Explain the scientific background and rationale for the investigation being reported	Page 4
Objectives	3	State specific objectives, including any prespecified hypotheses	Page 4
**Methods**			
Study design	4	Present key elements of study design early in the paper	Methods, Pages 4-8, Table S1
Setting	5	Describe the setting, locations, and relevant dates, including periods of recruitment, exposure, follow-up, and data collection	Pages 4-8
Participants	6	(*a*) *Cross-sectional study*—Give the eligibility criteria, and the sources and methods of selection of participants	Page 4-5
Variables	7	Clearly define all outcomes, exposures, predictors, potential confounders, and effect modifiers. Give diagnostic criteria, if applicable	Pages 6-7, Figure S1
Data sources/ measurement	8*	For each variable of interest, give sources of data and details of methods of assessment (measurement). Describe comparability of assessment methods if there is more than one group	Methods, Study design and Participants, Pages 5-7, Table S1
Bias	9	Describe any efforts to address potential sources of bias	Methods, Pages 6-7
Study size	10	Explain how the study size was arrived at	Methods, Data analysis, Pages 6-7
Quantitative variables	11	Explain how quantitative variables were handled in the analyses. If applicable, describe which groupings were chosen and why	Methods, Data analysis, Pages 6-7, Table S3
Statistical methods	12	(*a*) Describe all statistical methods, including those used to control for confounding	Methods, Data analysis, Pages 6-7
		(*b*) Describe any methods used to examine subgroups and interactions	Methods, Data analysis, Pages 6-7
		(*c*) Explain how missing data were addressed	Methods, Data analysis, Pages 6
		(*d*) *Cross-sectional study*—If applicable, describe analytical methods taking account of sampling strategy	NA
		(*e*) Describe any sensitivity analyses	Methods, Data analysis, Pages 6-7
**Results**			

For peer review only - http://bmjopen.bmj.com/site/about/guidelines.xhtml

Participants	13*	(a) Report numbers of individuals at each stage of study—eg numbers potentially eligible, examined for eligibility, confirmed eligible, included in the study, completing follow-up, and analysed	Results, Page 8
		(b) Give reasons for non-participation at each stage	NA
		(c) Consider use of a flow diagram	NA
Descriptive data	14*	(a) Give characteristics of study participants (eg demographic, clinical, social) and information on exposures and potential confounders	Results, Page 8, Table 1
		(b) Indicate number of participants with missing data for each variable of interest	Results, Page 8, Table 1
		(c) *Cohort study*—Summarise follow-up time (eg, average and total amount)	NA
Outcome data	15*	*Cross-sectional study*—Report numbers of outcome events or summary measures	Results, Pages 8-9
Main results	16	(*a*) Give unadjusted estimates and, if applicable, confounder-adjusted estimates and their precision (eg, 95% confidence interval). Make clear which confounders were adjusted for and why they were included	Results, Pages 8-11, Figure 2-3, Figure S1, Tables S2-S4
		(*b*) Report category boundaries when continuous variables were categorized	Results, Pages 8-11, Table 1
		(*c*) If relevant, consider translating estimates of relative risk into absolute risk for a meaningful time period	NA
Other analyses	17	Report other analyses done—eg analyses of subgroups and interactions, and sensitivity analyses	Results, Pages 9-11, Figure 2, Figure 3,
**Discussion**			
Key results	18	Summarise key results with reference to study objectives	Discussion, Pages 11-12
Limitations	19	Discuss limitations of the study, taking into account sources of potential bias or imprecision. Discuss both direction and magnitude of any potential bias	Discussion, Pages 13-14
Interpretation	20	Give a cautious overall interpretation of results considering objectives, limitations, multiplicity of analyses, results from similar studies, and other relevant evidence	Discussion, Pages 11-14
Generalisability	21	Discuss the generalisability (external validity) of the study results	Discussion, Pages 12-13
**Other information**			
Funding	22	Give the source of funding and the role of the funders for the present study and, if applicable, for the original study on which the present article is based	Page 16

*Give information separately for cases and controls in case-control studies and, if applicable, for exposed and unexposed groups in cohort and cross-sectional studies.

Note: An Explanation and Elaboration article discusses each checklist item and gives methodological background and published examples of transparent reporting. The STROBE checklist is best used in conjunction with this article (freely available on the Web sites of PLoS Medicine at http://www.plosmedicine.org/, Annals of Internal Medicine at http://www.annals.org/, and Epidemiology at http://www.epidem.com/). Information on the STROBE Initiative is available at www.strobe-statement.org.

For peer review only

BMJ Open

The impact of the first year of COVID-19 vaccination strategy in Brazil: An ecological study

Journal:	*BMJ Open*
Manuscript ID	bmjopen-2023-072314.R1
Article Type:	Original research
Date Submitted by the Author:	30-Aug-2023
Complete List of Authors:	Aguilar, Soraida; Pontifical Catholic University of Rio de Janeiro, Department of Industrial Engineering<br>Bastos, Leonardo S. L.; Pontifical Catholic University of Rio de Janeiro, Department of Industrial Engineering<br>Maçaira, Paula; Pontifical Catholic University of Rio de Janeiro, Department of Industrial Engineering<br>Baião, Fernanda; Pontifical Catholic University of Rio de Janeiro, Department of Industrial Engineering<br>Simões, Paulo; Pontifical Catholic University of Rio de Janeiro, Department of Industrial Engineering<br>Cerbino-Neto, José; Oswaldo Cruz Foundation<br>Ranzani, Otavio; ISGlobal<br>Hamacher, Silvio; Pontifical Catholic University of Rio de Janeiro, Department of Industrial Engineering<br>Bozza, Fernando; D'Or Institute for Research and Education
**Primary Subject Heading**:	Epidemiology
Secondary Subject Heading:	Public health, Infectious diseases, Health policy
Keywords:	COVID-19, Public health < INFECTIOUS DISEASES, EPIDEMIOLOGY

SCHOLARONE™
Manuscripts

I, the Submitting Author has the right to grant and does grant on behalf of all authors of the Work (as defined in the below author licence), an exclusive licence and/or a non-exclusive licence for contributions from authors who are: i) UK Crown employees; ii) where BMJ has agreed a CC-BY licence shall apply, and/or iii) in accordance with the terms applicable for US Federal Government officers or employees acting as part of their official duties; on a worldwide, perpetual, irrevocable, royalty-free basis to BMJ Publishing Group Ltd ("BMJ") its licensees and where the relevant Journal is co-owned by BMJ to the co-owners of the Journal, to publish the Work in this journal and any other BMJ products and to exploit all rights, as set out in our licence.

The Submitting Author accepts and understands that any supply made under these terms is made by BMJ to the Submitting Author unless you are acting as an employee on behalf of your employer or a postgraduate student of an affiliated institution which is paying any applicable article publishing charge ("APC") for Open Access articles. Where the Submitting Author wishes to make the Work available on an Open Access basis (and intends to pay the relevant APC), the terms of reuse of such Open Access shall be governed by a Creative Commons licence – details of these licences and which Creative Commons licence will apply to this Work are set out in our licence referred to above.

Other than as permitted in any relevant BMJ Author's Self Archiving Policies, I confirm this Work has not been accepted for publication elsewhere, is not being considered for publication elsewhere and does not duplicate material already published. I confirm all authors consent to publication of this Work and authorise the granting of this licence.

For peer review only

The impact of the first year of COVID-19 vaccination strategy in Brazil: An ecological study

Soraida Aguilar, PhD[1]; Leonardo S. L. Bastos, PhD[1,2]; Paula Maçaira, PhD[1]; Fernanda Baião, PhD[1]; Paulo Simões, MSc[1]; José Cerbino-Neto[3], MD, PhD; Otavio T. Ranzani, MD, PhD[4,5]; Silvio Hamacher, PhD[1,2]; Fernando A. Bozza, MD, PhD[3,6,*]

1. Department of Industrial Engineering (DEI), Pontifical Catholic University of Rio de Janeiro (PUC-Rio), Rio de Janeiro, RJ, 22451-900, Brazil

2. Tecgraf Institute, Pontifical Catholic University of Rio de Janeiro (PUC-Rio), Rio de Janeiro, RJ, 22451-900, Brazil

3. National Institute of Infectious Disease Evandro Chagas (INI), Oswaldo Cruz Foundation (FIOCRUZ), Rio de Janeiro, RJ, 21045-900, Brazil

4. Barcelona Institute for Global Health, ISGlobal, Barcelona, 08003, Spain.

5. Pulmonary Division, Heart Institute (InCor), Hospital das Clinicas HCFMUSP, Faculdade de Medicina da Universidade de São Paulo, São Paulo, Brazil

6. D'Or Institute for Research and Education (IDOR), Rio de Janeiro, RJ, 22281-100, Brazil

Corresponding author:

*Fernando A. Bozza, MD, PhD**.,** Laboratório de Pesquisa Clínica em Medicina Intensiva, Instituto Nacional de Infectologia Evandro Chagas (INI), Fundação Oswaldo Cruz (FIOCRUZ), Av. Brasil 4365, Rio De Janeiro, RJ, 21045-900, Brazil.

Email: fernando.bozza@ini.fiocruz.br or bozza.fernando@gmail.com

For peer review only

For peer review only - http://bmjopen.bmj.com/site/about/guidelines.xhtml

Abstract

Objectives: No consensus exists about the best COVID-19 vaccination strategy to be adopted by low- and middle-income countries. Brazil adopted an age-based calendar strategy to reduce mortality and the burden on the healthcare system. This study evaluates the impact of the vaccination campaign in Brazil on the progression of the reported COVID-19 deaths.

Methods: This ecological study analyses the dynamic of vaccination coverage and COVID-19 deaths in hospitalised adults (≥20 years) during the first year of the COVID-19 vaccination roll-out (January-December, 2021) using nationwide data (DATASUS). We stratified the adult population into 20-49, 50-59, 60-69, and 70+ years. The dynamic effect of the vaccination campaign on mortality rates was estimated using Differences-in-Differences. The prevented and possible preventable deaths (observed deaths higher than expected) and Potential Years of Life Lost (PYLL) for each age group were obtained in a counterfactual analysis.

Results: During the first year of COVID-19 vaccination, 266,153,517 doses were administered, achieving 91% first-dose coverage. A total of 380,594 deaths were reported, 154,091 (40%) in 70+ years and 136,804 (36%) from 50-59 or 20-49 years. The mortality rates of 70+ decreased by 52% (RRR [95% CI]: 0.48 [0.43-0.53]) in 6 months, whereas rates for 20-49 were still increasing due to low coverage (52%). The vaccination roll-out strategy prevented 59,618 deaths, 53,088 (89%) from those aged 70+ years. However, the strategy did not prevent 54,797 deaths, 85% from those under 60 years, being 26,344 (45%) only in 20-49, corresponding to 1,589,271 PYLL, being 1,080,104 PYLL (68%) from those aged 20-49 years.

Conclusion: The adopted aged-based calendar vaccination strategy initially reduced mortality in the oldest but did not prevent deaths of the youngest as effectively as compared to the older age group. Countries with a high burden, limited vaccine supply and young populations should consider other factors beyond age to prioritise who should be vaccinated first.

For peer review only - http://bmjopen.bmj.com/site/about/guidelines.xhtml

Strengths and limitations of this study

- We evaluated the impact of the COVID-19 vaccination strategy in Brazil on the number of reported deaths during the campaign's first year. In 2021, 266,153,517 COVID-19 vaccine doses were administered in adults with 91% vaccination coverage.
- The vaccination strategy followed an age calendar and prioritised the high-risk groups, and following an age-based calendar, the mortality rates of the elderly population decreased earlier than those of the young population.
- Alternative strategies to an age-based calendar are necessary for countries with low-resource and young populations. These strategies should consider the local burden, vaccination supply and speed, vaccine hesitancy, and socioeconomic vulnerability.
- Non-pharmacological Interventions (NPIs) that may affect the progression of deaths and mortality rates were not considered in the analysis.
- This ecological study analysed notified data in the SIVEP-Gripe, which limits the assessment of all COVID-19 deaths and excess mortality in the country.

For peer review only

For peer review only - http://bmjopen.bmj.com/site/about/guidelines.xhtml

Introduction

The COVID-19 pandemic imposed unprecedented challenges worldwide. In 2020, the pandemic in Brazil quickly spread across the different states, with distinct patterns and burdens due to the disparities in health assistance, income and a lack of a unified national strategy to face the pandemic.[1,2] With the emergence and dominance of SARS-CoV-2-Variants of Concern (VOC)[3] at the end of 2020,[4] characterized by high transmissibility,[4] immune escape,[5,6] and increased disease severity, substantial pressure was imposed on the health system. At the beginning of 2021, when the vaccination campaign started in the country, a new surge – the second COVID-19 Gamma wave – was still rampant, with higher mortality than the first.[3]

Countries have used different strategies to define who vaccinates first.[7,8] Several studies evaluated the impact of vaccination campaigns on infections, hospitalizations and saving lives principally in high-income countries.[8–11] In Brazil, the National Immunization Program (PNI), responsible for providing the vaccination guidelines, is part of the Brazilian universal health system (*Sistema Unico de Saúde – SUS*) and has been recognized as a model of vaccination campaigns and population adhesion worldwide.[12] The Brazilian COVID-19 vaccination campaign, which started on January 17, 2021, prioritised high-risk groups and considered an age-based calendar beginning with the elderly (85 years or more).[13] Vaccine distribution accomplished the national guidelines centralizing the distribution of vaccine doses to the states and municipalities.

It is still unclear how to best prioritise groups for mass vaccination during pandemics/outbreaks such as COVID-19, especially in low- and middle-income countries (LMICs) with a high burden of the disease and a young population.[14–16] Hence, we performed an ecological study to evaluate the impact of the vaccination campaign in Brazil on the progression of the reported COVID-19 deaths. We estimated the number of prevented and possible preventable deaths during the campaign stratified by age groups.

Methods

Study Design and Participants

We conducted an ecological study to analyse the progression of reported COVID-19 deaths and mortality rates before and during the first year of the vaccination campaign roll-out in Brazil.

Daily data on reported deaths were obtained from the Influenza Epidemiological Surveillance Information System (*Sistema de Informação de Vigilância Epidemiológica da Gripe*, SIVEP-Gripe), a nationwide surveillance open-access database used to monitor severe acute

For peer review only - http://bmjopen.bmj.com/site/about/guidelines.xhtml

respiratory infections (SARI) in Brazil. SIVEP-Gripe has been the primary source of reported COVID-19 deaths in the country. This database covers all Brazilian municipalities; however, in-hospital people belong to 80% of these municipalities, where those territories correspond to 96% of Brazil's population.[2] Each register contains information about the individual's demographics, self-reported symptoms, comorbidities, hospital and ICU admission and ventilatory support, in-hospital outcome (death or discharge), and dates of symptom onset, hospital admission, and ICU admission. We included all adult participants (≥20 years old) with SARS-CoV-2-confirmed infection (RT-PCR or clinical-epidemiological criteria) whose death was reported between February 27, 2020, and December 31, 2021.

Daily data on COVID-19 vaccination coverage were obtained from the Brazilian National Immunization Program database (SI-PNI). The SI-PNI contains information on the individual's demographics (age, sex, and place of residence), vaccine dose and platform, and place and date of dose administration. We included all doses (first, second or single) administered to adults (≥20 years old) between January 17, 2021 (the start of the vaccination campaign) and December 31, 2021. Finally, we used the 2020 population estimates provided by the Brazilian Ministry of Health stratified by age.

Data were publicly available, anonymized, and de-identified. Following ethically agreed principles on open data, this analysis did not require ethical approval in Brazil. Information on the data sources is shown in online supplemental table S1.

For peer review only

Patient and public involvement

No patient was involved.

COVID-19 Vaccination Campaign Roll-Out in Brazil

On January 17, 2021, the Brazilian national immunization program (PNI) initiated the vaccination campaign for COVID-19. Vaccine doses were freely distributed to states and municipalities, which managed the administration according to the availability of resources. The COVID-19 vaccination campaign strategy followed a national age-based calendar during the whole period, with some local adaptations. It started in parallel vaccinating those aged 90+, indigenous individuals, healthcare workers, and high-risk groups based on comorbidities and then followed an exclusively age-based calendar. Four vaccine platforms were considered in the country: two

For peer review only - http://bmjopen.bmj.com/site/about/guidelines.xhtml

doses of Sinovac-CoronaVac, ChAdOx1-S/nCoV-19 (Oxford-AstraZeneca), or BNT162b2 (Pfizer/BioNTech), or Ad26.COV2.S (Janssen) single-dose.

Outcomes

Our primary outcome was the estimated number of prevented COVID-19 deaths. Secondary outcomes comprise possible preventable deaths, the potential years of life lost (PYLL), the mortality rates per 100,000 population, and the first dose and second or single dose vaccination coverage. All quantities, rates, and estimates were evaluated temporally for the whole country and stratified by age groups; no further stratification was performed.

Data Analysis

We described continuous variables using medians and interquartile ranges (IQRs) or means and standard deviations (SDs) when applicable and frequencies and proportions for categorical variables. The analysis was pre-specified, and the sample size was pragmatic (SIVEP-Gripe database). All analyses considered complete data, and no imputation of missing values was performed.

We evaluated the progression of COVID-19 deaths before and during the vaccination campaign (February 26, 2020, to December 31, 2021) for the whole country and stratified by age groups. We generated the time series of daily reported deaths and mortality rates using the 15-day moving average to reduce the effects of variability in the notification process. Changes in the representativeness of age groups in the deaths were assessed by calculating the proportion of each group in the daily count. In addition, vaccination coverage of at least one dose ("partially vaccinated") and two or single doses ("fully vaccinated") was calculated as the number of doses divided by the corresponding estimated population.

To estimate the impact of the vaccination campaign strategy on the evolution of daily reported mortality rates for each age group (20-49, 50-59, 60-69, and 70+), we performed a three-stage analysis.

First, we estimated the dynamic effects of the vaccination campaign using a differences-in-differences (DiD) analysis.[17] DiD is a causal model approach used for impact evaluation by comparing treatment and control groups, before and after an intervention, under non-experimental settings (e.g., non-randomized data).[18] We considered reported COVID-19 deaths as the response variable. Each age group was considered the "treatment variable" and

For peer review only

the start of the vaccination campaign roll-out (January 17, 2021) was the intervention onset indicator for all groups. The control group is represented by the national age-adjusted mortality COVID-19 rates in Brazil as a surrogate of the pandemic progression in the country during the vaccination campaign. We compared the daily mortality rates of each age group and the control group using the Rate Ratio (RR). Due to the lack of a proper control group to perform the analysis, using the national age-adjusted mortality could be considered as an alternative to a synthetic control to overcome this difficulty.

Afterwards, we applied a Negative Binomial Regression model to estimate the intervention effect. The response variable (y_i) was the reported deaths, and the covariates were the intervention (age) / control group ($x_{age,t}$), the intervention period indicator ($x_{period,t}$), their interaction ($x_{age,t} * x_{period,t}$), and the population ($x_{population}$) as the offset variable. To obtain the dynamic effects for each age group, we split the intervention period variable into separate indicators, one for each month. The intervention effect was calculated as the exponentiated coefficient of the interaction terms, defined as the ratio of rate ratios (RRR) [19] or the DiD estimator, and their corresponding 95% confidence intervals. The Negative Binomial Regression model is represented as follows:

$$\ln y_i = \beta_0 + \beta_1 x_{age,t} + \beta_2 x_{period,t} + \beta_3 x_{age,t} * x_{period,t} + offset(x_{population}) \quad (1)$$

where t represents the monthly observations and β_0, β_1, β_2 and β_3 are regression coefficients.

Subsequently, we quantified the impact of the vaccination strategy in terms of prevented and possible preventable deaths for each age group using a counterfactual analysis.[20] Using a Linear Regression model, we predicted the mortality rate of an age group (response variable, y_i) based on the national age-adjusted mortality rates in the country (predictor variable, x_i).[20] The model is expressed by:

$$y_i = \beta_0 + \beta_1 x_i + \varepsilon_i \quad (2)$$

where ε_i denotes the error term, i is the daily observations of the target age group and β_0 and β_1 are regression coefficients. Although the national age-adjusted mortality rates in the country do not strictly represent a scenario without vaccination, we could obtain the number of expected deaths considering this progression as being the dynamic pandemic progression and the vaccination roll-out as the speed and effectiveness in the decrease of deaths for each age group.

For peer review only - http://bmjopen.bmj.com/site/about/guidelines.xhtml

For peer review only

We considered the start of the vaccination roll-out as the date of intervention onset (January 17, 2021). Hence, we fit the model using data prior to the vaccination roll-out to obtain the of predicted mortality rates for each age group in the following period. We multiplied the predicted mortality rates by the total population to obtain the number of expected deaths. When comparing the number of expected to observed deaths for each age group, we considered "prevented deaths" as when the observed deaths were lower than the expected deaths; otherwise, we considered "possible preventable deaths".

Furthermore, we estimated the corresponding number of prevented and preventable Potential Years of Life Lost (PYLL) for each age group, considering the midpoint of each age group and a life expectancy of 76 years for Brazil in 2019.[21] All analyses were performed in R 4.2.1. We followed STROBE recommendations.

Role of the Funding Source

The funders had no role in any decision about the manuscript. All authors had full access to all the data in the study. SA, PM, FB, and LSLB verified the data, and all authors approved the final version of the manuscript for publication.

Results

Age-Adjusted Mortality and Vaccine Coverage in the First Year of Vaccine Roll-Out

From February 26, 2020, to December 31, 2021, 565,774 COVID-19 deaths were reported (**figure 1-A**). Most of these comprised those aged 70 years or older (253,092, 45%), and one-third were those aged under 60 years (**figure 1-B**). The daily average mortality rates per 100,000 population were the highest between March and Mai 2021 for all most the age groups and occurred during the dominance of VOC Gamma (**online supplemental figure S1 and table S2**). When comparing the proportion of reported deaths (**figure 1-C**), the 70+ age group showed a decrease since March 2021, two months after the vaccination roll-out start, being the lowest in May-June 2021, almost 50% lower compared to pre-vaccination period. During this period, the proportion of deaths related to 20-49 and 50-59 years increased. However, in late 2021, there was a gradual reduction in the proportion of deaths related to those under 60 years.

With respect to the vaccination roll-out, 266,153,517 vaccine doses for COVID-19 were administered in adults (≥20 years old) in 2021, with 137,644,737 (51.66%) first doses and

For peer review only - http://bmjopen.bmj.com/site/about/guidelines.xhtml

For peer review only

128,508,780 (48.34%) second or single doses. At the end of 2021, the national first dose vaccination coverage for adults was 90.7%, with 86.74% for 20-49 years, 94.75% for 50-59 years, 100.78% for 60-69 years, and 99.59% for 70+ years (**online supplemental tables S3 and S4**). The progression of vaccination coverage rates was different for each age group due to the campaign strategy (**figure 1-D**). Brazil achieved 75% vaccination coverage in August 2021, six months after the vaccination roll-out. In the same period, the 70+ group had 98% first-dose coverage, whereas the 20-49 group presented 52% coverage (**online supplemental table S3**). We observed that the oldest group (70+) already had more than 90% vaccination coverage by the end of March 2021. Similar behaviour for the second or single dose was observed (**online supplemental figure S2 and table S4**).

Vaccination Effect on Mortality Rates

To assess the impact of the vaccination campaign strategy on reducing mortality rates, we compared the mortality rates for each age group with the national age-adjusted mortality rates in the country (**figure 2-A**). The 70+ group showed the highest mortality rates in the whole period (Rate Ratio > 1). However, six months later the vaccination roll-out started, the mortality rates reduced from 3.84 deaths per 100,000 population (Rate Ratio: 6.16) in January 2021 to 3.31 deaths per 100,000 (Rate Ratio: 3.11) in June 2021 (**online supplemental table S2**). In the same period, the 20-49 age group showed an increase in mortality rates (Rate Ratio: 0.21 in January 2021 and 0.58 in June 2021) compared to the national age-adjusted mortality rates. After September 2021, the Rate Ratios for all age groups returned to similar levels to those seen before vaccination (**online supplemental table S2**).

We estimated the intervention effect using a Negative Binomial Regression model to evaluate the association between the reported deaths, the intervention (age)/ control group, and the intervention period indicator (**figure 2-B**). When analysing the temporal progression of the RRR stratified by age group (**online supplemental table S5**), the 70+ age group had the lowest rates in June 2021 (RRR [95% CI]: 0.48 [0.43-0.53]), which evidenced a 52% decrease in the number of deaths in 5 months. With regard to age groups 50-59 and 60-69, we estimated a decrease in death rates of 15% and 30% (RRR [95% CI]: 0.85 [0.75-0.96] and RRR [95% CI]: 0.70 [0.63-0.71], respectively) for at least 4 and 6 months in the first year of vaccination (**online supplemental table S5**).

For peer review only - http://bmjopen.bmj.com/site/about/guidelines.xhtml

Number of Prevented and Possible Preventable Deaths and Potential Years of Life Lost (PYLL)

We quantified the number of prevented and possible preventable deaths in each age group during the vaccination roll-out (**figure 3**). We estimated that vaccination prevented 59,618 deaths compared to the expected deaths in the first year of vaccination roll-out (**table 1**). Of these, 89% were prevented in those aged 70 years or more (53,088), followed by those aged 60-69 years (6,032, 10%), which achieved high vaccination coverage earlier than younger age groups (**figure 1-D**).

Table 1. Comparison between observed and predicted deaths during the vaccination campaign in Brazil in 2021, stratified by age group.

Results	Age group				Total
	**20-49**	**50-59**	**60-69**	**70+**	
Observed deaths	65,582	67,991	86,126	145,812	365,511
Expected deaths	39,393	48,098	85,064	197,777	370,332
Prevented deaths	155	343	6,032	53,088	59,618
Possible preventable deaths	26,344	20,236	7,094	1,123	54,797
Prevented PYLL[#] †	6,355	7,203	66,352	291,984	371,894
Preventable PYLL[#] †	1,080,104	424,956	78,034	6,177	1,589,271

Prevented deaths were obtained if observed deaths were lower than predicted, and possible preventable deaths, were obtained if the number of observed deaths was higher than predicted.
†The denominator is the adult population of Brazil for this age group = 151,778,738.
[#] PYLL was calculated using the average age for each group and a life expectancy of 76 years for Brazil in 2020.

We considered possible preventable deaths when the number of observed deaths was higher than those expected if each age group followed the progression of the national age-adjusted mortality rates in the country (**figure 3, table 1**). Despite the considerable number of deaths prevented, we estimated 54,797 potentially preventable deaths in the same period. More than 85% of possible preventable deaths comprised those under 60 years old, half of them corresponding to those aged 20-49 years (26,344, 48%), which achieved late vaccination coverage compared to older groups (**figure 1-D**).

In addition, we estimated the impact of the vaccination roll-out in terms of the Potential Years of Life Lost (PYLL). The vaccination strategy prevented 371,894 PYLL, especially for those aged 60 years or older. However, it was not able to prevent 1,589,271 PYLL, composed mainly of those aged 20-49 years (68%) (**table 1**).

For peer review only - http://bmjopen.bmj.com/site/about/guidelines.xhtml

Discussion

We evaluated the impact of the COVID-19 vaccination strategy in Brazil on the number of reported deaths during the campaign's first year by analysing the progression of vaccination coverage and reported deaths and model the prevented and possible preventable deaths. In prioritising the vaccination of high-risk groups, the mortality rates of the elderly population reduced earlier compared to the young population, especially in a period of high burden due to the introduction of a new VOCs. After 6 months of vaccination roll-out, the vaccination coverage for those aged 70+ years was 98% compared to only 52% for the age group 20-49, which resulted in a reduction of 44%, and the mortality rates in the 70+ years and for those aged 20-49 were still increasing due to the low coverage. At the end of 2021, the first dose coverage achieved 91%, and the estimated number of prevented deaths was 59,618. However, the delay in the vaccination of younger ages may have resulted in an expressive number of deaths that could be prevented in this group.

Brazil was one of the epicentres of COVID-19 deaths in 2020, which intensified in a second and larger surge at the beginning of 2021 when the COVID-19 vaccination campaign started.[3] The first months comprised limited vaccine dose availability until May 2021. The strategy prioritised the immunization of high-risk groups (i.e., indigenous, healthcare workers, and comorbidities) and older ages, intending to reduce the burden of deaths and overload over the health system. This strategy resulted in a rapid decrease in mortality rates in the country, driven mainly by those aged 70+, which displayed the highest number of prevented deaths (53,088) in the period. The strategy successfully prevented deaths in the older population; however, the major drawback was the expressive number of deaths in those aged < 60 years old. Although non-pharmacological interventions (NPIs) such as social distancing and masks mandates have been implemented in Brazil, there was not a coordinated and unified guideline at the national level. Due to the country heterogeneity, municipalities applied NPIs under different flags, but not with a high degree of adherence; despite this when the vaccination campaign began none NPIs continued.

Except for those with high-risk conditions, vaccination for younger groups started only four to six months after vaccination for older groups, during the third surge of deaths (July 2021). Although mortality risks for this population are lower compared to the elderly, Brazil is composed mainly of young people. At the end of 2021, 86,834 deaths were reported for the 20-49 age group, where 66,692 (77%) occurred during the vaccination roll-out period. The trend of mortality for the youngers started to decrease in July 2021 and was more pronounced in

For peer review only - http://bmjopen.bmj.com/site/about/guidelines.xhtml

September 2021, when vaccination coverage of this population was higher than 75%. Our analysis did not discriminate between prioritised high-risk groups from those individuals that following the age-based calendar in the vaccination campaign, since this is a low percentage of doses from these groups, especially those based on comorbidities lower than 10%, hence not significantly impacting the results or the progression of the vaccination campaign.

We estimated that the vaccination strategy prevented 59,618 deaths, composed mainly of those aged 60-69 or 70+ years. However, 54,797 possible preventable deaths of those aged 50-59 or 20-49 years occurred, corresponding to a potential savings of 1,589,271 PYLL, 68% from 20-49 years. These results align with previous studies that identified a reduction in deaths and hospitalizations due to vaccination in high-income countries.[8–11] A recent Brazilian study obtained 303,129 prevented deaths in the period from January 17, 2021 to January 31, 2022, with 61% from those aged >= 60 and 7% for the 20-39 age group.[22] Our estimates were obtained using a counterfactual analysis in which the control group represented the progression of national age-adjusted mortality rates during the vaccination campaign. Although it does not represent a scenario strictly without vaccination, we could obtain the number of expected deaths considering the dynamics in pandemic progression and the vaccination roll-out as the speed and effectiveness in the decrease of deaths for each age group. Hence, we were able to evaluate the results from the age-based calendar strategy and quantify the number of prevented and possible preventable deaths for each age group during the first year of the vaccination campaign.

Latin America was highly affected by the pandemic, the continent with the highest number of deaths per capita, with limited access to vaccine doses.[23,24] In Brazil, the availability of vaccine doses at the beginning of the vaccination roll-out was low due to the national government's late negotiation for vaccine purchases, global demand, and the high concentration of doses in rich countries.[13,25] The concern with respect to vaccine hesitancy, encouraged by the country's leadership and polarized political environment, was not confirmed by previous evidence and high vaccination coverage was achieved.[13,25–27] The urgency of vaccination also refers to better use of a common resource, including access to vaccines, prioritising care for those most affected, i.e., where the epidemic is behaving most intensely.[28]

Recently, the WHO revised the initial recommendation of prioritising the years of life saved to reduce deaths and disease burden, especially in scenarios of limited access to vaccines.[28] In Brazil, due to the low availability of vaccine doses at the start of the roll-out, a strategy that followed an age-based calendar also prioritising high-risk groups was adopted. Our

For peer review only - http://bmjopen.bmj.com/site/about/guidelines.xhtml

results demonstrated that the strategy protected the oldest. However, it could not significantly prevent the deaths of the youngest. These findings raise concerns about whether the age-based calendar is the best strategy for countries with low-resource settings and a young population[29]. Should alternative strategies prioritise locals with high burden and increase the years of life saved in locations with limited healthcare resources and socioeconomic vulnerability? Alternative strategies to the age-based calendar have been proposed pointing out the importance of considering the burden, age distribution, vaccination supply and speed, hesitancy, and socioeconomic vulnerability to promote a better and more equitable decision-making process.[14,15,28,30,31] Other model approaches, such as agent-based or deterministic compartmental models, may also assess the potential gains of alternative strategies.[15,16]

The findings of this study should be interpreted in light of some potential limitations. First, we analysed data notified in the SIVEP-Gripe, which consists of cases reported by the surveillance system. This limits the assessment of all COVID-19 deaths and excess mortality in the country. However, the SIVEP-Gripe has been one of the country's central repositories of COVID-19 epidemiological data, with high information coverage[2]. Second, stringent policies and actions, as well as personal behaviour risk, may affect the progression of deaths and mortality rates and were not considered in the analysis. Third, although the vaccination roll-out started at the same time across the country, regional differences can indeed impact the vaccine coverage speed due to limited access or the age composition of certain locations.[13] Fourth, we did not take into account for previous infection, once the elderly had the highest rate of infection early in the pandemic, the natural immunity in this group might have also contributed to the same protection observed against death. Furthermore, the study has strengths: this is a nationwide data analysis of a large LMIC with a high burden of COVID-19; we estimate the dynamic impact of the vaccination program in four age groups considering an average behaviour, which allows the assessment and understanding of the vaccination roll-out within the country.

Conclusion

Our findings indicate that the vaccination campaign was able to prevent more than 59,000 deaths in Brazil, especially for the older population. However, the delay in the vaccination of younger ages may have resulted in an expressive number of deaths that could be prevented in this group.

For peer review only - http://bmjopen.bmj.com/site/about/guidelines.xhtml

Data availability statement

Data are available on reasonable request.

Ethics statements

Patient consent for publication

Not applicable.

Acknowledgements

This work is part of the Grand Challenges ICODA pilot initiative, delivered by Health Data Research UK and funded by the Bill & Melinda Gates Foundation and the Minderoo Foundation. This study was also supported by the National Council for Scientific and Technological Development (CNPq), the Coordination for the Improvement of Higher Education Personnel (CAPES) - Finance Code 001, Carlos Chagas Filho Foundation for Research Support of the State of Rio de Janeiro (FAPERJ), the Pontifical Catholic University of Rio de Janeiro. OTR is funded by a Sara Borrell grant from the Instituto de Salud Carlos III (CD19/00110). OTR acknowledges support from the Spanish Ministry of Science and Innovation and State Research Agency through the "Centro de Excelencia Severo Ochoa 2019-2023" Program (CEX2018-000806-S) and support from the Generalitat de Catalunya through the CERCA Program.

We are grateful to all healthcare workers for their endless efforts to tackle the COVID-19 pandemic in Brazil. We thank the NOIS research group and the ICODA driver project EFFECT-Brazil team members for the discussions and collaborative production during the COVID-19 pandemic. All authors carried out the research independently of the funding body. The findings and conclusions in this manuscript reflect the opinions of the authors alone.

For peer review only - http://bmjopen.bmj.com/site/about/guidelines.xhtml

Contributors

SA, LSLB, PM, FB, PS, JCN, OTR, SH and FAB participated in the design and concept of the study. SA, LSLB and PM did the data curation. SA, LSBL, PM, FB, OTR and FAB designed the data analysis. SA, LSLB, PM, FB, PS, SH and FAB performed the data analysis. SA, LSLB, PM, FB and FAB wrote the first version of the manuscript. LSLB, PM, FB, SH and FAB supervised the study. All authors had full access to the data, participated in data interpretation, revised the manuscript, and approved the final version of the manuscript.

Funding

This work is part of the Grand Challenges ICODA pilot initiative, delivered by Health Data Research UK and funded by the Bill & Melinda Gates Foundation (INV-017293) and the Minderoo Foundation. This study was supported by the National Council for Scientific and Technological Development (CNPq), the Coordination for the Improvement of Higher Education Personnel (CAPES) - Finance Code 001, Carlos Chagas Filho Foundation for Research Support of the State of Rio de Janeiro (FAPERJ) and the Pontifical Catholic University of Rio de Janeiro. OTR is funded by a Sara Borrell grant from the Instituto de Salud Carlos III (CD19/00110). OTR acknowledges support from the Spanish Ministry of Science and Innovation and State Research Agency through the "Centro de Excelencia Severo Ochoa 2019-2023" Program (CEX2018-000806-S), and support from the Generalitat de Catalunya through the CERCA Program.

All authors carried out the research independently of the funding bodies. The findings and conclusions in this manuscript reflect the opinions of the authors alone.

Competing interests

SH and FAB are funded by the CNPq and FAPERJ. PM is funded by CNPq (422470/2021-0) and FAPERJ (E-26/211.645/2021 and E-26/201.348/2022). OTR is funded by a Sara Borrell fellowship from the Instituto de Salud Carlos III (CD19/00110), acknowledges support from the Spanish Ministry of Science and Innovation and State Research Agency through the "Centro de Excelencia Severo Ochoa 2019–2023" Program (CEX2018-000806-S), support from the Generalitat de Catalunya through the CERCA Program, and received a research grant from the Health Effects Institute for research unrelated to this manuscript. OTR was also member of the Data Safety Monitoring Board in the REVOLUTION and STOP-COVID trials, testing treatments

For peer review only - http://bmjopen.bmj.com/site/about/guidelines.xhtml

against COVID-19, and is currently member of the Data Safety Monitoring Board of the RENOVATE trial, testing respiratory support strategies in patients with acute respiratory hypoxaemic failure. The other authors declare that they have no conflicts of interest.

All authors carried out the research independently of the funding bodies. The findings and conclusions in this manuscript reflect the opinions of the authors alone.

Data sharing

All data, including individual participant data, is publicly available with de-identification and anonymisation of patients. The data sources are described in the manuscript and in the supplementary material (**online supplemental table S1**). The raw data and code used for the analysis are available in a GitHub repository, with publication (https://github.com/noispuc/ICODA_COVID_VaccineStrategy)

For peer review only

For peer review only - http://bmjopen.bmj.com/site/about/guidelines.xhtml

References

1 Castro MC, Kim S, Barberia L, *et al.* Spatiotemporal pattern of COVID-19 spread in Brazil. *Science (80-)* 2021;**372**:821–6. doi:10.1126/science.abh1558

2 Ranzani OT, Bastos LSL, Gelli JGM, *et al.* Characterisation of the first 250 000 hospital admissions for COVID-19 in Brazil: a retrospective analysis of nationwide data. *Lancet Respir Med* 2021;**9**:407–18. doi:10.1016/S2213-2600(20)30560-9

3 Bastos LS, Ranzani OT, Souza TML, *et al.* COVID-19 hospital admissions: Brazil's first and second waves compared. *Lancet Respir Med* 2021;**9**:e82–3. doi:10.1016/S2213-2600(21)00287-3

4 Faria NR, Mellan TA, Whittaker C, *et al.* Genomics and epidemiology of the P.1 SARS-CoV-2 lineage in Manaus, Brazil. *Science (80-)* 2021;**372**:815–21. doi:10.1126/science.abh2644

5 Sabino EC, Buss LF, Carvalho MPS, *et al.* Resurgence of COVID-19 in Manaus, Brazil, despite high seroprevalence. *Lancet* 2021;**397**:452–5. doi:https://doi.org/10.1016/S0140-6736(21)00183-5

6 Planas D, Saunders N, Maes P, *et al.* Considerable escape of SARS-CoV-2 Omicron to antibody neutralization. *Nature* 2022;**602**:671–5. doi:10.1038/s41586-021-04389-z

7 Voigt A, Omholt S, Almaas E. Comparing the impact of vaccination strategies on the spread of COVID-19, including a novel household-targeted vaccination strategy. *PLoS One* 2022;**17**:1–16. doi:10.1371/journal.pone.0263155

8 Kim D, Lee YJ. Vaccination strategies and transmission of COVID-19: Evidence across advanced countries. *J Health Econ* 2022;**82**:102589. doi:10.1016/j.jhealeco.2022.102589

9 Shoukat A, Vilches TN, Moghadas SM, *et al.* Lives saved and hospitalizations averted by COVID-19 vaccination in New York City: a modeling study. *Lancet Reg Heal - Am* 2022;**5**:100085. doi:10.1016/j.lana.2021.100085

10 Steele MK, Couture A, Reed C, *et al.* Estimated Number of COVID-19 Infections, Hospitalizations, and Deaths Prevented among Vaccinated Persons in the US, December 2020 to September 2021. *JAMA Netw Open* 2022;**5**:E2220385. doi:10.1001/jamanetworkopen.2022.20385

11 Ferreira LS, Marquitti FMD, Silva RLP da, *et al.* Estimating the impact of implementation and timing of COVID-19 vaccination program in Brazil: a counterfactual analysis. *medRxiv* 2021;:2021.12.24.21268384.https://www.medrxiv.org/content/10.1101/2021.12.24.21268384v1%0Ahttps://www.medrxiv.org/content/10.1101/2021.12.24.21268384v1.abstract

12 Peres KC, Buendgens FB, Prates EA, *et al.* Vacinas no Brasil: análise histórica do registro sanitário e a disponibilização no Sistema de Saúde. *Cien Saude Colet* 2021;**26**:5509–22. doi:10.1590/1413-812320212611.13932021

13 Bastos LS, Aguilar S, Rache B, *et al.* Primary healthcare protects vulnerable populations from inequity in COVID-19 vaccination: An ecological analysis of nationwide data from Brazil. *Lancet Reg Heal* 2022;:100335.

For peer review only - http://bmjopen.bmj.com/site/about/guidelines.xhtml

14 McIntyre PB, Aggarwal R, Jani I, *et al.* COVID-19 vaccine strategies must focus on severe disease and global equity. *Lancet* 2022;**399**:406–10. doi:10.1016/S0140-6736(21)02835-X

15 Saadi N, Chi YL, Ghosh S, *et al.* Models of COVID-19 vaccine prioritisation: a systematic literature search and narrative review. *BMC Med* 2021;**19**:1–11. doi:10.1186/s12916-021-02190-3

16 Liu Y, Sandmann FG, Barnard RC, *et al.* Optimising health and economic impacts of COVID-19 vaccine prioritisation strategies in the WHO European Region: a mathematical modelling study. *Lancet Reg Heal - Eur* 2022;**12**:100267. doi:10.1016/j.lanepe.2021.100267

17 Karaivanov A, Kim D, Lu SE, *et al.* COVID-19 vaccination mandates and vaccine uptake. *Nat Hum Behav* 2022;:1–10. doi:10.1038/s41562-022-01363-1

18 Fredriksson A, Oliveira GM de. Impact evaluation using Difference-in-Differences. *RAUSP Manag J* 2019;**54**:519–32. doi:10.1108/RAUSP-05-2019-0112

19 Matthay EC, Farkas K, Goin DE, *et al.* Associations of firearm dealer openings with firearm self-harm deaths and injuries: A differences-in-differences analysis. *PLoS One* 2021;**16**:1–11. doi:10.1371/journal.pone.0248130

20 Izbicki R, Bastos LS, Izbicki M, *et al.* How many hospitalizations has the covid-19 vaccination already prevented in são paulo? *Clinics* 2021;**76**:3–4. doi:10.6061/clinics/2021/e3250

21 Mitra AK, Payton M, Kabir N, *et al.* Potential years of life lost due to COVID-19 in the United States, Italy, and Germany: An old formula with newer ideas. *Int J Environ Res Public Health* 2020;**17**:1–9. doi:10.3390/ijerph17124392

22 Dos Santos CVB, de Noronha TG, Werneck G, *et al.* Estimated COVID-19 Severe Cases and Deaths Averted in the First Year Of the Vaccination Campaign in Brazil: A Retrospective Observational Study. *Lancet Reg Heal* 2023;**17**:100418. doi:10.2139/ssrn.4151508

23 Cifuentes-Faura J. COVID-19 mortality rate and its incidence in Latin America: Dependence on demographic and economic variables. *Int J Environ Res Public Health* 2021;**18**.

24 Hotez PJ, Narayan KMV. Sharing Technology and Vaccine Doses to Address Global Vaccine Inequity and End the COVID-19 Pandemic. *JAMA - J Am Med Assoc* 2021;**326**:219–20. doi:10.1001/jama.2021.7439

25 Fonseca EM da, Shadlen KC, Bastos FI. The politics of COVID-19 vaccination in middle-income countries: Lessons from Brazil. *Soc Sci Med* 2021;**281**:114093. doi:10.1016/j.socscimed.2021.114093

26 Gramacho WG, Turgeon M. When politics collides with public health: COVID-19 vaccine country of origin and vaccination acceptance in Brazil. *Vaccine* 2021;**39**:2608–12. doi:10.1016/j.vaccine.2021.03.080

27 Moore DCBC, Nehab MF, Camacho KG, *et al.* Low COVID-19 vaccine hesitancy in Brazil. *Vaccine* 2021;**39**:6262–8. doi:10.1016/j.vaccine.2021.09.013

28 Faden R, Craviotto A, Hombach J, *et al.* Who to vaccinate first? A peek at decision-making in a pandemic. *Nature* 2022;**607**:235–8. doi:10.1038/d41586-022-01899-2

For peer review only - http://bmjopen.bmj.com/site/about/guidelines.xhtml

29 Watson OJ, Barnsley G, Toor J, *et al.* Global impact of the first year of COVID-19 vaccination: a mathematical modelling study. *Lancet Infect Dis* 2022;**22**:1293–302. doi:10.1016/S1473-3099(22)00320-6

30 Hogan AB, Winskill P, Watson OJ, *et al.* Within-country age-based prioritisation, global allocation, and public health impact of a vaccine against SARS-CoV-2: A mathematical modelling analysis. *Vaccine* 2021;**39**:2995–3006. doi:10.1016/j.vaccine.2021.04.002

31 Sachs JD, Karim SSA, Aknin L, *et al.* The Lancet Commission on lessons for the future from the COVID-19 pandemic. *Lancet* 2022;**400**.

For peer review only

For peer review only - http://bmjopen.bmj.com/site/about/guidelines.xhtml

Figure legends

Figure 1. Progression of COVID-19 reported deaths and the vaccination roll-out in Brazil: (A) Daily number of reported deaths (15-day moving average, left y-axis) and first dose vaccine coverage (right y-axis) from February 26, 2020 to December 31, 2021; yellow shaded areas display the period of VOC dominance (50% of samples using oubreak.info/GISAID data) for Gamma (February 15, 2021) and Delta (August 11, 2021) - vertical dashed lines indicate the start of the vaccination roll-out in Brazil and the 75% first vaccination coverage of adult population, respectively; (B) Daily number reported deaths stratified by age group in 2021; (C) Daily proportions of deaths reported for each age group (number of deaths / total number of deaths per day); (D) First dose vaccination coverage stratified by age group - vertical dashed lines refer to the start of the vaccination campaign for each age group, the horizontal dashed line indicates the 75% vaccination coverage target by the World Health Organization (WHO).

Figure 2. The impact of the COVID-19 vaccination campaign in reported deaths stratified by age groups: (A) Rate ratios comparing daily mortality rates stratified by age group with the national mortality rate; (B) Estimated effect of the vaccination campaign in each age groups from the difference-in-differences (DiD) analysis. Effects were obtained as the Ratio of Rate Ratios (RRR) and their respective 95% confidence intervals. The vertical dashed line refers to the start of the vaccination campaign on January 17, 2021. DiD analysis considered six months before the vaccination roll-out as the baseline period and the national mortality rates as the control group in a Negative Binomial Regression model.

Figure 3. Estimated prevented and possible preventable deaths stratified by age group. For each age group (coloured lines), the predicted deaths (black lines) were obtained whether that age-group followed the national age-adjusted mortality rates. Grey lines correspond to the fitted linear model. Predicted deaths were estimated from the period when 10% of first dose vaccination coverage was achieved for each age group. Prevented deaths (green-shaded area) were obtained if observed deaths was lower than predicted, and possible preventable deaths (red-shaded area), if the number of observed deaths was higher than predicted. The vertical dashed lines refer to the start of the vaccination campaign on January 17, 2021.

For peer review only

For peer review only - http://bmjopen.bmj.com/site/about/guidelines.xhtml

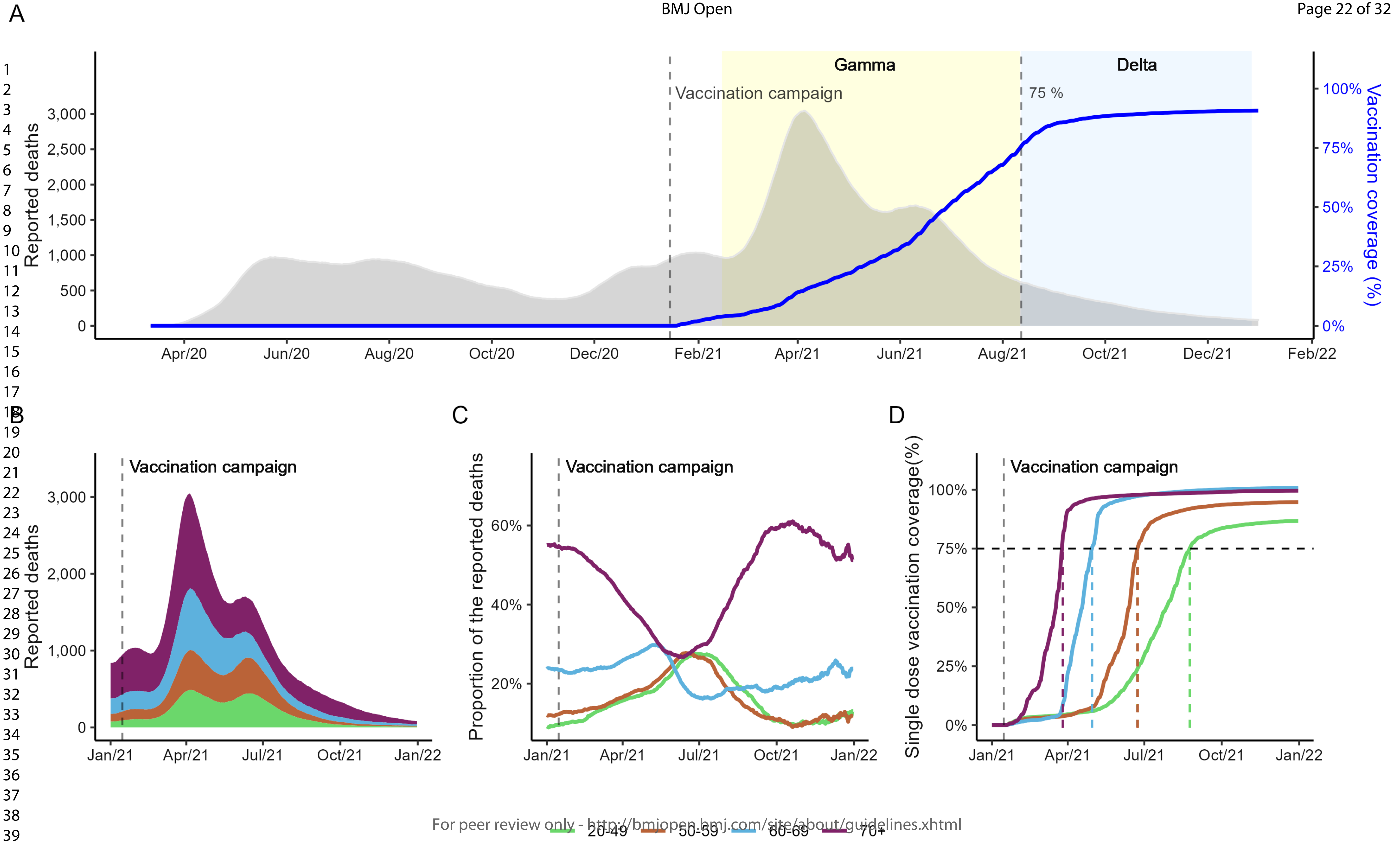

A
Gamma
Delta
Vaccination campaign
75 %
Reported deaths
3,000
2,500
2,000
1,500
1,000
500
0
100%
75%
50%
25%
0%
Vaccination coverage (%)
Apr/20
Jun/20
Aug/20
Oct/20
Dec/20
Feb/21
Apr/21
Jun/21
Aug/21
Oct/21
Dec/21
Feb/22
B
C
D
Vaccination campaign
Reported deaths
3,000
2,000
1,000
0
Proportion of the reported deaths
60%
40%
20%
Single dose vaccination coverage(%)
100%
75%
50%
25%
0%
Jan/21
Apr/21
Jul/21
Oct/21
Jan/22
20-49
50-59
60-69
70+

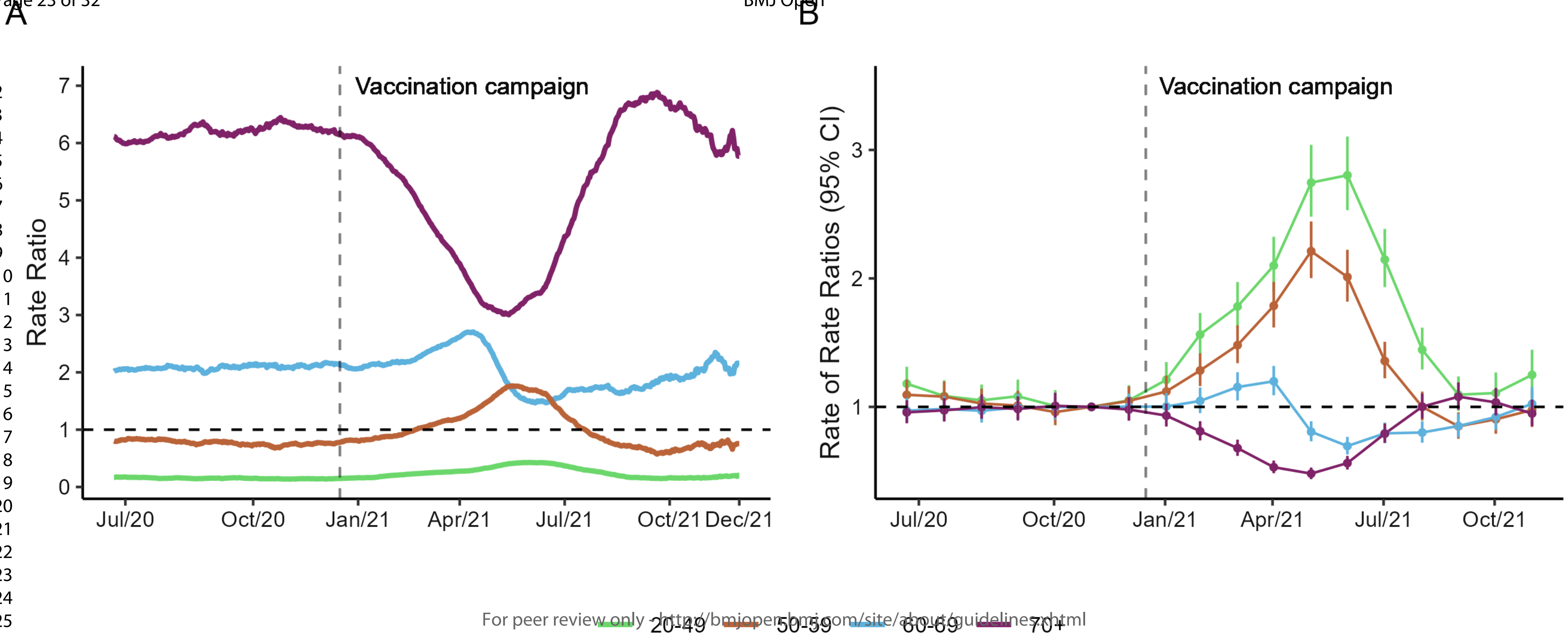
A
Vaccination campaign
Rate Ratio
0
1
2
3
4
5
6
7
Jul/20
Oct/20
Jan/21
Apr/21
Jul/21
Oct/21
Dec/21
B
Vaccination campaign
Rate of Rate Ratios (95% CI)
1
2
3
Jul/20
Oct/20
Jan/21
Apr/21
Jul/21
Oct/21
20-49
50-59
60-69
70+

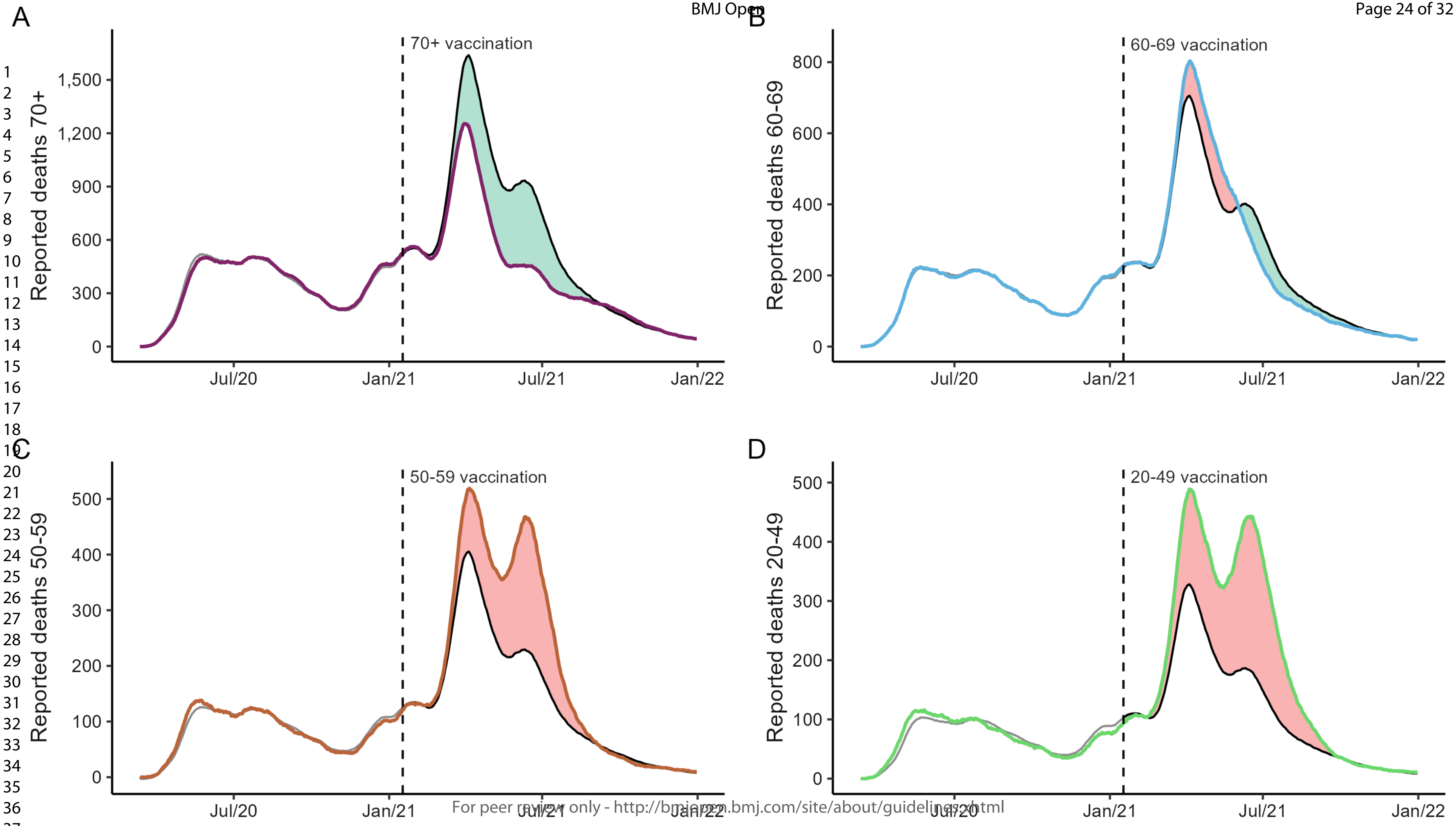
A
70+ vaccination
Reported deaths 70+
1,500
1,200
900
600
300
0
Jul/20
Jan/21
Jul/21
Jan/22
B
60-69 vaccination
Reported deaths 60-69
800
600
400
200
0
Jul/20
Jan/21
Jul/21
Jan/22
C
50-59 vaccination
Reported deaths 50-59
500
400
300
200
100
0
Jul/20
Jan/21
Jul/21
Jan/22
D
20-49 vaccination
Reported deaths 20-49
500
400
300
200
100
0
Jul/20
Jan/21
Jul/21
Jan/22

Supplementary Material

Table of contents

Figure S1. Deaths per 100.000 pop stratified by age group – The vertical dashed line refers to the start of the vaccination campaign for each age group.. 3
Figure S2. (A) First dose vaccination coverage stratified by age group – first vertical dashed line on the left refers to the start of the vaccination campaign for each age group, the horizontal dashed line indicates the 75% vaccination coverage target by the World Health Organization (WHO). (B) Second or single vaccination coverage stratified by age group.. 3

Table S1. Description of the data sources used in this study 2
Table S2. Monthly average of the mortality ratios in Brazil, in 2021, stratified by age group. 4
Table S3. Number of reported deaths and first dose vaccine coverage per month in during the COVID-19 vaccination campaign in Brazil (started in January 17, 2021). 5
Table S4. Second or single vaccination coverage during the vaccination roll-out in Brazil, in 2021, stratified by age group. 6
Table S5. Monthly and global estimated effect of the vaccination campaign (DID analysis) with their respective 95% confidence intervals stratified by age group. 7

For peer review only

Table S1. Description of the data sources used in this study

Data	Source	Source address	Version	Date exported
COVID-19 Vaccination doses	National Immunisation Program Information System,“SI-PNI”	*OpenDataSUS* repository: https://opendatasus.saude.gov.br/dataset/covid-19-vacinacao	21/07/2022	21/07/2022
Brazilian population estimates (per age, sex, and municipalities)	Health Surveillance Secretary from the Brazilian Ministry of Health	Official website: http://tabnet.datasus.gov.br/cgi/tabcgi.exe?popsvs/cnv/popbr.def	2020	07/02/2022
COVID-19 hospitalized cases	Influenza Epidemiological Surveillance Information System, “SIVEP-Gripe”	*OpenDataSUS* repository: https://opendatasus.saude.gov.br/dataset/bd-srag-2020 https://opendatasus.saude.gov.br/dataset/srag-2021-e-2022	21/07/2022	21/07/2022
Life expectancy at birth, total (years) - Brazil	World Bank	Official website: https://data.worldbank.org/indicator/SP.DYN.LE00.IN?locations=BR&most_recent_year_desc=true	2019	25/07/2022

COVID-19: Disease caused by the SARS-CoV-2 virus.

For peer review only

Figure S1. Deaths per 100.000 pop stratified by age group – The vertical dashed line refers to the start of the vaccination campaign for each age group.

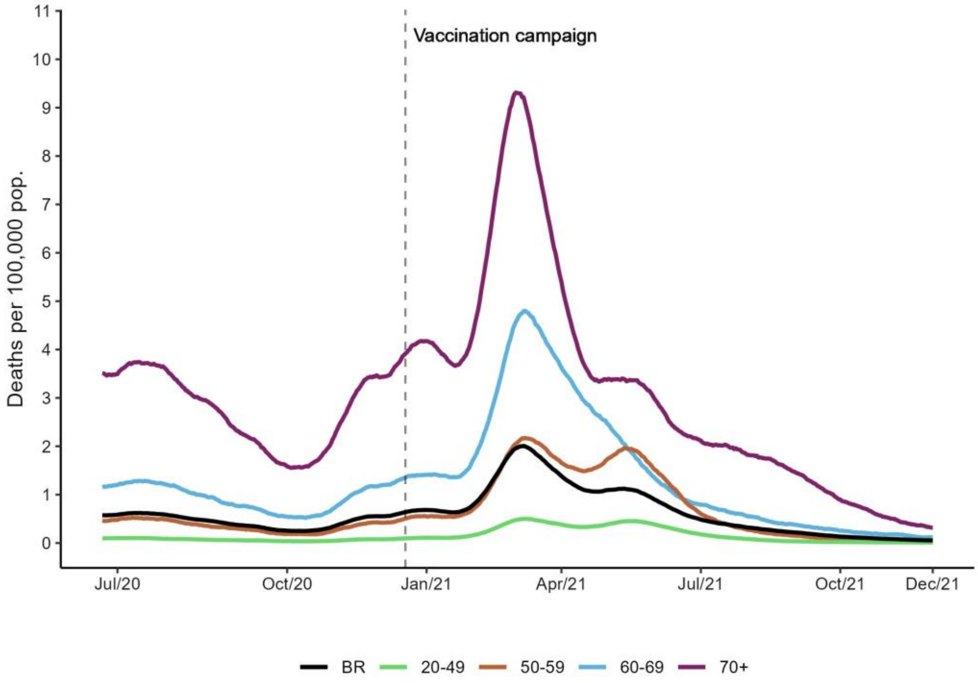

Figure S2. (A) First dose vaccination coverage stratified by age group – first vertical dashed line on the left refers to the start of the vaccination campaign for each age group, the horizontal dashed line indicates the 75% vaccination coverage target by the World Health Organization (WHO). (B) Second or single vaccination coverage stratified by age group.

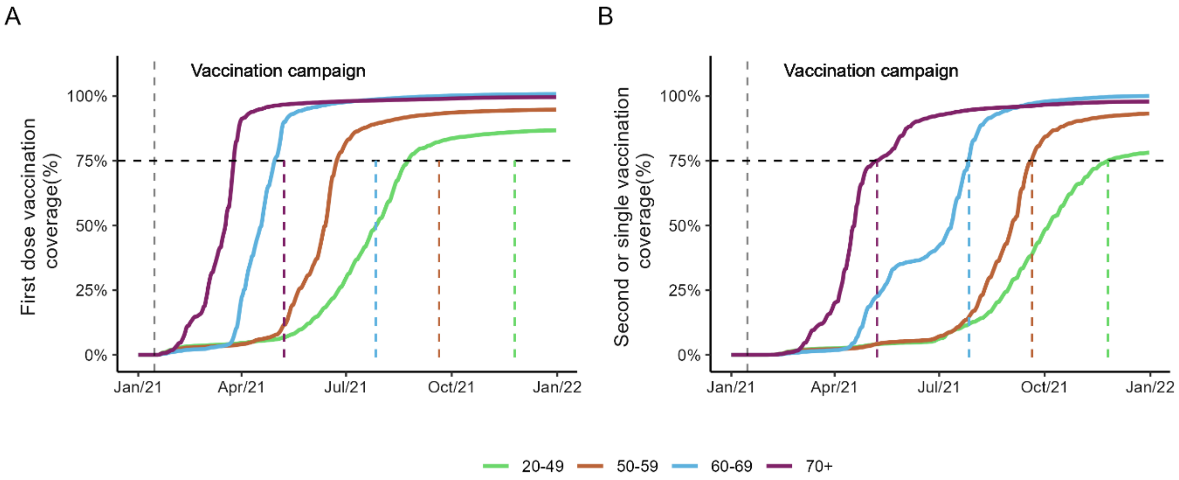

For peer review only - http://bmjopen.bmj.com/site/about/guidelines.xhtml

Table S2. Monthly average of the mortality ratios in Brazil, in 2021, stratified by age group.

Age group	Jan/21	Feb/21	Mar/21	Apr/21	Mai/21	Jun/21	Jul/21	Aug/21	Sep/21	Oct/21	Nov/21	Dec/21
20 – 49	0.15	0.17	0.22	0.26	0.31	0.41	0.41	0.31	0.2	0.16	0.16	0.18
50 – 59	0.79	0.85	0.96	1.14	1.38	1.72	1.53	1.03	0.75	0.63	0.69	0.74
60 – 69	2.12	2.12	2.22	2.48	2.58	1.74	1.52	1.7	1.7	1.81	1.95	2.16
70+	6.16	5.87	5.21	4.29	3.38	3.11	3.69	5.1	6.35	6.77	6.48	5.96

For peer review only

For peer review only - http://bmjopen.bmj.com/site/about/guidelines.xhtml

Table S3. Number of reported deaths and first dose vaccine coverage per month in during the COVID-19 vaccination campaign in Brazil (started in January 17, 2021).

Age group	Monthly Average Baseline*	Baseline*	Jan/21	Feb/21	Mar/21	Apr/21	Mai/21	Jun/21	Jul/21	Aug/21	Sep/21	Oct/21	Nov/21	Dec/21
Monthly reported deaths														
BR	20,627	123,763	29,346	28,141	62,603	79,939	53,772	48,416	31,293	18,485	12,164	8,254	4,958	3,223
20 – 49	2,050	12,298 (10 %)	2,842 (10 %)	3,149 (11 %)	9,099 (15 %)	13,299 (17 %)	10,617 (20 %)	12,620 (26 %)	8,316 (27 %)	3,714 (20 %)	1,626 (13 %)	832 (10 %)	506 (10 %)	372 (12 %)
50 – 59	2,545	15,269 (12 %)	3,641 (12 %)	3,748 (13 %)	9,611 (15 %)	14,224 (18 %)	11,623 (22 %)	13,082 (27 %)	7,677 (25 %)	3,022 (16 %)	1,449 (12 %)	830 (10 %)	531 (11 %)	374 (12 %)
60 – 69	4,731	28,385 (23 %)	6,845 (23 %)	6,588 (23 %)	15,385 (25 %)	21,775 (27 %)	15,319 (28 %)	9,363 (19 %)	5,210 (17 %)	3,471 (19 %)	2,276 (19 %)	1,634 (20 %)	1,063 (21 %)	770 (24 %)
70+	11,302	67,811 (55 %)	16,018 (55 %)	14,656 (52 %)	28,508 (46 %)	30,641 (38 %)	16,213 (30 %)	13,351 (28 %)	10,090 (32 %)	8,278 (45 %)	6,813 (56 %)	4,958 (60 %)	2,858 (58 %)	1,707 (53 %)
Monthly average mortality rates per 100,000 people														
BR		0.44	0.62	0.66	1.33	1.76	1.14	1.06	0.67	0.39	0.27	0.18	0.11	0.07
20 – 49		0.07	0.09	0.12	0.30	0.45	0.35	0.43	0.27	0.12	0.06	0.03	0.02	0.01
50 – 59		0.35	0.49	0.56	1.30	1.99	1.57	1.83	1.04	0.41	0.20	0.11	0.07	0.05
60 – 69		0.92	1.32	1.41	2.97	4.34	2.95	1.87	1.00	0.67	0.45	0.32	0.21	0.15
70+		2.73	3.84	3.89	6.83	7.59	3.88	3.31	2.42	1.98	1.69	1.19	0.71	0.41
Vaccine coverage# (%)														
BR			1.82	4.74	13.75	21.92	32.22	50.82	67.72	84.82	88.32	89.58	90.3	90.67
20 – 49			1.99	3.61	4.56	5.98	12.55	28.85	52.75	78.55	83.55	85.26	86.23	86.72
50 – 59			1.65	3.06	4.08	7.64	31.84	81.34	89.73	92.36	93.57	94.16	94.53	94.75
60 – 69			1.05	2.28	19.58	75.48	95.28	97.61	98.79	99.6	100.09	100.4	100.6	100.72
70+			1.85	18.95	90.35	96.32	97.4	97.99	98.38	98.72	99.07	99.4	99.53	99.61

*Baseline period corresponds to the six-month period (July to December 2020), prior to the start of the vaccination campaign. For monthly reported deaths, the baseline is the sum of the six months deaths.

#At least one dose.

For peer review only

For peer review only - http://bmjopen.bmj.com/site/about/guidelines.xhtml

Table S4. Second or single vaccination coverage during the vaccination roll-out in Brazil, in 2021, stratified by age group.

Age group	Jan/21	Feb/21	Mar/21	Apr/21	Mai/21	Jun/21	Jul/21	Aug/21	Sep/21	Oct/21	Nov/21	Dec/21
Vaccine coverage (%)												
BR	0	1.56	3.86	11.37	15.26	17.78	28.38	42.88	62.88	76.18	82.88	84.55
20 – 49	0	1.7	2.54	3.7	4.65	6.05	12.74	26.04	47.34	66.04	75.76	78.08
50 – 59	0	1.45	2.19	3.4	5.21	6.95	17.05	46.05	83.75	90.42	92.44	93.29
60 – 69	0	0.87	1.79	18.39	35.29	41.64	83.04	94.74	97.78	98.94	99.71	100
70+	0.01	1.67	19.17	72.57	85.27	92.52	94.75	95.68	96.59	97.27	97.69	97.83

For peer review only

For peer review only - http://bmjopen.bmj.com/site/about/guidelines.xhtml

Table S5. Monthly and global estimated effect of the vaccination campaign (DID analysis) with their respective 95% confidence intervals stratified by age group.

Age group	Global	Jan/21	Feb/21	Mar/21	Apr/21	Mai/21	Jun/21	Jul/21	Aug/21	Sep/21	Oct/21	Nov/21	Dec/21
20 – 49	1.75 (1.44 - 2.12)	1.05 (0.95 - 1.17)	1.21 (1.09 - 1.35)	1.56 (1.41 - 1.73)	1.78 (1.61 - 1.97)	2.10 (1.90 - 2.32)	2.75 (2.48 - 3.04)	2.80 (2.53 - 3.10)	2.15 (1.93 - 2.38)	1.44 (1.29 - 1.62)	1.09 (0.97 - 1.24)	1.11 (0.97 - 1.27)	1.25 (1.08 - 1.44)
50 – 59	1.47 (1.21 - 1.78)	1.05 (0.94 - 1.16)	1.12 (1.01 - 1.24)	1.28 (1.16 - 1.42)	1.48 (1.34 - 1.64)	1.79 (1.62 - 1.97)	2.21 (2.00 - 2.44)	2.01 (1.82 - 2.22)	1.36 (1.22 - 1.51)	1.00 (0.90 - 1.12)	0.85 (0.75 - 0.96)	0.90 (0.79 - 1.03)	0.98 (0.84 - 1.13)
60 – 69	1.01 (0.84 - 1.23)	1.00 (0.91 - 1.10)	1.00 (0.91 - 1.11)	1.05 (0.95 - 1.15)	1.15 (1.05 - 1.23)	1.20 (1.09 - 1.27)	0.81 (0.73 - 1.32)	0.70 (0.63 - 0.71)	0.79 (0.72 - 0.88)	0.80 (0.72 - 0.89)	0.85 (0.76 - 0.95)	0.92 (0.82 - 1.03)	1.02 (0.91 - 1.16)
70+	0.73 (0.60 - 0.88)	0.98 (0.89 - 1.08)	0.93 (0.85 - 1.03)	0.81 (0.74 - 0.89)	0.68 (0.62 - 0.75)	0.53 (0.48 - 0.58)	0.48 (0.43 - 0.53)	0.56 (0.51 - 0.62)	0.79 (0.72 - 0.87)	1.00 (0.91 - 1.10)	1.08 (0.98 - 1.19)	1.04 (0.93 - 1.15)	0.95 (0.85 - 1.06)

For peer review only

For peer review only - http://bmjopen.bmj.com/site/about/guidelines.xhtml

STROBE 2007 (v4) Statement—Checklist of items that should be included in reports of cross-sectional studies.

Section/Topic	Item No	Recommendation	Reported on page #
**Title and abstract**	1	(*a*) Indicate the study's design with a commonly used term in the title or the abstract	Title, Page 1
		(*b*) Provide in the abstract an informative and balanced summary of what was done and what was found	Abstract, Methods/Findings, Page 2
**Introduction**			
Background/rationale	2	Explain the scientific background and rationale for the investigation being reported	Page 4
Objectives	3	State specific objectives, including any prespecified hypotheses	Page 4
**Methods**			
Study design	4	Present key elements of study design early in the paper	Methods, Pages 4-8, Table S1
Setting	5	Describe the setting, locations, and relevant dates, including periods of recruitment, exposure, follow-up, and data collection	Pages 4-8
Participants	6	(*a*) *Cross-sectional study*—Give the eligibility criteria, and the sources and methods of selection of participants	Page 4-5
Variables	7	Clearly define all outcomes, exposures, predictors, potential confounders, and effect modifiers. Give diagnostic criteria, if applicable	Pages 6-7, Figure S1
Data sources/ measurement	8*	For each variable of interest, give sources of data and details of methods of assessment (measurement). Describe comparability of assessment methods if there is more than one group	Methods, Study design and Participants, Pages 5-7, Table S1
Bias	9	Describe any efforts to address potential sources of bias	Methods, Pages 6-7
Study size	10	Explain how the study size was arrived at	Methods, Data analysis, Pages 6-7
Quantitative variables	11	Explain how quantitative variables were handled in the analyses. If applicable, describe which groupings were chosen and why	Methods, Data analysis, Pages 6-7, Table S3
Statistical methods	12	(*a*) Describe all statistical methods, including those used to control for confounding	Methods, Data analysis, Pages 6-7
		(*b*) Describe any methods used to examine subgroups and interactions	Methods, Data analysis, Pages 6-7
		(*c*) Explain how missing data were addressed	Methods, Data analysis, Pages 6
		(*d*) *Cross-sectional study*—If applicable, describe analytical methods taking account of sampling strategy	NA
		(*e*) Describe any sensitivity analyses	Methods, Data analysis, Pages 6-7
**Results**			

For peer review only - http://bmjopen.bmj.com/site/about/guidelines.xhtml

Participants	13*	(a) Report numbers of individuals at each stage of study—eg numbers potentially eligible, examined for eligibility, confirmed eligible, included in the study, completing follow-up, and analysed	Results, Page 8
		(b) Give reasons for non-participation at each stage	NA
		(c) Consider use of a flow diagram	NA
Descriptive data	14*	(a) Give characteristics of study participants (eg demographic, clinical, social) and information on exposures and potential confounders	Results, Page 8, Table 1
		(b) Indicate number of participants with missing data for each variable of interest	Results, Page 8, Table 1
		(c) *Cohort study*—Summarise follow-up time (eg, average and total amount)	NA
Outcome data	15*	*Cross-sectional study*—Report numbers of outcome events or summary measures	Results, Pages 8-9
Main results	16	(*a*) Give unadjusted estimates and, if applicable, confounder-adjusted estimates and their precision (eg, 95% confidence interval). Make clear which confounders were adjusted for and why they were included	Results, Pages 8-11, Figure 2-3, Figure S1, Tables S2-S4
		(*b*) Report category boundaries when continuous variables were categorized	Results, Pages 8-11, Table 1
		(*c*) If relevant, consider translating estimates of relative risk into absolute risk for a meaningful time period	NA
Other analyses	17	Report other analyses done—eg analyses of subgroups and interactions, and sensitivity analyses	Results, Pages 9-11, Figure 2, Figure 3,
**Discussion**			
Key results	18	Summarise key results with reference to study objectives	Discussion, Pages 11-12
Limitations	19	Discuss limitations of the study, taking into account sources of potential bias or imprecision. Discuss both direction and magnitude of any potential bias	Discussion, Pages 13-14
Interpretation	20	Give a cautious overall interpretation of results considering objectives, limitations, multiplicity of analyses, results from similar studies, and other relevant evidence	Discussion, Pages 11-14
Generalisability	21	Discuss the generalisability (external validity) of the study results	Discussion, Pages 12-13
**Other information**			
Funding	22	Give the source of funding and the role of the funders for the present study and, if applicable, for the original study on which the present article is based	Page 16

*Give information separately for cases and controls in case-control studies and, if applicable, for exposed and unexposed groups in cohort and cross-sectional studies.

Note: An Explanation and Elaboration article discusses each checklist item and gives methodological background and published examples of transparent reporting. The STROBE checklist is best used in conjunction with this article (freely available on the Web sites of PLoS Medicine at http://www.plosmedicine.org/, Annals of Internal Medicine at http://www.annals.org/, and Epidemiology at http://www.epidem.com/). Information on the STROBE Initiative is available at www.strobe-statement.org.

For peer review only - http://bmjopen.bmj.com/site/about/guidelines.xhtml
For peer review only

BMJ Open

The impact of the first year of COVID-19 vaccination strategy in Brazil: An ecological study

Journal:	*BMJ Open*
Manuscript ID	bmjopen-2023-072314.R2
Article Type:	Original research
Date Submitted by the Author:	24-Dec-2023
Complete List of Authors:	Aguilar, Soraida; Pontifical Catholic University of Rio de Janeiro, Department of Industrial Engineering<br>Bastos, Leonardo S. L.; Pontifical Catholic University of Rio de Janeiro, Department of Industrial Engineering<br>Maçaira, Paula; Pontifical Catholic University of Rio de Janeiro, Department of Industrial Engineering<br>Baião, Fernanda; Pontifical Catholic University of Rio de Janeiro, Department of Industrial Engineering<br>Simões, Paulo; Pontifical Catholic University of Rio de Janeiro, Department of Industrial Engineering<br>Cerbino-Neto, José; Oswaldo Cruz Foundation<br>Ranzani, Otavio; ISGlobal<br>Hamacher, Silvio; Pontifical Catholic University of Rio de Janeiro, Department of Industrial Engineering<br>Bozza, Fernando; D'Or Institute for Research and Education
**Primary Subject Heading**:	Epidemiology
Secondary Subject Heading:	Public health, Infectious diseases, Health policy
Keywords:	COVID-19, Public health < INFECTIOUS DISEASES, EPIDEMIOLOGY

SCHOLARONE™
Manuscripts

I, the Submitting Author has the right to grant and does grant on behalf of all authors of the Work (as defined in the below author licence), an exclusive licence and/or a non-exclusive licence for contributions from authors who are: i) UK Crown employees; ii) where BMJ has agreed a CC-BY licence shall apply, and/or iii) in accordance with the terms applicable for US Federal Government officers or employees acting as part of their official duties; on a worldwide, perpetual, irrevocable, royalty-free basis to BMJ Publishing Group Ltd ("BMJ") its licensees and where the relevant Journal is co-owned by BMJ to the co-owners of the Journal, to publish the Work in this journal and any other BMJ products and to exploit all rights, as set out in our licence.

The Submitting Author accepts and understands that any supply made under these terms is made by BMJ to the Submitting Author unless you are acting as an employee on behalf of your employer or a postgraduate student of an affiliated institution which is paying any applicable article publishing charge ("APC") for Open Access articles. Where the Submitting Author wishes to make the Work available on an Open Access basis (and intends to pay the relevant APC), the terms of reuse of such Open Access shall be governed by a Creative Commons licence – details of these licences and which Creative Commons licence will apply to this Work are set out in our licence referred to above.

Other than as permitted in any relevant BMJ Author's Self Archiving Policies, I confirm this Work has not been accepted for publication elsewhere, is not being considered for publication elsewhere and does not duplicate material already published. I confirm all authors consent to publication of this Work and authorise the granting of this licence.

For peer review only

The impact of the first year of COVID-19 vaccination strategy in Brazil: An ecological study

Soraida Aguilar, PhD[1]; Leonardo S. L. Bastos, PhD[1,2]; Paula Maçaira, PhD[1]; Fernanda Baião, PhD[1]; Paulo Simões, MSc[1]; José Cerbino-Neto[3], MD, PhD; Otavio T. Ranzani, MD, PhD[4,5]; Silvio Hamacher, PhD[1,2]; Fernando A. Bozza, MD, PhD[3,6,*]

1. Department of Industrial Engineering (DEI), Pontifical Catholic University of Rio de Janeiro (PUC-Rio), Rio de Janeiro, RJ, 22451-900, Brazil

2. Tecgraf Institute, Pontifical Catholic University of Rio de Janeiro (PUC-Rio), Rio de Janeiro, RJ, 22451-900, Brazil

3. National Institute of Infectious Disease Evandro Chagas (INI), Oswaldo Cruz Foundation (FIOCRUZ), Rio de Janeiro, RJ, 21045-900, Brazil

4. Barcelona Institute for Global Health, ISGlobal, Barcelona, 08003, Spain.

5. Pulmonary Division, Heart Institute (InCor), Hospital das Clinicas HCFMUSP, Faculdade de Medicina da Universidade de São Paulo, São Paulo, Brazil

6. D'Or Institute for Research and Education (IDOR), Rio de Janeiro, RJ, 22281-100, Brazil

Corresponding author:

***Fernando A. Bozza, MD, PhD.,** Laboratório de Pesquisa Clínica em Medicina Intensiva, Instituto Nacional de Infectologia Evandro Chagas (INI), Fundação Oswaldo Cruz (FIOCRUZ), Av. Brasil 4365, Rio De Janeiro, RJ, 21045-900, Brazil.

Email: fernando.bozza@ini.fiocruz.br or bozza.fernando@gmail.com

For peer review only

For peer review only - http://bmjopen.bmj.com/site/about/guidelines.xhtml

Abstract

Objectives: No consensus exists about the best COVID-19 vaccination strategy to be adopted by low- and middle-income countries. Brazil adopted an age-based calendar strategy to reduce mortality and the burden on the healthcare system. This study evaluates the impact of the vaccination campaign in Brazil on the progression of the reported COVID-19 deaths.

Methods: This ecological study analyses the dynamic of vaccination coverage and COVID-19 deaths in hospitalised adults (≥20 years) during the first year of the COVID-19 vaccination roll-out (January-December, 2021) using nationwide data (DATASUS). We stratified the adult population into 20-49, 50-59, 60-69, and 70+ years. The dynamic effect of the vaccination campaign on mortality rates was estimated by applying a Negative Binomial Regression. The prevented and possible preventable deaths (observed deaths higher than expected) and Potential Years of Life Lost (PYLL) for each age group were obtained in a counterfactual analysis.

Results: During the first year of COVID-19 vaccination, 266,153,517 doses were administered, achieving 91% first-dose coverage. A total of 380,594 deaths were reported, 154,091 (40%) in 70+ years and 136,804 (36%) from 50-59 or 20-49 years. The mortality rates of 70+ decreased by 52% (RR [95% CI]: 0.48 [0.43-0.53]) in 6 months, whereas rates for 20-49 were still increasing due to low coverage (52%). The vaccination roll-out strategy prevented 59,618 deaths, 53,088 (89%) from those aged 70+ years. However, the strategy did not prevent 54,797 deaths, 85% from those under 60 years, being 26,344 (45%) only in 20-49, corresponding to 1,589,271 PYLL, being 1,080,104 PYLL (68%) from those aged 20-49 years.

Conclusion: The adopted aged-based calendar vaccination strategy initially reduced mortality in the oldest but did not prevent deaths of the youngest as effectively as compared to the older age group. Countries with a high burden, limited vaccine supply and young populations should consider other factors beyond age to prioritise who should be vaccinated first.

For peer review only - http://bmjopen.bmj.com/site/about/guidelines.xhtml

Strengths and limitations of this study

- We evaluated the impact of the COVID-19 vaccination strategy in Brazil on the number of reported deaths during the campaign's first year. In 2021, 266,153,517 COVID-19 vaccine doses were administered in adults with 91% vaccination coverage.
- The vaccination strategy followed an age calendar and prioritised the high-risk groups, and following an age-based calendar, the mortality rates of the elderly population decreased earlier than those of the young population.
- Alternative strategies to an age-based calendar are necessary for countries with low-resource and young populations. These strategies should consider the local burden, vaccination supply and speed, vaccine hesitancy, and socioeconomic vulnerability.
- Non-pharmacological Interventions (NPIs) that may affect the progression of deaths and mortality rates were not considered in the analysis.
- This ecological study analysed notified data in the SIVEP-Gripe, which limits the assessment of all COVID-19 deaths and excess mortality in the country.

For peer review only

For peer review only - http://bmjopen.bmj.com/site/about/guidelines.xhtml

Introduction

The COVID-19 pandemic imposed unprecedented challenges worldwide. In 2020, the pandemic in Brazil quickly spread across the different states, with distinct patterns and burdens due to the disparities in health assistance, income and a lack of a unified national strategy to face the pandemic.[1,2] With the emergence and dominance of SARS-CoV-2-Variants of Concern (VOC)[3] at the end of 2020,[4] characterized by high transmissibility,[4] immune escape,[5,6] and increased disease severity, substantial pressure was imposed on the health system. At the beginning of 2021, when the vaccination campaign started in the country, a new surge – the second COVID-19 Gamma wave – was still rampant, with higher mortality than the first.[3]

Countries have used different strategies to define who vaccinates first.[7,8] Several studies evaluated the impact of vaccination campaigns on infections, hospitalizations and saving lives principally in high-income countries.[8–11] In Brazil, the National Immunization Program (PNI), responsible for providing the vaccination guidelines, is part of the Brazilian universal health system (*Sistema Unico de Saúde – SUS*) and has been recognized as a model of vaccination campaigns and population adhesion worldwide.[12] The Brazilian COVID-19 vaccination campaign, which started on January 17, 2021, prioritised high-risk groups and considered an age-based calendar beginning with the elderly (85 years or more).[13] Vaccine distribution accomplished the national guidelines centralizing the distribution of vaccine doses to the states and municipalities.

It is still unclear how to best prioritise groups for mass vaccination during pandemics/outbreaks such as COVID-19, especially in low- and middle-income countries (LMICs) with a high burden of the disease and a young population.[14–16] Hence, we performed an ecological study to evaluate the impact of the vaccination campaign in Brazil on the progression of the reported COVID-19 deaths. We estimated the number of prevented and possible preventable deaths during the campaign stratified by age groups.

Methods

Study Design and Participants

We conducted an ecological study to analyse the progression of reported COVID-19 deaths and mortality rates before and during the first year of the vaccination campaign roll-out in Brazil.

Daily data on reported deaths were obtained from the Influenza Epidemiological Surveillance Information System (*Sistema de Informação de Vigilância Epidemiológica da Gripe*, SIVEP-Gripe), a nationwide surveillance open-access database used to monitor severe acute

For peer review only

For peer review only - http://bmjopen.bmj.com/site/about/guidelines.xhtml

respiratory infections (SARI) in Brazil. SIVEP-Gripe has been the primary source of reported COVID-19 deaths in the country. This database covers all Brazilian municipalities; however, in-hospital people belong to 80% of these municipalities, where those territories correspond to 96% of Brazil's population.[2] Each register contains information about the individual's demographics, self-reported symptoms, comorbidities, hospital and ICU admission and ventilatory support, in-hospital outcome (death or discharge), and dates of symptom onset, hospital admission, and ICU admission. We included all adult participants (≥20 years old) with SARS-CoV-2-confirmed infection (RT-PCR or clinical-epidemiological criteria) whose death was reported between February 27, 2020, and December 31, 2021.

Daily data on COVID-19 vaccination coverage were obtained from the Brazilian National Immunization Program database (SI-PNI). The SI-PNI contains information on the individual's demographics (age, sex, and place of residence), vaccine dose and platform, and place and date of dose administration. We included all doses (first, second or single) administered to adults (≥20 years old) between January 17, 2021 (the start of the vaccination campaign) and December 31, 2021. Finally, we used the 2020 population estimates provided by the Brazilian Ministry of Health stratified by age.

Data were publicly available, anonymized, and de-identified. Following ethically agreed principles on open data, this analysis did not require ethical approval in Brazil. Information on the data sources is shown in online supplemental table S1.

Patient and public involvement

No patient was involved.

COVID-19 Vaccination Campaign Roll-Out in Brazil

On January 17, 2021, the Brazilian national immunization program (PNI) initiated the vaccination campaign for COVID-19. Vaccine doses were freely distributed to states and municipalities, which managed the administration according to the availability of resources. The COVID-19 vaccination campaign strategy followed a national age-based calendar during the whole period, with some local adaptations. It started in parallel vaccinating those aged 90+, indigenous individuals, healthcare workers, and high-risk groups based on comorbidities and then followed an exclusively age-based calendar. Four vaccine platforms were considered in the country: two

For peer review only - http://bmjopen.bmj.com/site/about/guidelines.xhtml

For peer review only

doses of Sinovac-CoronaVac, ChAdOx1-S/nCoV-19 (Oxford-AstraZeneca), or BNT162b2 (Pfizer/BioNTech), or Ad26.COV2.S (Janssen) single-dose.

Outcomes

Our primary outcome was the estimated number of prevented COVID-19 deaths. Secondary outcomes comprise possible preventable deaths, the potential years of life lost (PYLL), the mortality rates per 100,000 population, and the first dose and second or single dose vaccination coverage. All quantities, rates, and estimates were evaluated temporally for the whole country and stratified by age groups; no further stratification was performed.

Data Analysis

We described continuous variables using medians and interquartile ranges (IQRs) or means and standard deviations (SDs) when applicable and frequencies and proportions for categorical variables. The analysis was pre-specified, and the sample size was pragmatic (SIVEP-Gripe database). All analyses considered complete data, and no imputation of missing values was performed.

We evaluated the progression of COVID-19 deaths before and during the vaccination campaign (February 26, 2020, to December 31, 2021) for the whole country and stratified by age groups. We generated the time series of daily reported deaths and mortality rates using the 15-day moving average to reduce the effects of variability in the notification process. Changes in the representativeness of age groups in the deaths were assessed by calculating the proportion of each group in the daily count. In addition, vaccination coverage of at least one dose ("partially vaccinated") and two or single doses ("fully vaccinated") was calculated as the number of doses divided by the corresponding estimated population.

To estimate the impact of the vaccination campaign strategy on the evolution of daily reported mortality rates for each age group (20-49, 50-59, 60-69, and 70+), we performed a three-stage analysis.

First, we estimated the dynamic effects of the vaccination campaign to examines how age-specific death rates deviated from the national average before and after the onset of the vaccination campaign. The national average is represented by the national age-adjusted mortality COVID-19 rates in Brazil as a surrogate of the pandemic progression in the country

For peer review only - http://bmjopen.bmj.com/site/about/guidelines.xhtml

during the vaccination campaign. We compared the daily mortality rates of each age group and the national age-adjusted mortality COVID-19 rates using Rate Ratios (RR). Afterwards, we applied a Negative Binomial Regression model. The response variable (y_t) was the monthly COVID-19 reported deaths, and the covariates were the age group ($x_{age,t}$), the period indicator ($x_{period,t}$), separated into indicators, one for each month, their interaction ($x_{age,t} * x_{period,t}$), and the population ($x_{population}$) as the offset variable. To obtain the dynamic effects for each age group, we used the national average as the reference. The effect was calculated as the exponentiated coefficient of the interaction terms, defined as the Rate Ratio (RR) and their corresponding 95% confidence intervals. The Negative Binomial Regression model is represented as follows:

$$\ln y_t = \beta_0 + \beta_1 x_{age,t} + \beta_2 x_{period,t} + \beta_3 x_{age,t} * x_{period,t} + offset(x_{population}) \quad (1)$$

where t represents the monthly observations and β_0, β_1, β_2 and β_3 are regression coefficients. Additionally, a sensitivity analysis is performed by exchanging the national average as reference to the older and younger (70+ and 20-49) age groups.

Subsequently, we quantified the impact of the vaccination strategy in terms of prevented and possible preventable deaths for each age group using a counterfactual analysis.[17] Using a Linear Regression model, we predicted the mortality rate of an age group (response variable, y_i) based on the national age-adjusted mortality rates in the country (predictor variable, x_i).[17] The model is expressed by:

$$y_i = \beta_0 + \beta_1 x_i + \varepsilon_i \quad (2)$$

where ε_i denotes the error term, i is the daily observations of the target age group and β_0 and β_1 are regression coefficients. Although the national age-adjusted mortality rates in the country do not strictly represent a scenario without vaccination, we could obtain the number of expected deaths considering this progression as being the dynamic pandemic progression and the vaccination roll-out as the speed and effectiveness in the decrease of deaths for each age group.

We considered the start of the vaccination roll-out as the date of intervention onset (January 17, 2021). Hence, we fit the model using data prior to the vaccination roll-out to obtain the of predicted mortality rates for each age group in the following period. We multiplied the predicted mortality rates by the total population to obtain the number of expected deaths.

For peer review only - http://bmjopen.bmj.com/site/about/guidelines.xhtml

When comparing the number of expected to observed deaths for each age group, we considered "prevented deaths" as when the observed deaths were lower than the expected deaths; otherwise, we considered "possible preventable deaths".

Furthermore, we estimated the corresponding number of prevented and preventable Potential Years of Life Lost (PYLL) for each age group, considering the midpoint of each age group and a life expectancy of 76 years for Brazil in 2019.[18] All analyses were performed in R 4.2.1. We followed STROBE recommendations.

Role of the Funding Source

The funders had no role in any decision about the manuscript. All authors had full access to all the data in the study. SA, PM, FB, and LSLB verified the data, and all authors approved the final version of the manuscript for publication.

Results

Age-Adjusted Mortality and Vaccine Coverage in the First Year of Vaccine Roll-Out

From February 26, 2020, to December 31, 2021, 565,774 COVID-19 deaths were reported (**figure 1-A**). Most of these comprised those aged 70 years or older (253,092, 45%), and one-third were those aged under 60 years (**figure 1-B**). The daily average mortality rates per 100,000 population were the highest between March and Mai 2021 for all most the age groups and occurred during the dominance of VOC Gamma (**online supplemental figure S1-A and table S2**). When comparing the proportion of reported deaths (**figure 1-C**), the 70+ age group showed a decrease since March 2021, two months after the vaccination roll-out start, being the lowest in May-June 2021, almost 50% lower compared to pre-vaccination period. During this period, the proportion of deaths related to 20-49 and 50-59 years increased. However, in late 2021, there was a gradual reduction in the proportion of deaths related to those under 60 years.

With respect to the vaccination roll-out, 266,153,517 vaccine doses for COVID-19 were administered in adults (≥20 years old) in 2021, with 137,644,737 (51.66%) first doses and 128,508,780 (48.34%) second or single doses. At the end of 2021, the national first dose vaccination coverage for adults was 90.7%, with 86.74% for 20-49 years, 94.75% for 50-59 years, 100.78% for 60-69 years, and 99.59% for 70+ years (**online supplemental tables S3 and S4**). The progression of vaccination coverage rates was different for each age group due to the campaign

For peer review only - http://bmjopen.bmj.com/site/about/guidelines.xhtml

strategy (**figure 1-D**). Brazil achieved 75% vaccination coverage in August 2021, six months after the vaccination roll-out. In the same period, the 70+ group had 98% first-dose coverage, whereas the 20-49 group presented 52% coverage (**online supplemental table S3**). We observed that the oldest group (70+) already had more than 90% vaccination coverage by the end of March 2021. Similar behaviour for the second or single dose was observed (**online supplemental figure S2 and table S4**).

Vaccination Effect on Mortality Rates

To assess the impact of the vaccination campaign strategy on reducing mortality rates, we compared the mortality rates for each age group with the national age-adjusted mortality rates in the country (**figure 2-A and online supplemental figure S1-A**). The 70+ group showed the highest mortality rates in the whole period (Rate Ratio > 1). However, six months later the vaccination roll-out started, the mortality rates reduced from 3.84 deaths per 100,000 population (Rate Ratio: 6.16) in January 2021 to 3.31 deaths per 100,000 (Rate Ratio: 3.11) in June 2021 (**online supplemental table S2**). In the same period, the 20-49 age group showed an increase in mortality rates (Rate Ratio: 0.21 in January 2021 and 0.58 in June 2021) compared to the national age-adjusted mortality rates. After September 2021, the Rate Ratios for all age groups returned to similar levels to those seen before vaccination (**online supplemental table S2**).

We estimated the dynamic effect of age-specific death rates and how they deviated from the national average using a Negative Binomial Regression model to evaluate the association between the reported deaths, age group and the period indicator (**figure 2-B**). When analysing the temporal progression of the RR stratified by age group (**online supplemental table S5**), the 70+ age group had the lowest rates in June 2021 (RR [95% CI]: 0.48 [0.43-0.53]), which evidenced a 52% decrease in the number of deaths in 5 months. With regard to age groups 50-59 and 60-69, we estimated a decrease in death rates of 15% and 30% (RR [95% CI]: 0.85 [0.75-0.96] and RR [95% CI]: 0.70 [0.63-0.71], respectively) for at least 4 and 6 months in the first year of vaccination (**online supplemental table S5**).

When performing the sensitive analysis (**online supplemental figure S3 and tables S6-S7**), the extreme age groups (20-49 and 70+) display a similar behaviour from those age groups obtained from the dynamic effect when comparing to the national average (**figure 2-B**). Furthermore, evaluating the before and after the vaccination campaign (reference Dec/20) was evidenced a rise on the mortality rates (**online supplemental figure S1-B and table S8**) after the

For peer review only - http://bmjopen.bmj.com/site/about/guidelines.xhtml

start of the vaccination campaign which became increasingly higher for younger age groups (20-49 and 50-59). In June there was a second increase that was not repeated for the older age groups (60-69 and 70+).

Number of Prevented and Possible Preventable Deaths and Potential Years of Life Lost (PYLL)

We quantified the number of prevented and possible preventable deaths in each age group during the vaccination roll-out (**figure 3**). We estimated that vaccination prevented 59,618 deaths compared to the expected deaths in the first year of vaccination roll-out (**table 1**). Of these, 89% were prevented in those aged 70 years or more (53,088), followed by those aged 60-69 years (6,032, 10%), which achieved high vaccination coverage earlier than younger age groups (**figure 1-D**).

Table 1. Comparison between observed and predicted deaths during the vaccination campaign in Brazil in 2021, stratified by age group.

Results	Age group				Total
	20-49	50-59	60-69	70+	
Observed deaths	65,582	67,991	86,126	145,812	365,511
Expected deaths	39,393	48,098	85,064	197,777	370,332
Prevented deaths	155	343	6,032	53,088	59,618
Possible preventable deaths	26,344	20,236	7,094	1,123	54,797
Prevented PYLL[#] †	6,355	7,203	66,352	291,984	371,894
Preventable PYLL[#] †	1,080,104	424,956	78,034	6,177	1,589,271

Prevented deaths were obtained if observed deaths were lower than predicted, and possible preventable deaths, were obtained if the number of observed deaths was higher than predicted.
†The denominator is the adult population of Brazil for this age group = 151,778,738.
[#] PYLL was calculated using the average age for each group and a life expectancy of 76 years for Brazil in 2020.

We considered possible preventable deaths when the number of observed deaths was higher than those expected if each age group followed the progression of the national age-adjusted mortality rates in the country (**figure 3, table 1**). Despite the considerable number of deaths prevented, we estimated 54,797 potentially preventable deaths in the same period. More than 85% of possible preventable deaths comprised those under 60 years old, half of them corresponding to those aged 20-49 years (26,344, 48%), which achieved late vaccination coverage compared to older groups (**figure 1-D**).

For peer review only - http://bmjopen.bmj.com/site/about/guidelines.xhtml

In addition, we estimated the impact of the vaccination roll-out in terms of the Potential Years of Life Lost (PYLL). The vaccination strategy prevented 371,894 PYLL, especially for those aged 60 years or older. However, it was not able to prevent 1,589,271 PYLL, composed mainly of those aged 20-49 years (68%) (**table 1**).

Discussion

We evaluated the impact of the COVID-19 vaccination strategy in Brazil on the number of reported deaths during the campaign's first year by analysing the progression of vaccination coverage and reported deaths and model the prevented and possible preventable deaths. In prioritising the vaccination of high-risk groups, the mortality rates of the elderly population reduced earlier compared to the young population, especially in a period of high burden due to the introduction of a new VOCs. After 6 months of vaccination roll-out, the vaccination coverage for those aged 70+ years was 98% compared to only 52% for the age group 20-49, which resulted in a reduction of 44%, and the mortality rates in the 70+ years and for those aged 20-49 were still increasing due to the low coverage. At the end of 2021, the first dose coverage achieved 91%, and the estimated number of prevented deaths was 59,618. However, the delay in the vaccination of younger ages may have resulted in an expressive number of deaths that could be prevented in this group.

Brazil was one of the epicentres of COVID-19 deaths in 2020, which intensified in a second and larger surge at the beginning of 2021 when the COVID-19 vaccination campaign started.[3] The first months comprised limited vaccine dose availability until May 2021. The strategy prioritised the immunization of high-risk groups (i.e., indigenous, healthcare workers, and comorbidities) and older ages, intending to reduce the burden of deaths and overload over the health system. This strategy resulted in a rapid decrease in mortality rates in the country, driven mainly by those aged 70+, which displayed the highest number of prevented deaths (53,088) in the period. The strategy successfully prevented deaths in the older population; however, the major drawback was the expressive number of deaths in those aged < 60 years old. Although non-pharmacological interventions (NPIs) such as social distancing and masks mandates have been implemented in Brazil, there was not a coordinated and unified guideline at the national level. Due to the country heterogeneity, municipalities applied NPIs under different flags, but not with a high degree of adherence; despite this when the vaccination campaign began none NPIs continued.

For peer review only - http://bmjopen.bmj.com/site/about/guidelines.xhtml

Except for those with high-risk conditions, vaccination for younger groups started only four to six months after vaccination for older groups, during the third surge of deaths (July 2021). Although mortality risks for this population are lower compared to the elderly, Brazil is composed mainly of young people. At the end of 2021, 86,834 deaths were reported for the 20-49 age group, where 66,692 (77%) occurred during the vaccination roll-out period. The trend of mortality for the youngers started to decrease in July 2021 and was more pronounced in September 2021, when vaccination coverage of this population was higher than 75%. Our analysis did not discriminate between prioritised high-risk groups from those individuals that following the age-based calendar in the vaccination campaign, since this is a low percentage of doses from these groups, especially those based on comorbidities lower than 10%, hence not significantly impacting the results or the progression of the vaccination campaign.

We estimated that the vaccination strategy prevented 59,618 deaths, composed mainly of those aged 60-69 or 70+ years. However, 54,797 possible preventable deaths of those aged 50-59 or 20-49 years occurred, corresponding to a potential savings of 1,589,271 PYLL, 68% from 20-49 years. These results align with previous studies that identified a reduction in deaths and hospitalizations due to vaccination in high-income countries.[8–11] A recent Brazilian study obtained 303,129 prevented deaths in the period from January 17, 2021 to January 31, 2022, with 61% from those aged >= 60 and 7% for the 20-39 age group.[19] Our estimates were obtained using a counterfactual analysis in which the control group represented the progression of national age-adjusted mortality rates during the vaccination campaign. Although it does not represent a scenario strictly without vaccination, we could obtain the number of expected deaths considering the dynamics in pandemic progression and the vaccination roll-out as the speed and effectiveness in the decrease of deaths for each age group. Hence, we were able to evaluate the results from the age-based calendar strategy and quantify the number of prevented and possible preventable deaths for each age group during the first year of the vaccination campaign.

Latin America was highly affected by the pandemic, the continent with the highest number of deaths per capita, with limited access to vaccine doses.[20,21] In Brazil, the availability of vaccine doses at the beginning of the vaccination roll-out was low due to the national government's late negotiation for vaccine purchases, global demand, and the high concentration of doses in rich countries.[13,22] The concern with respect to vaccine hesitancy, encouraged by the country's leadership and polarized political environment, was not confirmed by previous evidence and high vaccination coverage was achieved.[13,22–24] The urgency of

For peer review only - http://bmjopen.bmj.com/site/about/guidelines.xhtml

For peer review only

vaccination also refers to better use of a common resource, including access to vaccines, prioritising care for those most affected, i.e., where the epidemic is behaving most intensely.[25]

Recently, the WHO revised the initial recommendation of prioritising the years of life saved to reduce deaths and disease burden, especially in scenarios of limited access to vaccines.[25] In Brazil, due to the low availability of vaccine doses at the start of the roll-out, a strategy that followed an age-based calendar also prioritising high-risk groups was adopted. Our results demonstrated that the strategy protected the oldest. However, it could not significantly prevent the deaths of the youngest. These findings raise concerns about whether the age-based calendar is the best strategy for countries with low-resource settings and a young population[26]. Should alternative strategies prioritise locals with high burden and increase the years of life saved in locations with limited healthcare resources and socioeconomic vulnerability? Alternative strategies to the age-based calendar have been proposed pointing out the importance of considering the burden, age distribution, vaccination supply and speed, hesitancy, and socioeconomic vulnerability to promote a better and more equitable decision-making process.[14,15,25,27,28] Other model approaches, such as agent-based or deterministic compartmental models, may also assess the potential gains of alternative strategies.[15,16]

The findings of this study should be interpreted in light of some potential limitations. First, we analysed data notified in the SIVEP-Gripe, which consists of cases reported by the surveillance system. This limits the assessment of all COVID-19 deaths and excess mortality in the country. However, the SIVEP-Gripe has been one of the country's central repositories of COVID-19 epidemiological data, with high information coverage[2]. Second, stringent policies and actions, as well as personal behaviour risk, may affect the progression of deaths and mortality rates and were not considered in the analysis. Third, although the vaccination roll-out started at the same time across the country, regional differences can indeed impact the vaccine coverage speed due to limited access or the age composition of certain locations.[13] Fourth, we did not take into account for previous infection, once the elderly had the highest rate of infection early in the pandemic, the natural immunity in this group might have also contributed to the same protection observed against death. Furthermore, the study has strengths: this is a nationwide data analysis of a large LMIC with a high burden of COVID-19; we estimate the dynamic impact of the vaccination program in four age groups considering an average behaviour, which allows the assessment and understanding of the vaccination roll-out within the country.

For peer review only - http://bmjopen.bmj.com/site/about/guidelines.xhtml

Conclusion

Our findings indicate that the vaccination campaign was able to prevent more than 59,000 deaths in Brazil, especially for the older population. However, the delay in the vaccination of younger ages may have resulted in an expressive number of deaths that could be prevented in this group.

For peer review only

For peer review only - http://bmjopen.bmj.com/site/about/guidelines.xhtml

Data availability statement

Data are available on reasonable request.

Ethics statements

Patient consent for publication

Not applicable.

Acknowledgements

This work is part of the Grand Challenges ICODA pilot initiative, delivered by Health Data Research UK and funded by the Bill & Melinda Gates Foundation and the Minderoo Foundation. This study was also supported by the National Council for Scientific and Technological Development (CNPq), the Coordination for the Improvement of Higher Education Personnel (CAPES) - Finance Code 001, Carlos Chagas Filho Foundation for Research Support of the State of Rio de Janeiro (FAPERJ), the Pontifical Catholic University of Rio de Janeiro. OTR is funded by a Sara Borrell grant from the Instituto de Salud Carlos III (CD19/00110). OTR acknowledges support from the Spanish Ministry of Science and Innovation and State Research Agency through the "Centro de Excelencia Severo Ochoa 2019-2023" Program (CEX2018-000806-S) and support from the Generalitat de Catalunya through the CERCA Program.

We are grateful to all healthcare workers for their endless efforts to tackle the COVID-19 pandemic in Brazil. We thank the NOIS research group and the ICODA driver project EFFECT-Brazil team members for the discussions and collaborative production during the COVID-19 pandemic. All authors carried out the research independently of the funding body. The findings and conclusions in this manuscript reflect the opinions of the authors alone.

For peer review only - http://bmjopen.bmj.com/site/about/guidelines.xhtml

Contributors

SA, LSLB, PM, FB, PS, JCN, OTR, SH and FAB participated in the design and concept of the study. SA, LSLB and PM did the data curation. SA, LSBL, PM, FB, OTR and FAB designed the data analysis. SA, LSLB, PM, FB, PS, SH and FAB performed the data analysis. SA, LSLB, PM, FB and FAB wrote the first version of the manuscript. LSLB, PM, FB, SH and FAB supervised the study. All authors had full access to the data, participated in data interpretation, revised the manuscript, and approved the final version of the manuscript.

Funding

This work is part of the Grand Challenges ICODA pilot initiative, delivered by Health Data Research UK and funded by the Bill & Melinda Gates Foundation (INV-017293) and the Minderoo Foundation. This study was supported by the National Council for Scientific and Technological Development (CNPq), the Coordination for the Improvement of Higher Education Personnel (CAPES) - Finance Code 001, Carlos Chagas Filho Foundation for Research Support of the State of Rio de Janeiro (FAPERJ) and the Pontifical Catholic University of Rio de Janeiro. OTR is funded by a Sara Borrell grant from the Instituto de Salud Carlos III (CD19/00110). OTR acknowledges support from the Spanish Ministry of Science and Innovation and State Research Agency through the "Centro de Excelencia Severo Ochoa 2019-2023" Program (CEX2018-000806-S), and support from the Generalitat de Catalunya through the CERCA Program.

All authors carried out the research independently of the funding bodies. The findings and conclusions in this manuscript reflect the opinions of the authors alone.

Competing interests

SH and FAB are funded by the CNPq and FAPERJ. PM is funded by CNPq (422470/2021-0) and FAPERJ (E-26/211.645/2021 and E-26/201.348/2022). OTR is funded by a Sara Borrell fellowship from the Instituto de Salud Carlos III (CD19/00110), acknowledges support from the Spanish Ministry of Science and Innovation and State Research Agency through the “Centro de Excelencia Severo Ochoa 2019–2023” Program (CEX2018-000806-S), support from the Generalitat de Catalunya through the CERCA Program, and received a research grant from the Health Effects Institute for research unrelated to this manuscript. OTR was also member of the Data Safety Monitoring Board in the REVOLUTION and STOP-COVID trials, testing treatments

For peer review only - http://bmjopen.bmj.com/site/about/guidelines.xhtml

against COVID-19, and is currently member of the Data Safety Monitoring Board of the RENOVATE trial, testing respiratory support strategies in patients with acute respiratory hypoxaemic failure. The other authors declare that they have no conflicts of interest.

All authors carried out the research independently of the funding bodies. The findings and conclusions in this manuscript reflect the opinions of the authors alone.

Data sharing

All data, including individual participant data, is publicly available with de-identification and anonymisation of patients. The data sources are described in the manuscript and in the supplementary material (**online supplemental table S1**). The raw data and code used for the analysis are available in a GitHub repository, with publication (https://github.com/noispuc/ICODA_COVID_VaccineStrategy)

For peer review only - http://bmjopen.bmj.com/site/about/guidelines.xhtml

References

1 Castro MC, Kim S, Barberia L, *et al.* Spatiotemporal pattern of COVID-19 spread in Brazil. *Science (80-)* 2021;**372**:821–6. doi:10.1126/science.abh1558

2 Ranzani OT, Bastos LSL, Gelli JGM, *et al.* Characterisation of the first 250 000 hospital admissions for COVID-19 in Brazil: a retrospective analysis of nationwide data. *Lancet Respir Med* 2021;**9**:407–18. doi:10.1016/S2213-2600(20)30560-9

3 Bastos LS, Ranzani OT, Souza TML, *et al.* COVID-19 hospital admissions: Brazil's first and second waves compared. *Lancet Respir Med* 2021;**9**:e82–3. doi:10.1016/S2213-2600(21)00287-3

4 Faria NR, Mellan TA, Whittaker C, *et al.* Genomics and epidemiology of the P.1 SARS-CoV-2 lineage in Manaus, Brazil. *Science (80-)* 2021;**372**:815–21. doi:10.1126/science.abh2644

5 Sabino EC, Buss LF, Carvalho MPS, *et al.* Resurgence of COVID-19 in Manaus, Brazil, despite high seroprevalence. *Lancet* 2021;**397**:452–5. doi:https://doi.org/10.1016/S0140-6736(21)00183-5

6 Planas D, Saunders N, Maes P, *et al.* Considerable escape of SARS-CoV-2 Omicron to antibody neutralization. *Nature* 2022;**602**:671–5. doi:10.1038/s41586-021-04389-z

7 Voigt A, Omholt S, Almaas E. Comparing the impact of vaccination strategies on the spread of COVID-19, including a novel household-targeted vaccination strategy. *PLoS One* 2022;**17**:1–16. doi:10.1371/journal.pone.0263155

8 Kim D, Lee YJ. Vaccination strategies and transmission of COVID-19: Evidence across advanced countries. *J Health Econ* 2022;**82**:102589. doi:10.1016/j.jhealeco.2022.102589

9 Shoukat A, Vilches TN, Moghadas SM, *et al.* Lives saved and hospitalizations averted by COVID-19 vaccination in New York City: a modeling study. *Lancet Reg Heal - Am* 2022;**5**:100085. doi:10.1016/j.lana.2021.100085

10 Steele MK, Couture A, Reed C, *et al.* Estimated Number of COVID-19 Infections, Hospitalizations, and Deaths Prevented among Vaccinated Persons in the US, December 2020 to September 2021. *JAMA Netw Open* 2022;**5**:E2220385. doi:10.1001/jamanetworkopen.2022.20385

11 Ferreira LS, Marquitti FMD, Silva RLP da, *et al.* Estimating the impact of implementation and timing of COVID-19 vaccination program in Brazil: a counterfactual analysis. *medRxiv* 2021;:2021.12.24.21268384.https://www.medrxiv.org/content/10.1101/2021.12.24.21268384v1%0Ahttps://www.medrxiv.org/content/10.1101/2021.12.24.21268384v1.abstract

12 Peres KC, Buendgens FB, Prates EA, *et al.* Vacinas no Brasil: análise histórica do registro sanitário e a disponibilização no Sistema de Saúde. *Cien Saude Colet* 2021;**26**:5509–22. doi:10.1590/1413-812320212611.13932021

13 Bastos LS, Aguilar S, Rache B, *et al.* Primary healthcare protects vulnerable populations from inequity in COVID-19 vaccination: An ecological analysis of nationwide data from Brazil. *Lancet Reg Heal* 2022;:100335.

14 McIntyre PB, Aggarwal R, Jani I, *et al.* COVID-19 vaccine strategies must focus on severe disease and global equity. *Lancet* 2022;**399**:406–10. doi:10.1016/S0140-6736(21)02835-X

15 Saadi N, Chi YL, Ghosh S, *et al.* Models of COVID-19 vaccine prioritisation: a systematic

For peer review only - http://bmjopen.bmj.com/site/about/guidelines.xhtml

literature search and narrative review. *BMC Med* 2021;**19**:1–11. doi:10.1186/s12916-021-02190-3

16 Liu Y, Sandmann FG, Barnard RC, *et al.* Optimising health and economic impacts of COVID-19 vaccine prioritisation strategies in the WHO European Region: a mathematical modelling study. *Lancet Reg Heal - Eur* 2022;**12**:100267. doi:10.1016/j.lanepe.2021.100267

17 Izbicki R, Bastos LS, Izbicki M, *et al.* How many hospitalizations has the covid-19 vaccination already prevented in são paulo? *Clinics* 2021;**76**:3–4. doi:10.6061/clinics/2021/e3250

18 Mitra AK, Payton M, Kabir N, *et al.* Potential years of life lost due to COVID-19 in the United States, Italy, and Germany: An old formula with newer ideas. *Int J Environ Res Public Health* 2020;**17**:1–9. doi:10.3390/ijerph17124392

19 Dos Santos CVB, de Noronha TG, Werneck G, *et al.* Estimated COVID-19 Severe Cases and Deaths Averted in the First Year Of the Vaccination Campaign in Brazil: A Retrospective Observational Study. *Lancet Reg Heal* 2023;**17**:100418. doi:10.2139/ssrn.4151508

20 Cifuentes-Faura J. COVID-19 mortality rate and its incidence in Latin America: Dependence on demographic and economic variables. *Int J Environ Res Public Health* 2021;**18**.

21 Hotez PJ, Narayan KMV. Sharing Technology and Vaccine Doses to Address Global Vaccine Inequity and End the COVID-19 Pandemic. *JAMA - J Am Med Assoc* 2021;**326**:219–20. doi:10.1001/jama.2021.7439

22 Fonseca EM da, Shadlen KC, Bastos FI. The politics of COVID-19 vaccination in middle-income countries: Lessons from Brazil. *Soc Sci Med* 2021;**281**:114093. doi:10.1016/j.socscimed.2021.114093

23 Gramacho WG, Turgeon M. When politics collides with public health: COVID-19 vaccine country of origin and vaccination acceptance in Brazil. *Vaccine* 2021;**39**:2608–12. doi:10.1016/j.vaccine.2021.03.080

24 Moore DCBC, Nehab MF, Camacho KG, *et al.* Low COVID-19 vaccine hesitancy in Brazil. *Vaccine* 2021;**39**:6262–8. doi:10.1016/j.vaccine.2021.09.013

25 Faden R, Cravioto A, Hombach J, *et al.* Who to vaccinate first? A peek at decision-making in a pandemic. *Nature* 2022;**607**:235–8. doi:10.1038/d41586-022-01899-2

26 Watson OJ, Barnsley G, Toor J, *et al.* Global impact of the first year of COVID-19 vaccination: a mathematical modelling study. *Lancet Infect Dis* 2022;**22**:1293–302. doi:10.1016/S1473-3099(22)00320-6

27 Hogan AB, Winskill P, Watson OJ, *et al.* Within-country age-based prioritisation, global allocation, and public health impact of a vaccine against SARS-CoV-2: A mathematical modelling analysis. *Vaccine* 2021;**39**:2995–3006. doi:10.1016/j.vaccine.2021.04.002

28 Sachs JD, Karim SSA, Aknin L, *et al.* The Lancet Commission on lessons for the future from the COVID-19 pandemic. *Lancet* 2022;**400**.

For peer review only - http://bmjopen.bmj.com/site/about/guidelines.xhtml

Figure legends

Figure 1. Progression of COVID-19 reported deaths and the vaccination roll-out in Brazil: (A) Daily number of reported deaths (15-day moving average, left y-axis) and first dose vaccine coverage (right y-axis) from February 26, 2020 to December 31, 2021; yellow shaded areas display the period of VOC dominance (50% of samples using oubreak.info/GISAID data) for Gamma (February 15, 2021) and Delta (August 11, 2021) - vertical dashed lines indicate the start of the vaccination roll-out in Brazil and the 75% first vaccination coverage of adult population, respectively; (B) Daily number reported deaths stratified by age group in 2021; (C) Daily proportions of deaths reported for each age group (number of deaths / total number of deaths per day); (D) First dose vaccination coverage stratified by age group - vertical dashed lines refer to the start of the vaccination campaign for each age group, the horizontal dashed line indicates the 75% vaccination coverage target by the World Health Organization (WHO).

Figure 2. The impact of the COVID-19 vaccination campaign in reported deaths stratified by age groups: (A) Rate Ratios comparing daily mortality rates stratified by age group with the national mortality rate; (B) Estimated effect of the vaccination campaign in each age groups. Effects were obtained as the Rate Ratio (RR) and their respective 95% confidence intervals. The vertical dashed line refers to the start of the vaccination campaign on January 17, 2021. Our analysis considered six months before the vaccination roll-out as the baseline period and used the national mortality rates as reference in a Negative Binomial Regression model.

Figure 3. Estimated prevented and possible preventable deaths stratified by age group. For each age group (coloured lines), the predicted deaths (black lines) were obtained whether that age-group followed the national age-adjusted mortality rates. Grey lines correspond to the fitted linear model. Predicted deaths were estimated from the period when 10% of first dose vaccination coverage was achieved for each age group. Prevented deaths (green-shaded area) were obtained if observed deaths was lower than predicted, and possible preventable deaths (red-shaded area), if the number of observed deaths was higher than predicted. The vertical dashed lines refer to the start of the vaccination campaign on January 17, 2021.

For peer review only

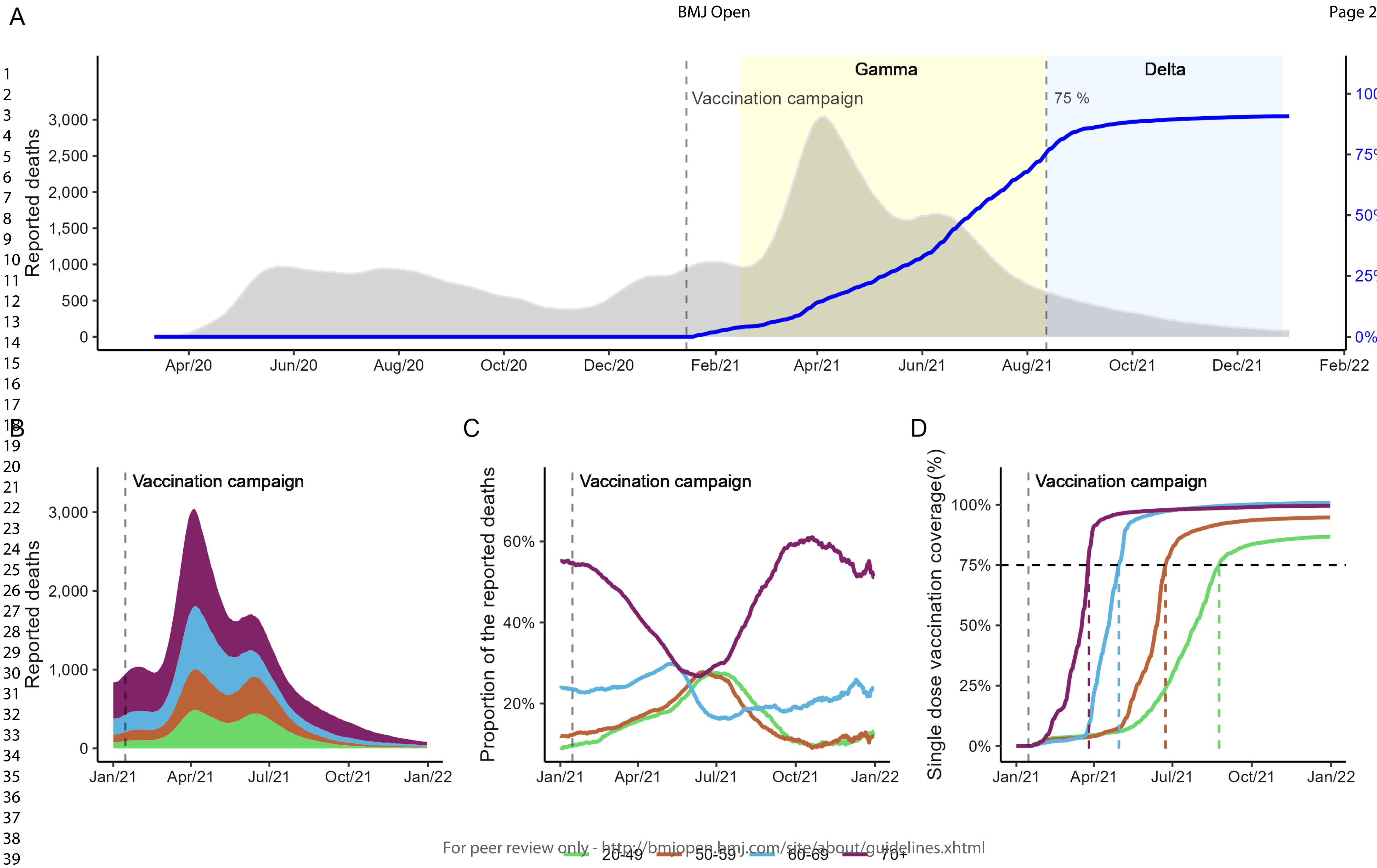

For peer review only - http://bmjopen.bmj.com/site/about/guidelines.xhtml

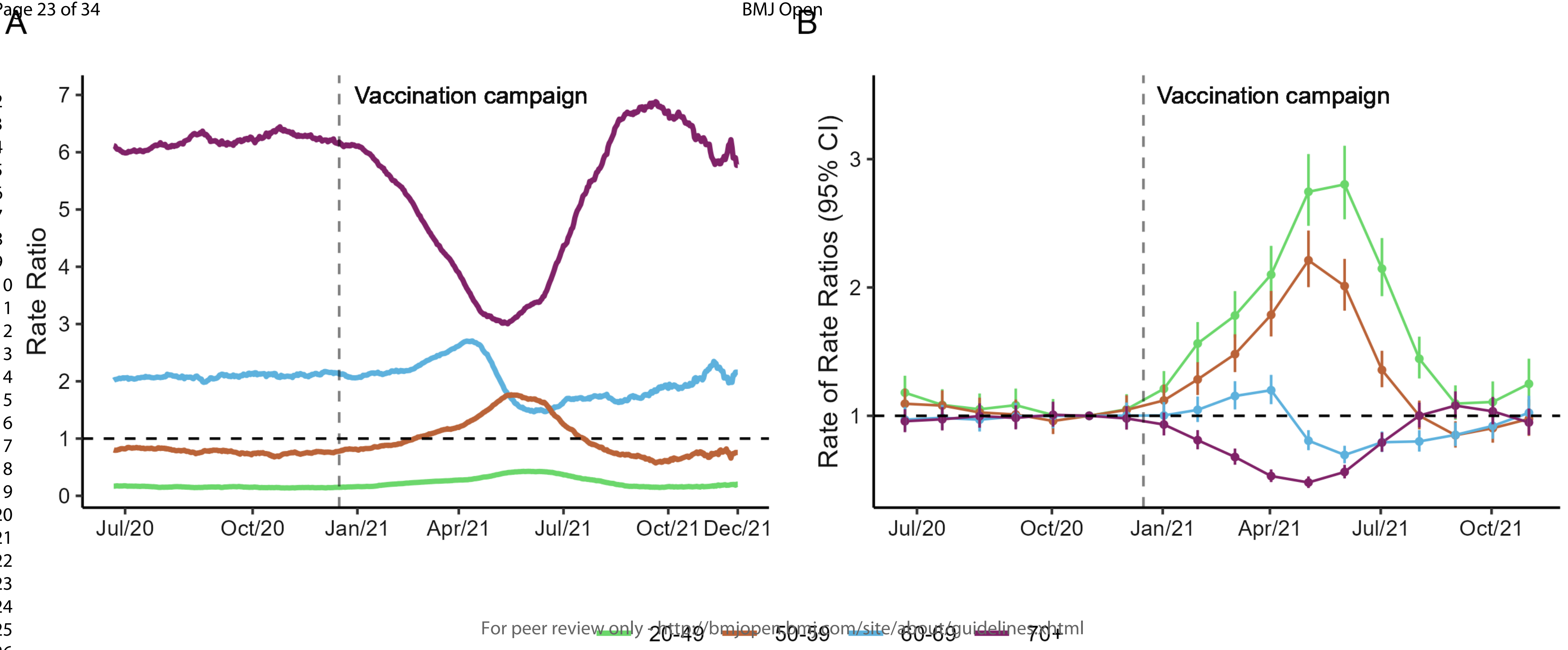

For peer review only - http://bmjopen.bmj.com/site/about/guidelines.xhtml

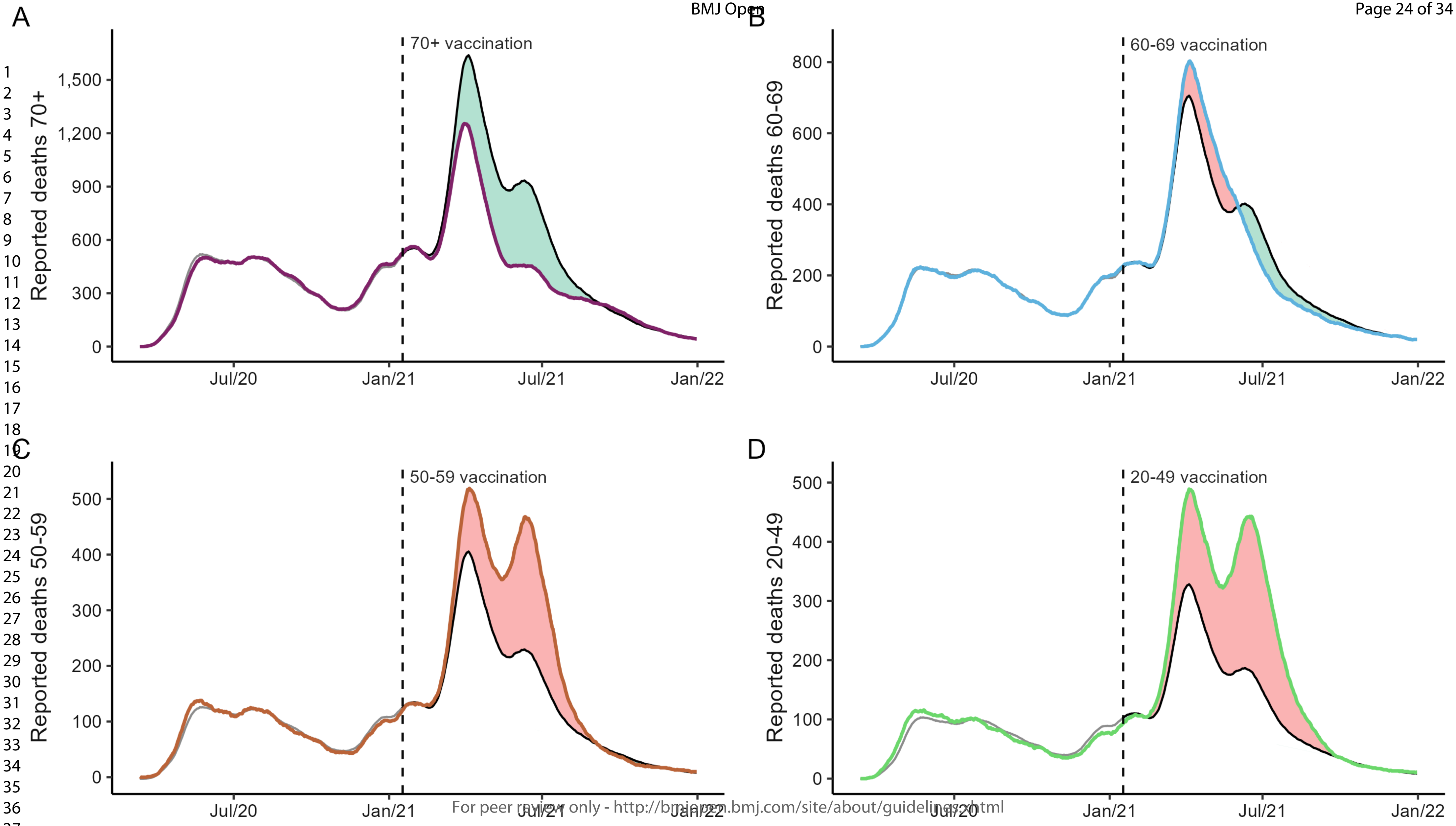
A
70+ vaccination
Reported deaths 70+
0
300
600
900
1,200
1,500
Jul/20
Jan/21
Jul/21
Jan/22
B
60-69 vaccination
Reported deaths 60-69
0
200
400
600
800
Jul/20
Jan/21
Jul/21
Jan/22
C
50-59 vaccination
Reported deaths 50-59
0
100
200
300
400
500
Jul/20
Jan/21
Jul/21
Jan/22
D
20-49 vaccination
Reported deaths 20-49
0
100
200
300
400
500
Jul/20
Jan/21
Jul/21
Jan/22

Supplementary Material

Table of contents

Figure S1. (A) Deaths per 100.000 pop stratified by age group – The vertical dashed line refers to the start of the vaccination campaign. (B) Estimated effect for each age group before and after the beginning the vaccination campaign. Effects were obtained as the Rate Ratio (RR) and their respective 95% confidence intervals. 3

Figure S2. (A) First dose vaccination coverage stratified by age group – first vertical dashed line on the left refers to the start of the vaccination campaign for each age group, the horizontal dashed line indicates the 75% vaccination coverage target by the World Health Organization (WHO). (B) Second or single vaccination coverage stratified by age group. 3

Figure S3. (A) Estimated effect of the vaccination campaign in each age groups comparing to 70+ age group. (B) Estimated effect of the vaccination campaign in each age groups comparing to 20-49 age group. Effects were obtained as the Rate Ratio (RR) and their respective 95% confidence intervals. The vertical dashed line refers to the start of the vaccination campaign on January 17, 2021. Our analysis considered six months before the vaccination roll-out as the baseline period and used the national mortality rates as reference in a Negative Binomial Regression model. 4

Table S1. Description of the data sources used in this study 2

Table S2. Monthly average of the mortality ratios in Brazil, in 2021, stratified by age group. 5

Table S3. Number of reported deaths and first dose vaccine coverage per month in during the COVID-19 vaccination campaign in Brazil (started on January 17, 2021). 6

Table S4. Second or single vaccination coverage during the vaccination roll-out in Brazil, in 2021, stratified by age group. 7

Table S5. Monthly and global estimated effect of the vaccination campaign with their respective 95% confidence intervals stratified by age group. 8

Table S6. Monthly estimated effect of the vaccination campaign with their respective 95% confidence intervals stratified by age group (70+ reference). 8

Table S7. Monthly estimated effect of the vaccination campaign with their respective 95% confidence intervals stratified by age group (20-49 reference). 8

Table S8. Monthly estimated effect of the vaccination campaign with their respective 95% confidence intervals stratified by age group. 9

For peer review only - http://bmjopen.bmj.com/site/about/guidelines.xhtml

Table S1. Description of the data sources used in this study

Data	Source	Source address	Version	Date exported
COVID-19 Vaccination doses	National Immunisation Program Information System,"SI-PNI"	*OpenDataSUS* repository: https://opendatasus.saude.gov.br/dataset/covid-19-vacinacao	21/07/2022	21/07/2022
Brazilian population estimates (per age, sex, and municipalities)	Health Surveillance Secretary from the Brazilian Ministry of Health	Official website: http://tabnet.datasus.gov.br/cgi/tabcgi.exe?popsvs/cnv/popbr.def	2020	07/02/2022
COVID-19 hospitalized cases	Influenza Epidemiological Surveillance Information System, "SIVEP-Gripe"	*OpenDataSUS* repository: https://opendatasus.saude.gov.br/dataset/bd-srag-2020 https://opendatasus.saude.gov.br/dataset/srag-2021-e-2022	21/07/2022	21/07/2022
Life expectancy at birth, total (years) - Brazil	World Bank	Official website: https://data.worldbank.org/indicator/SP.DYN.LE00.IN?locations=BR&most_recent_year_desc=true	2019	25/07/2022

COVID-19: Disease caused by the SARS-CoV-2 virus.

For peer review only - http://bmjopen.bmj.com/site/about/guidelines.xhtml

Figure S1. (A) Deaths per 100.000 pop stratified by age group – The vertical dashed line refers to the start of the vaccination campaign. (B) Estimated effect for each age group before and after the beginning the vaccination campaign. Effects were obtained as the Rate Ratio (RR) and their respective 95% confidence intervals.

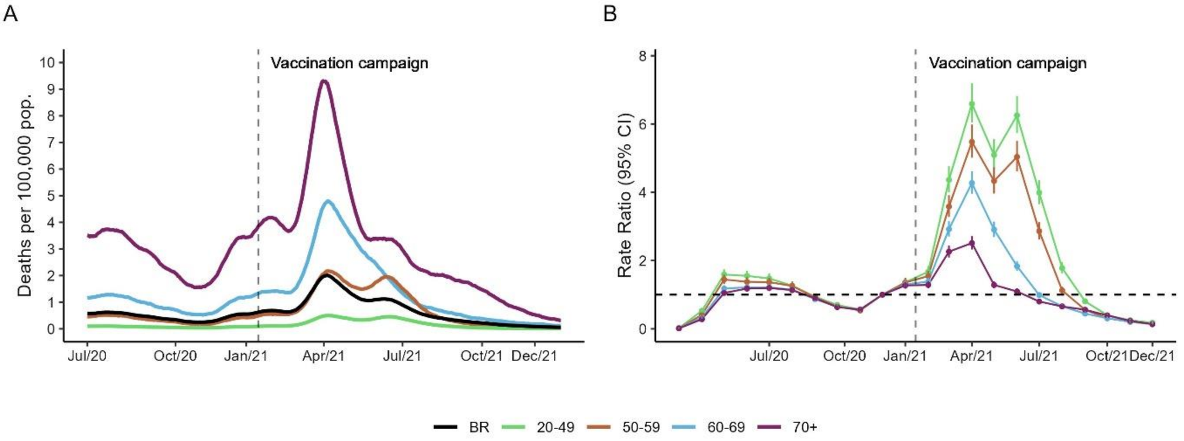

Figure S2. (A) First dose vaccination coverage stratified by age group – first vertical dashed line on the left refers to the start of the vaccination campaign for each age group, the horizontal dashed line indicates the 75% vaccination coverage target by the World Health Organization (WHO). (B) Second or single vaccination coverage stratified by age group.

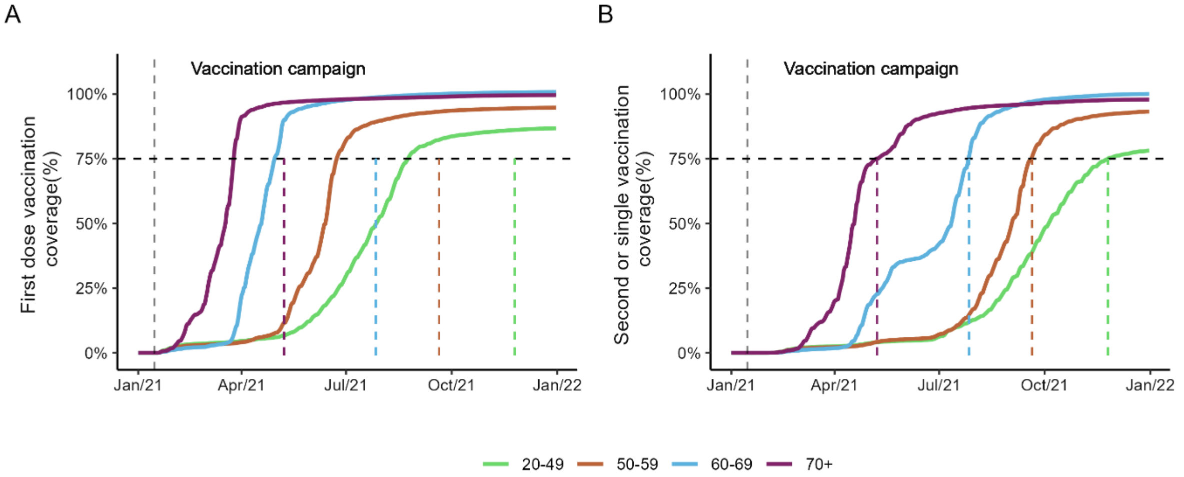

For peer review only - http://bmjopen.bmj.com/site/about/guidelines.xhtml

Figure S3. (A) Estimated effect of the vaccination campaign in each age groups comparing to 70+ age group. (B) Estimated effect of the vaccination campaign in each age groups comparing to 20-49 age group. Effects were obtained as the Rate Ratio (RR) and their respective 95% confidence intervals. The vertical dashed line refers to the start of the vaccination campaign on January 17, 2021. Our analysis considered six months before the vaccination roll-out as the baseline period and used the national mortality rates as reference in a Negative Binomial Regression model.

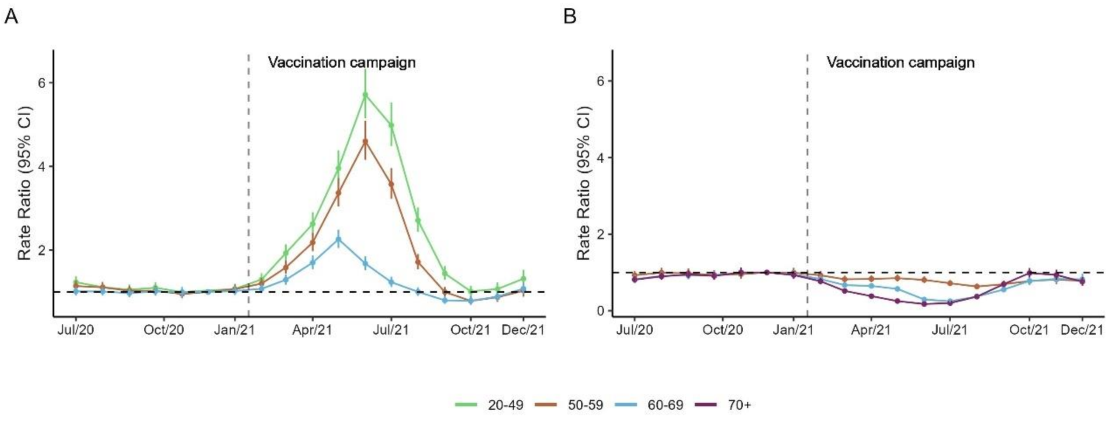

For peer review only - http://bmjopen.bmj.com/site/about/guidelines.xhtml

Table S2. Monthly average of the mortality ratios in Brazil, in 2021, stratified by age group.

Age group	Jan/21	Feb/21	Mar/21	Apr/21	Mai/21	Jun/21	Jul/21	Aug/21	Sep/21	Oct/21	Nov/21	Dec/21
20 – 49	0.15	0.17	0.22	0.26	0.31	0.41	0.41	0.31	0.2	0.16	0.16	0.18
50 – 59	0.79	0.85	0.96	1.14	1.38	1.72	1.53	1.03	0.75	0.63	0.69	0.74
60 – 69	2.12	2.12	2.22	2.48	2.58	1.74	1.52	1.7	1.7	1.81	1.95	2.16
70+	6.16	5.87	5.21	4.29	3.38	3.11	3.69	5.1	6.35	6.77	6.48	5.96

For peer review only

Table S3. Number of reported deaths and first dose vaccine coverage per month in during the COVID-19 vaccination campaign in Brazil (started on January 17, 2021).

Age group	Monthly Average Baseline*	Baseline*	Jan/21	Feb/21	Mar/21	Apr/21	Mai/21	Jun/21	Jul/21	Aug/21	Sep/21	Oct/21	Nov/21	Dec/21
Monthly reported deaths														
BR	20,627	123,763	29,346	28,141	62,603	79,939	53,772	48,416	31,293	18,485	12,164	8,254	4,958	3,223
20 – 49	2,050	12,298 (10 %)	2,842 (10 %)	3,149 (11 %)	9,099 (15 %)	13,299 (17 %)	10,617 (20 %)	12,620 (26 %)	8,316 (27 %)	3,714 (20 %)	1,626 (13 %)	832 (10 %)	506 (10 %)	372 (12 %)
50 – 59	2,545	15,269 (12 %)	3,641 (12 %)	3,748 (13 %)	9,611 (15 %)	14,224 (18 %)	11,623 (22 %)	13,082 (27 %)	7,677 (25 %)	3,022 (16 %)	1,449 (12 %)	830 (10 %)	531 (11 %)	374 (12 %)
60 – 69	4,731	28,385 (23 %)	6,845 (23 %)	6,588 (23 %)	15,385 (25 %)	21,775 (27 %)	15,319 (28 %)	9,363 (19 %)	5,210 (17 %)	3,471 (19 %)	2,276 (19 %)	1,634 (20 %)	1,063 (21 %)	770 (24 %)
70+	11,302	67,811 (55 %)	16,018 (55 %)	14,656 (52 %)	28,508 (46 %)	30,641 (38 %)	16,213 (30 %)	13,351 (28 %)	10,090 (32 %)	8,278 (45 %)	6,813 (56 %)	4,958 (60 %)	2,858 (58 %)	1,707 (53 %)
Monthly average mortality rates per 100,000 people														
BR		0.44	0.62	0.66	1.33	1.76	1.14	1.06	0.67	0.39	0.27	0.18	0.11	0.07
20 – 49		0.07	0.09	0.12	0.30	0.45	0.35	0.43	0.27	0.12	0.06	0.03	0.02	0.01
50 – 59		0.35	0.49	0.56	1.30	1.99	1.57	1.83	1.04	0.41	0.20	0.11	0.07	0.05
60 – 69		0.92	1.32	1.41	2.97	4.34	2.95	1.87	1.00	0.67	0.45	0.32	0.21	0.15
70+		2.73	3.84	3.89	6.83	7.59	3.88	3.31	2.42	1.98	1.69	1.19	0.71	0.41
Vaccine coverage[#] (%)														
BR			1.82	4.74	13.75	21.92	32.22	50.82	67.72	84.82	88.32	89.58	90.3	90.67
20 – 49			1.99	3.61	4.56	5.98	12.55	28.85	52.75	78.55	83.55	85.26	86.23	86.72
50 – 59			1.65	3.06	4.08	7.64	31.84	81.34	89.73	92.36	93.57	94.16	94.53	94.75
60 – 69			1.05	2.28	19.58	75.48	95.28	97.61	98.79	99.6	100.09	100.4	100.6	100.72
70+			1.85	18.95	90.35	96.32	97.4	97.99	98.38	98.72	99.07	99.4	99.53	99.61

*Baseline period corresponds to the six-month period (July to December 2020), prior to the start of the vaccination campaign. For monthly reported deaths, the baseline is the sum of the six months deaths.

[#]At least one dose.

For peer review only

For peer review only - http://bmjopen.bmj.com/site/about/guidelines.xhtml

Table S4. Second or single vaccination coverage during the vaccination roll-out in Brazil, in 2021, stratified by age group.

Age group	Jan/21	Feb/21	Mar/21	Apr/21	Mai/21	Jun/21	Jul/21	Aug/21	Sep/21	Oct/21	Nov/21	Dec/21
Vaccine coverage (%)												
BR	0	1.56	3.86	11.37	15.26	17.78	28.38	42.88	62.88	76.18	82.88	84.55
20 – 49	0	1.7	2.54	3.7	4.65	6.05	12.74	26.04	47.34	66.04	75.76	78.08
50 – 59	0	1.45	2.19	3.4	5.21	6.95	17.05	46.05	83.75	90.42	92.44	93.29
60 – 69	0	0.87	1.79	18.39	35.29	41.64	83.04	94.74	97.78	98.94	99.71	100
70+	0.01	1.67	19.17	72.57	85.27	92.52	94.75	95.68	96.59	97.27	97.69	97.83

For peer review only

Table S5. Monthly and global estimated effect of the vaccination campaign with their respective 95% confidence intervals stratified by age group.

Age group	Global	Jan/21	Feb/21	Mar/21	Apr/21	Mai/21	Jun/21	Jul/21	Aug/21	Sep/21	Oct/21	Nov/21	Dec/21
20 – 49	1.75 (1.44 - 2.12)	1.05 (0.95 - 1.17)	1.21 (1.09 - 1.35)	1.56 (1.41 - 1.73)	1.78 (1.61 - 1.97)	2.10 (1.90 - 2.32)	2.75 (2.48 - 3.04)	2.80 (2.53 - 3.10)	2.15 (1.93 - 2.38)	1.44 (1.29 - 1.62)	1.09 (0.97 - 1.24)	1.11 (0.97 - 1.27)	1.25 (1.08 - 1.44)
50 – 59	1.47 (1.21 - 1.78)	1.05 (0.94 - 1.16)	1.12 (1.01 - 1.24)	1.28 (1.16 - 1.42)	1.48 (1.34 - 1.64)	1.79 (1.62 - 1.97)	2.21 (2.00 - 2.44)	2.01 (1.82 - 2.22)	1.36 (1.22 - 1.51)	1.00 (0.90 - 1.12)	0.85 (0.75 - 0.96)	0.90 (0.79 - 1.03)	0.98 (0.84 - 1.13)
60 – 69	1.01 (0.84 - 1.23)	1.00 (0.91 - 1.10)	1.00 (0.91 - 1.11)	1.05 (0.95 - 1.15)	1.15 (1.05 - 1.23)	1.20 (1.09 - 1.27)	0.81 (0.73 - 1.32)	0.70 (0.63 - 0.71)	0.79 (0.72 - 0.88)	0.80 (0.72 - 0.89)	0.85 (0.76 - 0.95)	0.92 (0.82 - 1.03)	1.02 (0.91 - 1.16)
70+	0.73 (0.60 - 0.88)	0.98 (0.89 - 1.08)	0.93 (0.85 - 1.03)	0.81 (0.74 - 0.89)	0.68 (0.62 - 0.75)	0.53 (0.48 - 0.58)	0.48 (0.43 - 0.53)	0.56 (0.51 - 0.62)	0.79 (0.72 - 0.87)	1.00 (0.91 - 1.10)	1.08 (0.98 - 1.19)	1.04 (0.93 - 1.15)	0.95 (0.85 - 1.06)

Table S6. Monthly estimated effect of the vaccination campaign with their respective 95% confidence intervals stratified by age group (70+ reference).

Age group	Jan/21	Feb/21	Mar/21	Apr/21	Mai/21	Jun/21	Jul/21	Aug/21	Sep/21	Oct/21	Nov/21	Dec/21
20 – 49	1.07 (0.96 - 1.19)	1.30 (1.16 - 1.45)	1.93 (1.74 - 2.14)	2.62 (2.37 - 2.91)	3.96 (3.57 - 4.39)	5.71 (5.15 - 6.34)	4.98 (4.49 - 5.53)	2.71 (2.44 - 3.02)	1.44 (1.29 - 1.62)	1.01 (0.90 - 1.15)	1.07 (0.93 - 1.23)	1.32 (1.13 - 1.53)
50 – 59	1.07 (0.96 - 1.19)	1.20 (1.08 - 1.34)	1.58 (1.43 - 1.75)	2.18 (1.97 - 2.41)	3.36 (3.05 - 3.72)	4.60 (4.16 - 5.09)	3.57 (3.23 - 3.96)	1.71 (1.54 - 1.91)	1.00 (0.89 - 1.12)	0.79 (0.70 - 0.89)	0.87 (0.76 - 1.00)	1.03 (0.89 - 1.19)
60 – 69	1.02 (0.93 - 1.13)	1.08 (0.97 - 1.19)	1.29 (1.17 - 1.42)	1.70 (1.54 - 1.87)	2.26 (2.05 - 2.49)	1.68 (1.52 - 1.85)	1.24 (1.18 - 1.37)	1.00 (0.91 - 1.11)	0.80 (0.72 - 0.89)	0.79 (0.71 - 0.88)	0.89 (0.79 - 1.00)	1.08 (0.95 - 1.23)
70+ (REF)	-	-	-	-	-	-	-	-	-	-	-	-

Table S7. Monthly estimated effect of the vaccination campaign with their respective 95% confidence intervals stratified by age group (20-49 reference).

Age group	Jan/21	Feb/21	Mar/21	Apr/21	Mai/21	Jun/21	Jul/21	Aug/21	Sep/21	Oct/21	Nov/21	Dec/21
20 – 49 (REF)	-	-	-	-	-	-	-	-	-	-	-	-
50 – 59	0.99 (0.89 - 1.19)	0.92 (0.82 - 1.04)	0.82 (0.74 - 0.92)	0.83 (0.75 - 0.93)	0.85 (0.76 - 0.95)	0.81 (0.72 - 0.90)	0.71 (0.64 - 0.80)	0.63 (0.56 - 0.71)	0.69 (0.61 - 0.79)	0.77 (0.67 - 0.89)	0.81 (0.69 - 0.96)	0.78 (0.65 - 0.93)
60 – 69	0.95 (0.85 - 1.07)	0.82 (0.74 - 0.93)	0.67 (0.60 - 0.74)	0.65 (0.58 - 0.72)	0.57 (0.51 - 0.63)	0.29 (0.26 - 0.33)	0.24 (0.22 - 0.28)	0.37 (0.33 - 0.41)	0.55 (0.49 - 0.63)	0.78 (0.68 - 0.89)	0.83 (0.72 - 0.96)	0.82 (0.70 - 0.96)
70+	0.93 (0.84 - 1.04)	0.76 (0.69 - 0.86)	0.52 (0.47 - 0.57)	0.38 (0.34 - 0.42)	0.25 (0.23 - 0.28)	0.18 (0.16 - 0.19)	0.20 (0.18 - 0.22)	0.37 (0.33 - 0.41)	0.69 (0.62 - 0.78)	0.99 (0.87 - 1.12)	0.93 (0.81 - 1.07)	0.75 (0.65 - 0.88)

For peer review only

For peer review only - http://bmjopen.bmj.com/site/about/guidelines.xhtml

Table S8. Monthly estimated effect of the vaccination campaign with their respective 95% confidence intervals stratified by age group.

Age group	Jan/21	Feb/21	Mar/21	Apr/21	Mai/21	Jun/21	Jul/21	Aug/21	Sep/21	Oct/21	Nov/21	Dec/21
20 – 49	1.36 (1.24 - 1.50)	1.67 (1.52 - 1.84)	4.36 (4.00 - 4.76)	6.59 (6.04 - 7.19)	5.09 (4.67 - 5.56)	6.25 (5.73 - 6.83)	3.99 (3.68 - 4.35)	1.78 (1.63 - 1.95)	0.81 (0.73 - 0.89)	0.40 (0.36 - 0.44)	0.25 (0.22 - 0.28)	0.18 (0.16 - 0.20)
50 – 59	1.35 (1.23 - 1.49)	1.55 (1.41 - 1.70)	3.58 (3.28 - 3.91)	5.48 (5.01 - 5.99)	4.33 (3.97 - 4.73)	5.04 (4.61 - 5.51)	2.86 (2.62 - 3.13)	1.13 (1.03 - 1.24)	0.56 (0.50 - 0.62)	0.31 (0.28 - 0.35)	0.20 (0.18 - 0.23)	0.14 (0.12 - 0.15)
60 – 69	1.29 (1.20 - 1.41)	1.38 (1.27 - 1.50)	2.92 (2.70 - 3.15)	4.27 (3.96 - 4.61)	2.91 (2.69 - 3.14)	1.84 (1.70 - 1.99)	0.99 (0.91 - 1.07)	0.66 (0.61 - 0.72)	0.45 (0.41 - 0.49)	0.31 (0.28 - 0.34)	0.21 (0.19 - 0.23)	0.15 (0.13 - 0.16)
70+	1.28 (1.18 - 1.37)	1.29 (1.19 - 1.39)	2.26 (2.09 - 2.44)	2.51 (2.32 - 2.72)	1.29 (1.19 - 1.39)	1.09 (1.01 - 1.18)	0.80 (0.74 - 0.87)	0.66 (0.61 - 0.71)	0.56 (0.52 - 0.61)	0.39 (0.36 - 0.43)	0.23 (0.22 - 0.26)	0.14 (0.12 - 0.15)

For peer review only

STROBE 2007 (v4) Statement—Checklist of items that should be included in reports of cross-sectional studies.

Section/Topic	Item No	Recommendation	Reported on page #
**Title and abstract**	1	(*a*) Indicate the study's design with a commonly used term in the title or the abstract	Title, Page 1
		(*b*) Provide in the abstract an informative and balanced summary of what was done and what was found	Abstract, Methods/Findings, Page 2
**Introduction**			
Background/rationale	2	Explain the scientific background and rationale for the investigation being reported	Page 4
Objectives	3	State specific objectives, including any prespecified hypotheses	Page 4
**Methods**			
Study design	4	Present key elements of study design early in the paper	Methods, Pages 4-8, Table S1
Setting	5	Describe the setting, locations, and relevant dates, including periods of recruitment, exposure, follow-up, and data collection	Pages 4-8
Participants	6	(*a*) *Cross-sectional study*—Give the eligibility criteria, and the sources and methods of selection of participants	Page 4-5
Variables	7	Clearly define all outcomes, exposures, predictors, potential confounders, and effect modifiers. Give diagnostic criteria, if applicable	Pages 6-7, Figure S1
Data sources/ measurement	8*	For each variable of interest, give sources of data and details of methods of assessment (measurement). Describe comparability of assessment methods if there is more than one group	Methods, Study design and Participants, Pages 5-7, Table S1
Bias	9	Describe any efforts to address potential sources of bias	Methods, Pages 6-8
Study size	10	Explain how the study size was arrived at	Methods, Data analysis, Pages 6-8
Quantitative variables	11	Explain how quantitative variables were handled in the analyses. If applicable, describe which groupings were chosen and why	Methods, Data analysis, Pages 6-8, Table S3
Statistical methods	12	(*a*) Describe all statistical methods, including those used to control for confounding	Methods, Data analysis, Pages 6-8
		(*b*) Describe any methods used to examine subgroups and interactions	Methods, Data analysis, Pages 6-8
		(*c*) Explain how missing data were addressed	Methods, Data analysis, Pages 6-8
		(*d*) *Cross-sectional study*—If applicable, describe analytical methods taking account of sampling strategy	NA
		(*e*) Describe any sensitivity analyses	Methods, Data analysis, Pages 7 Figure S3, Tables S7 and S8

For peer review only

**Results**			
Participants	13*	(a) Report numbers of individuals at each stage of study—eg numbers potentially eligible, examined for eligibility, confirmed eligible, included in the study, completing follow-up, and analysed	Results, Page 8
		(b) Give reasons for non-participation at each stage	NA
		(c) Consider use of a flow diagram	NA
Descriptive data	14*	(a) Give characteristics of study participants (eg demographic, clinical, social) and information on exposures and potential confounders	Results, Page 8, Table 1
		(b) Indicate number of participants with missing data for each variable of interest	Results, Page 8, Table 1
		(c) *Cohort study*—Summarise follow-up time (eg, average and total amount)	NA
Outcome data	15*	*Cross-sectional study*—Report numbers of outcome events or summary measures	Results, Pages 8-11
Main results	16	(*a*) Give unadjusted estimates and, if applicable, confounder-adjusted estimates and their precision (eg, 95% confidence interval). Make clear which confounders were adjusted for and why they were included	Results, Pages 8-11, Figure 2-3, Figure S1, Tables S2-S4
		(*b*) Report category boundaries when continuous variables were categorized	Results, Pages 8-11, Table 1
		(*c*) If relevant, consider translating estimates of relative risk into absolute risk for a meaningful time period	NA
Other analyses	17	Report other analyses done—eg analyses of subgroups and interactions, and sensitivity analyses	Results, Pages 9-11, Figure 2, Figure 3,
**Discussion**			
Key results	18	Summarise key results with reference to study objectives	Discussion, Pages 11-12
Limitations	19	Discuss limitations of the study, taking into account sources of potential bias or imprecision. Discuss both direction and magnitude of any potential bias	Discussion, Pages 12-13
Interpretation	20	Give a cautious overall interpretation of results considering objectives, limitations, multiplicity of analyses, results from similar studies, and other relevant evidence	Discussion, Pages 11-13
Generalisability	21	Discuss the generalisability (external validity) of the study results	Discussion, Pages 12-13
**Other information**			
Funding	22	Give the source of funding and the role of the funders for the present study and, if applicable, for the original study on which the present article is based	Page 16

*Give information separately for cases and controls in case-control studies and, if applicable, for exposed and unexposed groups in cohort and cross-sectional studies.

Note: An Explanation and Elaboration article discusses each checklist item and gives methodological background and published examples of transparent reporting. The STROBE checklist is best used in conjunction with this article (freely available on the Web sites of PLoS Medicine at http://www.plosmedicine.org/, Annals of Internal Medicine at http://www.annals.org/, and Epidemiology at http://www.epidem.com/). Information on the STROBE Initiative is available at www.strobe-statement.org.

For peer review only
